# Supplementary material for: Metal-free alcohol-directed regioselective heteroarylation of remote unactivated C(sp3)–H bonds
Source: Nat Commun. 2018 Aug 21;9:3343. doi: 10.1038/s41467-018-05522-9 (PMC6104081; doi:10.1038/s41467-018-05522-9)
Supplement: Supplementary file 1 — Supplementary Information [file 41467_2018_5522_MOESM1_ESM.pdf]

Supplementary Information

Metal-Free Alcohol-Directed Regioselective Heteroarylation of Remote Unactivated C(sp^3)-H Bonds

Wu et al.

Supplementary Methods

All reactions were maintained under a nitrogen atmosphere unless otherwise stated. Commercially available reagents were used without further purification. Infrared (FT-IR) spectra were recorded on a BRUKER VERTEX 70, ν_{max} in cm^{-1}. ^{1}H-NMR spectra were recorded on a BRUKER AVANCE III HD (400 MHz) spectrometer. Chemical shifts are reported in ppm from tetramethylsilane with the solvent resonance as internal standard ($CDCl_3$: δ 7.26). Data are reported as follows: chemical shift, multiplicity (s = singlet, d = doublet, t = triplet, q = quadruplet, br = broad, m = multiplet), coupling constants (Hz) and integration. ^{13}C-NMR spectra were recorded on a BRUKER AVANCE III HD (100 MHz) spectrometer with complete proton decoupling. Chemical shifts are reported in ppm from tetramethylsilane with the solvent resonance as the internal standard ($CDCl_3$: δ 77.16). ^{19}F-NMR spectra were recorded on a BRUKER AVANCE III HD (376 MHz) spectrometer. Mass spectra were measured with an Agilent Technologies 6120 Quadrupole LC/MS. High resolution mass spectrometry (HRMS) were measured with a GCT Premier™ and BRUKER micrOTF-Q III. Melting points were measured using INESA WRR and values are uncorrected.

General procedure for the remote C(sp^3)-H bond heteroarylation. Heteroaryl **1** (0.4 mmol) and alcohol **2** (2.0 mmol) were loaded in a reaction vial which was subjected to evacuation/ flushing with N_2 three times. Then DCM (2.0 mL) followed by PIFA (0.92 mmol) was added to the mixture. The reaction was irradiated with 100 W blue LEDs and kept at rt under fan cooling (see the photos below). After the reaction completion monitored by TLC, the mixture was quenched by addition of aq. KOH until pH>8 and then extracted with ethyl acetate (3 x 10 mL). The combined organic extracts were washed by brine, dried over Na_2SO_4, filtered, concentrated, and purified by flash column chromatography on silica gel (eluent: ethyl acetate/ petroleum ether) to give the desired product **3**.

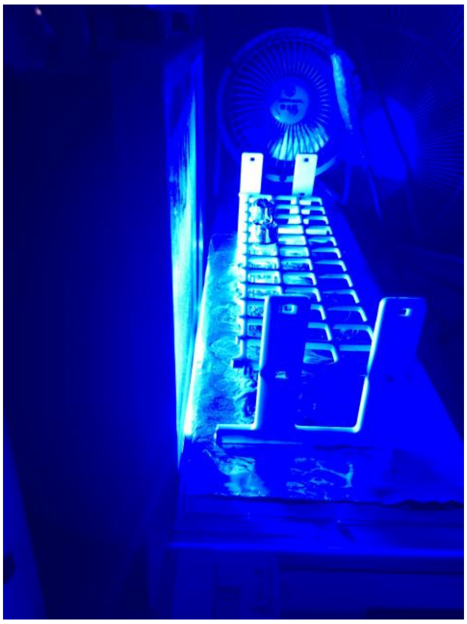

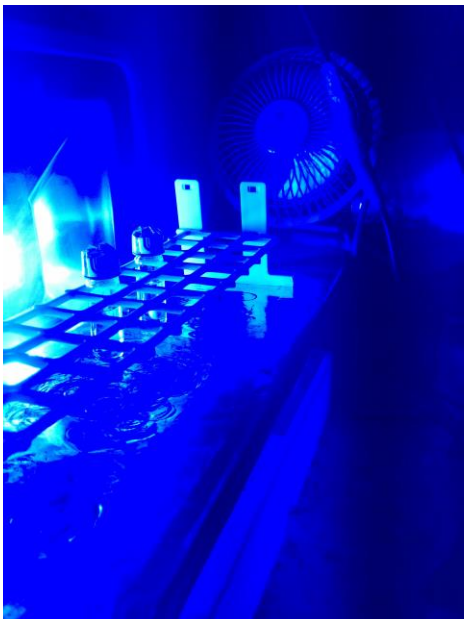

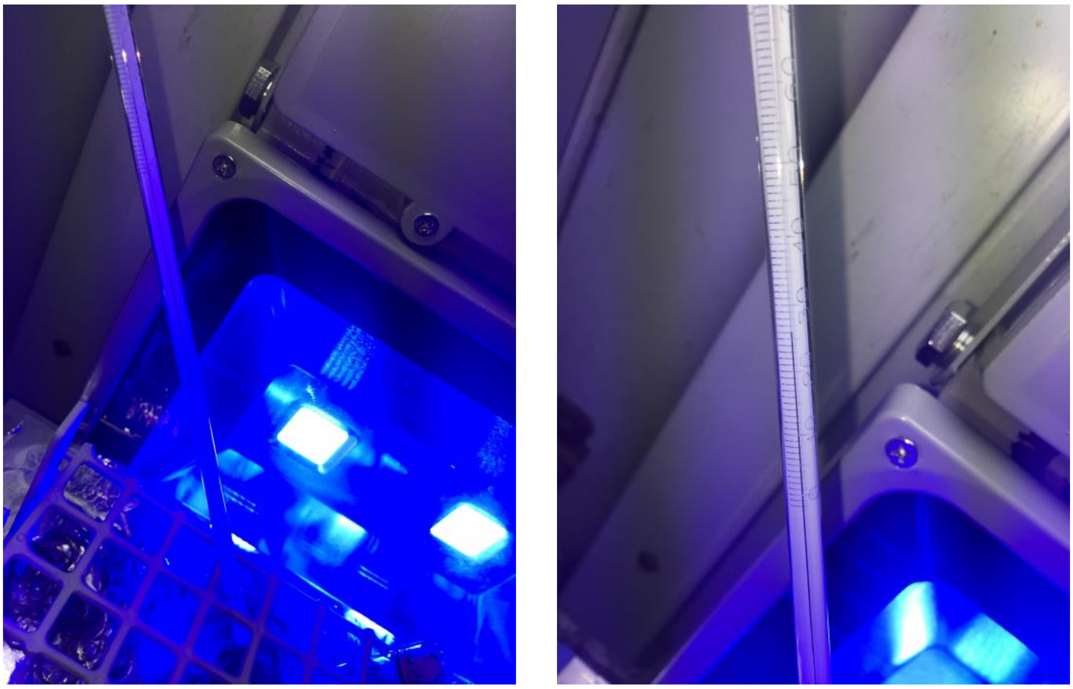

Supplementary Figure 1. Reaction equipment

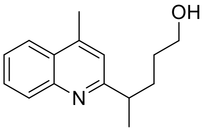

3a: yellow oil. ^{1}H NMR (400 MHz, $CDCl_3$) δ 8.03 (d, J = 8.4 Hz, 1H), 7.92 (dd, J = 8.0, 0.8 Hz, 1H), 7.64 (ddd, J = 8.0, 6.8, 1.2 Hz, 1H), 7.48 (ddd, J = 8.4, 6.8, 1.2 Hz, 1H), 7.13 (s, 1H), 3.66-3.55 (m, 2H), 3.22 (br, 1H), 3.14-3.03 (m, 1H), 2.66 (s, 3H), 1.97-1.86 (m, 1H), 1.80-1.69 (m, 1H), 1.68-1.55 (m, 1H), 1.53-1.40 (m, 1H), 1.35 (d, J = 6.8 Hz, 3H); ^{13}C NMR (100 MHz, $CDCl_3$) δ 165.8, 146.8, 144.2, 128.7, 128.6, 126.5, 125.1, 123.1, 119.7, 61.9, 41.7, 32.5, 30.2, 20.5, 18.4. FT-IR: ν (cm^{-1}) 3307, 2931, 2867, 1736, 1602, 1561, 1509, 1448, 1377, 1343, 1240. HRMS [ESI] calcd for $C_{15}H_{20}NO$ $[M+H]^+$ 230.1539, found 230.1540.

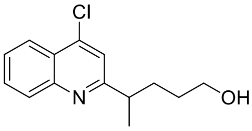

3b: yellow oil. ^{1}H NMR (400 MHz, $CDCl_3$) δ 8.12 (dd, J = 8.4, 0.8 Hz, 1H), 8.01 (d, J = 8.4 Hz, 1H), 7.67 (ddd, J = 8.4, 6.8, 1.2 Hz, 1H), 7.52 (ddd, J = 8.0, 6.8, 0.8 Hz, 1H), 7.36 (s, 1H), 3.59 (t, J = 6.4 Hz, 2H), 3.33 (s, 1H), 3.11-3.01 (m, 1H), 1.92-1.82 (m, 1H), 1.77-1.67 (m, 1H), 1.64-1.53 (m, 1H), 1.50-1.39 (m, 1H), 1.32 (d, J = 6.8 Hz, 3H); ^{13}C NMR (100 MHz, $CDCl_3$) 166.7, 148.3, 143.0, 130.3, 129.0, 126.8, 125.1, 123.9, 119.7, 62.2, 42.2, 33.0, 30.6, 20.7. FT-IR: ν (cm^{-1}) 2959, 2932, 2869, 2361, 2342, 1615, 1589, 1553. HRMS [ESI] calcd for $C_{14}H_{17}ClNO$ $[M+H]^+$ 250.0993, found 250.0995.

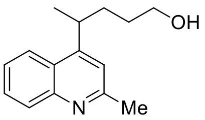

3c: yellow oil. ^{1}H NMR (400 MHz, $CDCl_3$) δ 8.01-7.95 (m, 2H), 7.58 (ddd, J = 8.4, 6.8, 1.2 Hz, 1H), 7.41 (ddd, J = 8.0, 6.8, 0.8 Hz, 1H), 7.11 (s, 1H), 3.68 (br, 1H), 3.60 (t, J = 6.4 Hz, 2H), 3.57-3.46 (m, 1H), 2.63 (s, 3H), 1.88-1.66 (m, 2H), 1.64-1.43 (m, 2H), 1.30 (d, J = 6.8 Hz, 3H); ^{13}C NMR (100 MHz, $CDCl_3$) δ 158.1, 153.2, 147.4, 128.6, 128.5, 125.0, 122.3, 118.0, 61.7, 33.0, 32.6, 30.2, 24.7, 20.7. FT-IR: ν (cm^{-1}) 2963, 2934, 2862, 1598, 1562, 1509, 1458, 1415. HRMS [ESI] calcd for

$C_{15}H_{20}NO$ [M+H]$^+$ 230.1539, found 230.1536.

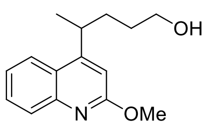

3d: yellow oil. ^{1}H NMR (400 MHz, $CDCl_3$) δ 7.95 (d, *J* = 8.0 Hz, 1H), 7.88 (dd, *J* = 8.4, 0.8 Hz, 1H), 7.60 (ddd, *J* = 8.0, 6.8, 1.2 Hz, 1H), 7.38 (ddd, *J* = 8.4, 7.2, 1.2 Hz, 1H), 6.80 (s, 1H), 4.06 (s, 3H), 3.60 (t, *J* = 6.4 Hz, 2H), 3.55-3.45 (m, 1H), 2.26 (br, 1H), 1.90-1.78 (m, 1H), 1.78-1.67 (m, 1H), 1.64-1.48 (m, 2H), 1.35 (d, *J* = 6.8 Hz, 3H); ^{13}C NMR (100 MHz, $CDCl_3$) δ 162.6, 155.9, 147.2, 129.1, 128.1, 124.3, 123.8, 123.0, 109.2, 62.7, 53.2, 33.2, 33.2, 30.6, 21.1. FT-IR: ν (cm^{-1}) 3348, 3070, 2940, 2869, 2361, 2341, 1717, 1606. HRMS [ESI] calcd for $C_{15}H_{20}NO_2$ [M+H]$^+$ 246.1489, found 246.1485.

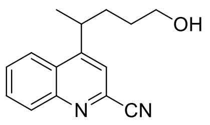

3e: yellow oil. ^{1}H NMR (400 MHz, $CDCl_3$) δ 8.17-8.12 (m, 2H), 7.79 (ddd, *J* = 8.4, 6.8, 1.2 Hz, 1H), 7.69 (ddd, *J* = 8.4, 6.8, 1.2 Hz, 1H), 7.58 (s, 1H), 3.71-3.62 (m, 3H), 1.95-1.75 (m, 3H), 1.67-1.57 (m, 1H), 1.57-1.46 (m, 1H), 1.40 (d, *J* = 6.8 Hz, 3H); ^{13}C NMR (100 MHz, $CDCl_3$) δ 155.5, 147.9, 133.1, 130.4, 130.2, 128.7, 127.3, 122.6, 119.7, 117.4, 61.9, 33.0, 32.9, 30.0, 20.6. FT-IR: ν (cm^{-1}) 3367, 2935, 2871, 2235, 1614, 1584, 1551, 1457, 1370, 1229. HRMS [ESI] calcd for $C_{15}H_{17}N_2O$ [M+H]$^+$ 241.1335, found 241.1330.

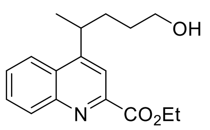

3f: yellow oil. ^{1}H NMR (400 MHz, $CDCl_3$) δ 8.27 (dd, *J* = 8.8, 0.8 Hz, 1H), 8.08 (d, *J* = 8.4 Hz, 1H), 8.03 (s, 1H), 7.69 (ddd, *J* = 8.0, 6.8, 0.8 Hz, 1H), 7.58 (ddd, *J* = 8.4, 6.8, 1.2 Hz, 1H), 4.50 (q, *J* = 7.2 Hz, 2H), 3.66-3.56 (m, 3H), 2.38 (s, 1H), 1.92-1.70 (m, 2H), 1.63-1.47 (m, 2H), 1.44 (t, *J* = 7.2 Hz, 3H), 1.36 (d, *J* = 7.2 Hz, 3H); ^{13}C NMR (100 MHz, $CDCl_3$) δ 165.3, 154.7, 147.4, 147.3, 131.1, 129.2, 127.8, 127.7, 122.4, 117.0, 62.0, 61.7, 33.0, 32.9, 30.1, 20.7, 13.9. FT-IR: ν (cm^{-1}) 2965, 2936, 2872, 2359, 2339, 2236, 1718, 1509. HRMS [ESI] calcd for $C_{17}H_{22}NO_3$ [M+H]$^+$ 288.1594, found 288.1597.

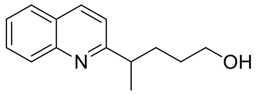

3g-*o*: yellow oil. ^{1}H NMR (400 MHz, $CDCl_3$) δ 8.08 (d, *J* = 8.8 Hz, 1H), 8.04 (d, *J* = 8.8 Hz, 1H), 7.78-7.75 (m, 1H), 7.67 (ddd, *J* = 8.4, 6.8, 1.6 Hz, 1H), 7.48 (ddd, *J* = 8.0, 6.8, 1.2 Hz, 1H), 7.31 (d, *J* = 8.4 Hz, 1H), 3.67-3.56 (m, 2H), 3.19-3.10 (m, 1H), 2.77 (br, 1H), 1.99-1.89 (m, 1H), 1.82-1.72 (m, 1H), 1.68-1.57 (m, 1H), 1.53-1.40 (m, 1H), 1.37 (d, *J* = 6.8 Hz, 3H); ^{13}C NMR (100 MHz, $CDCl_3$) δ 166.1, 147.0, 136.2, 129.0, 128.3, 127.0, 126.5, 125.3, 119.2, 62.1, 41.8, 32.5, 30.2, 20.6. FT-IR: ν (cm^{-1}) 3059, 2959, 2932, 2868, 1717, 1619, 1601, 1562. HRMS [ESI] calcd for $C_{14}H_{18}NO$ [M+H]$^+$ 216.1383, found 216.1384.

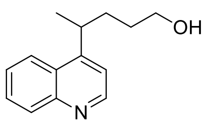

3g-*p*: yellow oil. ^{1}H NMR (400 MHz, $CDCl_3$) δ 8.78 (d, *J* = 4.4 Hz, 1H), 8.12-8.08 (m, 2H), 7.68 (ddd, *J* = 8.0, 6.8, 1.2 Hz, 1H), 7.54 (ddd, *J* = 8.4, 7.2, 1.6 Hz, 1H), 7.26 (d, *J* = 4.8 Hz, 1H), 3.36 (t, *J* = 6.4 Hz, 2H), 3.67-3.58 (m, 1H), 2.48 (br, 1H), 1.95-1.74 (m, 2H), 1.66-1.48 (m, 2H), 1.38 (d, *J* = 6.8 Hz, 3H); ^{13}C NMR (100 MHz, $CDCl_3$) δ 153.6, 150.1, 148.2, 130.2, 129.0, 127.2, 126.3, 123.0, 117.6, 62.6, 33.4, 33.2, 30.7, 21.3. FT-IR: ν (cm^{-1}) 3274, 2960, 2932, 2864, 1721, 1588, 1572, 1509. HRMS [ESI] calcd for $C_{14}H_{18}NO$ [M+H]$^+$ 216.1383, found 216.1383.

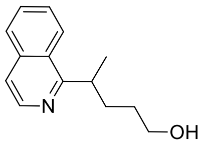

3h: yellow oil. ^{1}H NMR (400 MHz, $CDCl_3$) δ 8.43 (d, *J* = 5.6 Hz, 1H), 8.20 (d, *J* = 8.4 Hz, 1H), 7.78 (d, *J* = 8.0 Hz, 1H), 7.63 (ddd, *J* = 8.0, 6.8, 0.8 Hz, 1H), 7.55 (ddd, *J* = 8.0, 6.8, 1.2 Hz, 1H), 7.45 (d, *J* = 5.6 Hz, 1H), 3.85-3.75 (m, 1H), 3.58-3.46 (m, 2H), 2.77 (br, 1H), 2.18-2.08 (m, 1H), 1.85-1.75 (m, 1H), 1.68-1.56 (m, 1H), 1.53-1.42 (m, 1H), 1.38 (d, *J* = 6.8 Hz, 3H); ^{13}C NMR (100 MHz, $CDCl_3$) δ 165.5, 141.7, 136.4, 129.7, 127.6, 127.0, 126.8, 124.7, 119.1, 62.6, 36.0, 32.1, 31.0, 21.2. FT-IR: ν (cm^{-1}) 3308, 3053, 2932, 2869, 1693, 1622, 1588, 1561. HRMS [ESI] calcd for $C_{14}H_{18}NO$ $[M+H]^+$ 216.1383, found 216.1379.

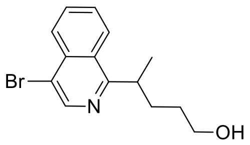

3i: yellow oil. ^{1}H NMR (400 MHz, $CDCl_3$) δ 8.62 (s, 1H), 8.19 (d, *J* = 7.6 Hz, 1H), 8.15 (d, *J* = 8.4 Hz, 1H), 7.74 (ddd, *J* = 8.4, 7.2, 1.2 Hz, 1H), 7.62 (ddd, *J* = 8.4, 6.8, 1.2 Hz, 1H), 3.81-3.71 (m, 1H), 3.60-3.50 (m, 2H), 2.69 (br, 1H), 2.13-2.03 (m, 1H), 1.82-1.72 (m, 1H), 1.63-1.52 (m, 1H), 1.50-1.40 (m, 1H), 1.36 (d, *J* = 6.8 Hz, 3H); ^{13}C NMR (100 MHz, $CDCl_3$) δ 164.7, 143.0, 134.4, 130.5, 127.6, 127.5, 126.4, 124.6, 117.2, 62.1, 35.5, 31.7, 30.4, 20.6. FT-IR: ν (cm^{-1}) 3072, 3045, 2932, 2869, 1717, 1616, 1566, 1557. HRMS [ESI] calcd for $C_{14}H_{17}BrNO$ $[M+H]^+$ 294.0488, found 294.0486.

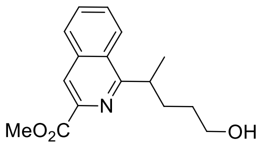

3j: yellow oil. ^{1}H NMR (400 MHz, $CDCl_3$) δ 8.37 (s, 1H), 8.26-8.22 (m, 1H), 7.92-7.89 (m, 1H), 7.73-7.66 (m, 2H), 3.98 (s, 3H), 3.83-3.73 (m, 1H), 3.58-3.51 (m, 1H), 3.46-3.38 (m, 1H), 2.71 (br, 1H), 2.44-2.32 (m, 1H), 1.82-1.75 (m, 1H), 1.75-1.65 (m, 1H), 1.51-1.41 (m, 1H), 1.39 (d, *J* = 6.8 Hz, 3H); ^{13}C NMR (100 MHz, $CDCl_3$) δ 166.1, 165.3, 139.8, 135.6, 129.9, 128.8, 128.6, 127.6, 124.5, 122.2, 61.6, 52.2, 36.4, 30.6, 30.2, 21.2. FT-IR: ν (cm^{-1}) 3417, 2949, 2934, 2870, 2360, 2343, 2237, 1721. HRMS [ESI] calcd for $C_{16}H_{20}NO_3$ $[M+H]^+$ 274.1438, found 274.1442.

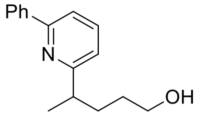

3k-*o*: yellow oil. ^{1}H NMR (400 MHz, $CDCl_3$) δ 8.04-7.98 (m, 2H), 7.66 (t, *J* = 7.6 Hz, 1H), 7.53 (dd, *J* = 8.0, 0.8 Hz, 1H), 7.49-7.43 (m, 2H), 7.42-7.36 (m, 1H), 7.08 (dd, *J* = 7.6, 0.8 Hz, 1H), 3.61 (td, *J* = 6.4, 2.0 Hz, 2H), 3.06-2.95 (m, 1H), 2.59 (br, 1H), 1.95-1.84 (m, 1H), 1.76-1.66 (m, 1H), 1.63-1.54 (m, 1H), 1.53-1.46 (m, 1H), 1.35 (d, *J* = 7.2 Hz, 3H); ^{13}C NMR (100 MHz, $CDCl_3$) δ 165.5, 156.0, 139.2, 136.6, 128.3, 128.2, 126.5, 119.3, 117.4, 62.4, 41.1, 32.8, 30.2, 20.6. FT-IR: ν (cm^{-1}) 2958, 2931, 2868, 1720, 1590, 1570, 1461, 1445. HRMS [ESI] calcd for $C_{16}H_{20}NO$ $[M+H]^+$ 242.1539, found 242.1539.

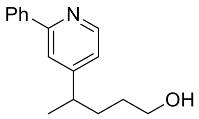

3k-*p*: yellow oil. ^{1}H NMR (400 MHz, $CDCl_3$) δ 8.56 (d, *J* = 5.2 Hz, 1H), 7.97-7.92 (m, 2H), 7.52 (s, 1H), 7.49-7.43 (m, 2H), 7.43-7.37 (m, 1H), 7.06 (dd, *J* = 5.2, 1.6 Hz, 1H), 3.60 (t, *J* = 6.4 Hz, 2H), 2.81-2.70 (m, 1H), 1.73-1.65 (m, 2H), 1.59-1.49 (m, 1H), 1.47-1.41 (m, 1H), 1.29 (d, *J* = 6.8 Hz, 3H); ^{13}C NMR (100 MHz, $CDCl_3$) δ 157.1, 156.7, 149.1, 139.1, 128.4, 128.2, 126.6, 120.6, 119.3, 62.2, 39.1, 33.3, 30.2, 21.1. FT-IR: ν (cm^{-1}) 3321, 3059, 2958, 2930, 2869, 2361, 2341, 1719. HRMS [ESI] calcd for $C_{16}H_{20}NO$ $[M+H]^+$ 242.1539, found 242.1537.

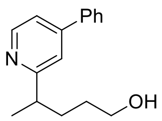

3l: yellow oil. ^{1}H NMR (400 MHz, $CDCl_3$) δ 8.53 (d, *J* = 5.2 Hz, 1H), 7.63-7.58 (m, 2H), 7.49-7.38 (m, 3H), 7.37-7.34 (m, 1H), 7.31 (dd, *J* = 5.2, 1.2 Hz, 1H), 3.61 (t, *J* = 6.4 Hz, 2H), 3.50 (br, 1H), 3.05-2.94 (m, 1H), 1.92-1.81 (m, 1H), 1.76-1.64 (m, 1H), 1.63-1.53 (m, 1H), 1.53-1.43 (m, 1H), 1.34 (d, *J* = 7.2 Hz, 3H); ^{13}C NMR (100 MHz, $CDCl_3$) δ 166.2, 148.8, 148.6, 138.0, 128.6, 128.5, 126.6, 119.1, 118.9, 62.0, 41.1, 32.8, 30.3, 20.5. FT-IR: ν (cm^{-1}) 3300, 3061, 2959, 2931, 2866, 2360, 2342, 1718. HRMS [ESI] calcd for $C_{16}H_{20}NO$ [M+H]$^+$ 242.1539, found 242.1537.

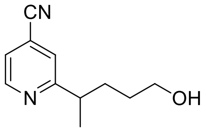

3m: yellow oil. ^{1}H NMR (400 MHz, $CDCl_3$) δ 8.69 (d, *J* = 5.2 Hz, 1H), 7.38-7.36 (m, 1H), 7.34 (dd, *J* = 4.8, 1.2 Hz, 1H), 3.60 (t, *J* = 6.4 Hz, 2H), 3.03-2.93 (m, 1H), 1.96 (br, 1H), 1.86-1.76 (m, 1H), 1.73-1.64 (m, 1H), 1.60-1.48 (m, 1H), 1.46-1.35 (m, 1H), 1.30 (d, *J* = 7.2 Hz, 3H); ^{13}C NMR (100 MHz, $CDCl_3$) δ 167.9, 150.1, 123.4, 122.6, 120.7, 116.7, 62.6, 41.6, 32.9, 30.5, 20.6. FT-IR: ν (cm^{-1}) 3357, 2934, 2871, 2238, 1595, 1550, 1459, 1398, 1291. HRMS [ESI] calcd for $C_{11}H_{15}N_2O$ [M+H]$^+$ 191.1179, found 191.1178.

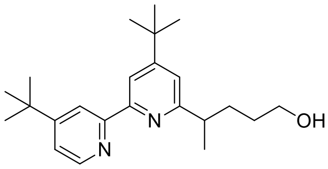

3n: yellow oil. ^{1}H NMR (400 MHz, $CDCl_3$) δ 8.57 (d, *J* = 5.2 Hz, 1H), 8.45 (d, *J* = 1.2 Hz, 1H), 8.19 (d, *J* = 1.6 Hz, 1H), 7.28 (d, *J* = 2.0 Hz, 1H), 7.13 (d, *J* = 1.6 Hz, 1H), 3.68-3.57 (m, 3H), 3.07-2.96 (m, 1H), 1.98-1.87 (m, 1H), 1.78-1.68 (m, 1H), 1.68-1.56 (m, 1H), 1.55-1.47 (m, 1H), 1.37 (m, 21H); ^{13}C NMR (100 MHz, $CDCl_3$) δ 165.2, 161.3, 160.7, 156.9, 155.5, 148.9, 120.5, 118.5, 118.4, 115.8, 63.0, 41.6, 35.0, 34.9, 33.5, 30.7, 30.6, 21.2. FT-IR: ν (cm^{-1}) 2963, 2934, 2869, 2360, 2342, 1589, 1546, 1478. HRMS [ESI] calcd for $C_{23}H_{35}N_2O$ [M+H]$^+$ 355.2744, found 355.2739.

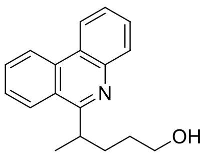

3o: yellow oil. ^{1}H NMR (400 MHz, $CDCl_3$) δ 8.64 (d, *J* = 8.0 Hz, 1H), 8.53 (d, *J* = 8.0 Hz, 1H), 8.31 (d, *J* = 8.0 Hz, 1H), 8.13 (d, *J* = 8.0 Hz, 1H), 7.85-7.78 (m, 1H), 7.74-7.65 (m, 2H), 7.64-7.57 (m, 1H), 3.92-3.81 (m, 1H), 3.66-3.48 (m, 2H), 2.55 (br, 1H), 2.43-2.32 (m, 1H), 1.92-1.80 (m, 1H), 1.80-1.67 (m, 1H), 1.62-1.51 (m, 1H), 1.46 (d, *J* = 6.4 Hz, 3H); ^{13}C NMR (100 MHz, $CDCl_3$) δ 164.6, 143.0, 132.6, 129.7, 129.1, 128.1, 126.8, 125.9, 125.2, 124.6, 123.0, 122.2, 121.4, 62.1, 36.2, 30.6, 30.5, 20.8. FT-IR: ν (cm^{-1}) 3073, 2930, 2868, 1611, 1581, 1526, 1487, 1456. HRMS [ESI] calcd for $C_{18}H_{20}NO$ [M+H]$^+$ 266.1539, found 266.1545.

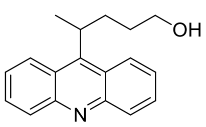

3p: yellow solid, m.p. 156-157 ºC. ^{1}H NMR (400 MHz, $CDCl_3$) δ 8.53-8.31 (m, 2H), 8.24 (d, *J* = 8.4 Hz, 2H), 7.77-7.67 (m, 2H), 7.57-7.44 (m, 2H), 4.41-4.27 (m, 1H), 3.57 (t, *J* = 6.4 Hz, 2H), 2.37-2.18 (m, 2H), 1.73 (t, *J* = 7.2 Hz, 3H), 1.70-1.58 (m, 1H), 1.39-1.26 (m, 1H); ^{13}C NMR (100 MHz, $CDCl_3$) δ 150.8, 148.2, 147.9, 130.0, 129.7, 129.2, 125.6, 125.4, 125.2, 124.9, 124.1, 124.0, 123.2, 62.0, 33.6, 33.1, 31.5, 20.8. FT-IR: ν (cm^{-1}) 3213, 3063, 3050, 2961, 2931, 2860, 2830, 1720. HRMS [ESI] calcd for $C_{18}H_{20}NO$ [M+H]$^+$ 266.1539, found 266.1541.

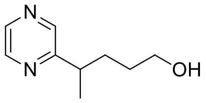

3q: yellow oil. ^{1}H NMR (400 MHz, $CDCl_3$) δ 8.48 (dd, *J* = 2.4, 1.6 Hz, 1H), 8.44 (d, *J* = 1.2 Hz, 1H), 8.38 (d, *J* = 2.4 Hz, 1H), 3.60 (t, *J* = 6.4 Hz, 2H),

3.00-2.90 (m, 1H), 1.91 (br, 1H), 1.87-1.78 (m, 1H), 1.76-1.66 (m, 1H), 1.61-1.50 (m, 1H), 1.47-1.37 (m, 1H), 1.31 (d, *J* = 6.8 Hz, 3H); ^{13}C NMR (100 MHz, $CDCl_3$) δ 161.5, 144.0, 143.9, 142.3, 62.6, 39.2, 32.7, 30.6, 20.6. FT-IR: ν (cm^{-1}) 3356, 3055, 2961, 2932, 2870, 1722, 1671, 1577. HRMS [ESI] calcd for $C_9H_{15}N_2O$ $[M+H]^+$ 167.1179, found 167.1178.

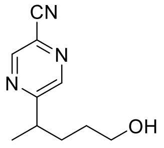

3r: yellow oil. ^{1}H NMR (400 MHz, $CDCl_3$) δ 8.81 (d, *J* = 1.2 Hz, 1H), 8.55 (d, *J* = 1.6 Hz, 1H), 3.60 (t, *J* = 6.4 Hz, 2H), 3.11-3.00 (m, 1H), 2.05 (br, 1H), 1.90-1.79 (m, 1H), 1.78-1.68 (m, 1H), 1.60-1.48 (m, 1H), 1.45-1.36 (m, 1H), 1.33 (t, *J* = 7.2 Hz, 3H); ^{13}C NMR (100 MHz, $CDCl_3$) δ 164.8, 147.0, 144.2, 127.6, 115.2, 61.9, 39.2, 32.1, 29.9, 19.7. FT-IR: ν (cm^{-1}) 3387, 2933, 2873, 2360, 2237, 1718, 1567, 1567. HRMS [ESI] calcd for $C_{10}H_{14}N_3O$ $[M+H]^+$ 192.1131, found 192.1134.

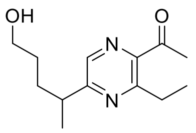

3s: yellow oil. ^{1}H NMR (400 MHz, $CDCl_3$) δ 8.28 (s, 1H), 3.64-3.56 (m, 2H), 3.11 (q, *J* = 7.6 Hz, 2H), 3.04-2.94 (m, 1H), 2.66 (s, 3H), 2.10 (br, 1H), 1.90-1.80 (m, 1H), 1.76-1.66 (m, 1H), 1.61-1.49 (m, 1H), 1.47-1.37 (m, 1H), 1.31 (d, *J* = 6.8 Hz, 3H), 1.24 (t, *J* = 7.2 Hz, 3H); ^{13}C NMR (100 MHz, $CDCl_3$) δ 200.9, 162.6, 157.5, 143.9, 138.7, 62.1, 38.9, 32.1, 30.0, 28.4, 27.6, 19.8, 12.7. FT-IR: ν (cm^{-1}) 2967, 2935, 2874, 2361, 2343, 1698, 1552, 1533. HRMS [ESI] calcd for $C_{13}H_{20}N_2O_2Na$ $[M+Na]^+$ 259.1417, found 259.1411.

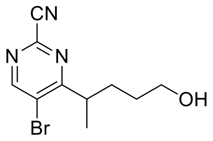

3t: yellow oil. ^{1}H NMR (400 MHz, $CDCl_3$) δ 8.80 (s, 1H), 3.61 (t, *J* = 6.8 Hz, 2H), 3.46-3.36 (m, 1H), 2.04-1.82 (m, 2H), 1.73-1.63 (m, 1H), 1.62-1.50 (m, 1H), 1.47-1.35 (m, 1H), 1.26 (d, *J* = 6.8 Hz, 3H); ^{13}C NMR (100 MHz, $CDCl_3$) δ 174.2, 159.0, 142.6, 124.0, 114.9, 62.0, 38.7, 30.8, 29.8, 18.5. FT-IR: ν (cm^{-1}) 3356, 2930, 2861, 2360, 2342, 1722, 1547, 1512. HRMS [ESI] calcd for $C_{10}H_{12}BrN_3ONa$ $[M+Na]^+$ 292.0056, found 292.0056.

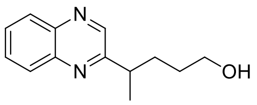

3u: yellow oil. ^{1}H NMR (400 MHz, $CDCl_3$) δ 8.76 (s, 1H), 8.09-8.03 (m, 2H), 7.77-7.68 (m, 2H), 3.68-3.58 (m, 2H), 3.24-3.14 (m, 1H), 2.18 (br, 1H), 2.05-1.95 (m, 1H), 1.88-1.78 (m, 1H), 1.70-1.58 (m, 1H), 1.54-1.46 (m, 1H), 1.43 (d, *J* = 6.8 Hz, 3H); ^{13}C NMR (100 MHz, $CDCl_3$) δ 161.0, 145.0, 141.9, 141.4, 130.0, 129.1, 129.1, 128.9, 62.6, 40.1, 32.5, 30.7, 20.5. FT-IR: ν (cm^{-1}) 3364, 2933, 2870, 1717, 1559, 1493, 1456, 1369, 1276. HRMS [ESI] calcd for $C_{13}H_{17}N_2O$ $[M+H]^+$ 217.1335, found 217.1330.

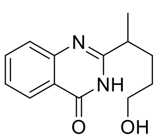

3v: white solid, m.p. 116-117 °C. ^{1}H NMR (400 MHz, $CDCl_3$) δ 11.9 (br, 1H), 8.28 (d, *J* = 8.0 Hz, 1H), 7.79-7.69 (m, 2H), 7.46 (dd, *J* = 7.2, 7.2 Hz, 1H), 3.71 (t, *J* = 6.0 Hz, 2H), 3.02-2.94 (m, 1H), 2.13-2.02 (m, 1H), 1.82-1.68 (m, 2H), 1.68-1.58 (m, 1H), 1.43 (d, *J* = 6.8 Hz, 3H); ^{13}C NMR (100 MHz, $CDCl_3$) δ 163.9, 159.8, 148.8, 134.4, 126.8, 126.1, 125.8, 120.1, 61.5, 39.0, 30.9, 29.5, 17.9. FT-IR: ν (cm^{-1}) 3172, 3126, 3034, 2972, 2924, 2870, 2360, 1677. HRMS [ESI] calcd for $C_{13}H_{17}N_2O_2$ $[M+H]^+$ 233.1285, found 233.1284.

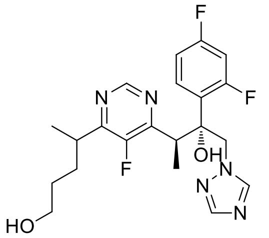

3w (*d.r.* = 1.2:1): yellow oil. ^{1}H NMR (400 MHz, $CDCl_3$) δ 8.81 (dd, *J* = 4.0, 1.6 Hz, 1H, two isomers), 8.03 (d, *J* = 7.2 Hz, 1H, two isomers), 7.66-7.57 (m, 1H, two isomers), 7.54 (d, *J* = 8.8 Hz, 1H, two isomers), 6.87-6.79 (m, 2H, two isomers), 4.70 (m, 1H, two isomers), 4.32 (m, 1H, two isomers), 4.11 (q, *J* = 7.2 Hz, 1H, two isomers), 3.65 (d, *J* = 6.8 Hz, 1H, two isomers), 3.62 (d, *J* = 6.8 Hz, 1H, two isomers), 3.37-3.26 (m, 1H, two isomers), 2.14 (br, 2H, two isomers), 1.96-1.85 (m, 1H, two isomers), 1.79-1.67 (m, 1H, two isomers), 1.66-1.55 (m, 1H, two isomers), 1.52 -1.38 (m, 1H, two isomers), 1.36-1.29 (m, 3H, two isomers), 1.07 (d, *J* = 7.2 Hz, 3H, two isomers); ^{13}C NMR (100 MHz, $CDCl_3$) δ 163.5 (d, J_{C-F} = 12.3 Hz, one isomer), 162.5 & 162.4 (two isomers), 161.0 (d, J_{C-F} = 12.2 Hz, one isomer), 159.3 (d, J_{C-F} = 12.1 Hz, one isomer), 157.5 (d, J_{C-F} = 6.6 Hz, one isomer) & 157.4 (d, J_{C-F} = 6.6 Hz, one isomer), 156.8 (d, J_{C-F} = 11.7 Hz, one isomer), 154.4 (d, J_{C-F} = 5.3 Hz, one isomer), 152.3 (d, J_{C-F} = 2.8 Hz, one isomer) & 152.3 (d, J_{C-F} = 2.9 Hz, one isomer), 151.8 (d, J_{C-F} = 5.2 Hz, one isomer), 150.0 & 149.8 (two isomers), 143.4 (two isomers), 130.1 (d, J_{C-F} = 5.9 Hz, one isomers) & 130.1 (d, J_{C-F} = 5.9 Hz, one isomers), 123.2 (dd, J_{C-F} = 8.5, 3.8 Hz, one isomer) & 123.1 (dd, J_{C-F} = 8.2, 3.8 Hz, one isomer), 111.2 (d, J_{C-F} = 2.6 Hz, one isomer) & 110.9 (d, J_{C-F} = 3.2 Hz, one isomer), 103.7 (d, J_{C-F} = 27.2 Hz, one isomer) & 103.5 (d, J_{C-F} = 27.2 Hz, one isomer), 76.9 (two isomers), 61.7 & 61.7 (two isomers), 57.1 & 57.0 (two isomers), 35.9 (d, J_{C-F} = 4.3 Hz, one isomer), 35.9 (d, J_{C-F} = 4.3 Hz, one isomer), 33.9 & 33.7 (two isomers), 30.9 & 30.7 (two isomers), 30.0 & 29.9 (two isomers), 18.6 & 18.5 (two isomers), 15.8 & 15.7 (two isomers); ^{19}F NMR (376 MHz, $CDCl_3$) δ -109.00--109.02 (m, two isomers), -110.3--110.5 (m, two isomers), -139.09 & -139.22 (two isomers). FT-IR: ν (cm^{-1}) 3062, 2949, 2868, 1601, 1558, 1508, 1474, 1447. HRMS [ESI] calcd for $C_{21}H_{24}F_3N_5O_2Na$ $[M+Na]^+$ 458.1770, found 458.1773.

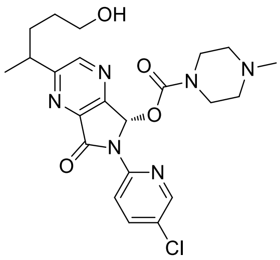

3x (*d.r.* = 1:1): yellow oil. ^{1}H NMR (400 MHz, $CDCl_3$) δ 8.71 (s, 1H, two isomers), 8.46 (d, *J* = 8.8 Hz, 1H, two isomers), 8.36 (d, *J* = 2.4 Hz, 1H, two isomers), 7.92 (s, 1H, two isomers), 7.76 (dd, *J* = 8.8, 2.4 Hz, 1H, two isomers), 3.74-3.52 (m, 4H, two isomers), 3.38-3.24 (m, 2H, two isomers), 3.21-3.12 (m, 1H, two isomers), 2.61-2.20 (m, 7H, two isomers), 2.17-2.02 (m, 1H, two isomers), 2.00-1.86 (m, 1H, two isomers), 1.83-1.71 (m, 1H, two isomers), 1.64-1.53 (m, 1H, two isomers), 1.49-1.36 (m, 4H, two isomers); ^{13}C NMR (100 MHz, $CDCl_3$) δ 166.5 & 166.5 (two isomers), 162.9 (two isomers), 154.4 (two isomers), 153.0 & 152.9 (two isomers), 147.5 & 147.5 (two isomers), 146.6 & 146.4 (two isomers), 146.2 (two isomers), 140.8 (two isomers), 137.6 (two isomers), 127.6 (two isomers), 115.6 (two isomers), 78.7 & 78.7 (two isomers), 61.9 & 61.8 (two isomers), 53.9 & 53.8 (two isomers), 45.3 & 45.2 (two isomers), 43.3 & 43.1 (two isomers), 39.5 (two isomers), 32.3 & 32.0 (two isomers) , 30.0 (two isomers), 20.2 & 19.9 (two isomers). FT-IR: ν (cm^{-1}) 3032, 2948, 2878, 1601, 1561, 1508, 1424, 1358. HRMS [ESI] calcd for $C_{22}H_{28}ClN_6O_4$ $[M+H]^+$ 475.1855, found 475.1859.

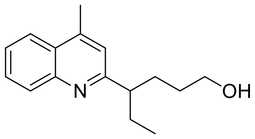

3y: yellow oil. ^{1}H NMR (400 MHz, $CDCl_3$) δ 8.05-8.01 (m, 1H), 7.90 (dd, *J* = 8.0, 0.8 Hz, 1H), 7.61 (ddd, *J* = 8.4, 6.8, 1.2 Hz, 1H), 7.46 (ddd, *J* = 8.4, 6.8, 0.8 Hz, 1H), 7.11-7.07 (m, 1H), 3.65-3.51 (m, 3H), 2.88-2.79 (m, 1H), 2.63 (d, *J* = 0.8 Hz, 3H), 1.85-1.70 (m, 4H), 1.58-1.49 (m, 1H),

1.44-1.34 (m, 1H), 0.79 (t, *J* = 7.2 Hz, 3H); ^{13}C NMR (100 MHz, $CDCl_3$) δ 164.9, 146.8, 144.1, 128.7, 128.6, 126.6, 125.1, 123.1, 120.2, 61.9, 49.3, 31.0, 30.2, 28.1, 18.4, 11.7. FT-IR: ν (cm^{-1}) 2959, 2932, 2872, 2360, 2341, 1737, 1603, 1509. HRMS [ESI] calcd for $C_{16}H_{22}NO$ $[M+H]^+$ 244.1696, found 244.1698.

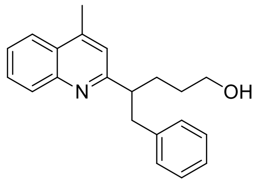

3z: yellow oil. ^{1}H NMR (400 MHz, $CDCl_3$) δ 8.06 (d, *J* = 8.4 Hz, 1H), 7.96-7.92 (m, 1H), 7.67 (ddd, *J* = 8.0, 6.8, 1.2 Hz, 1H), 7.50 (ddd, *J* = 8.0, 7.2, 1.2 Hz, 1H), 7.23-7.17 (m, 2H), 7.16-7.10 (m, 3H), 7.04 (s, 1H), 3.58-3.46 (m, 2H), 3.31-3.22 (m, 1H), 3.20-3.13 (m, 1H), 3.01-2.94 (m, 1H), 2.64 (s, 3H), 2.69-2.58 (m, 1H), 2.02-1.92 (m, 1H), 1.86-1.78 (m, 1H), 1.58-1.47 (m, 1H), 1.45-1.34 (m, 1H); ^{13}C NMR (100 MHz, $CDCl_3$) δ 164.3, 147.5, 144.4, 140.3, 129.3, 129.2, 129.1, 128.2, 127.1, 125.9, 125.6, 123.6, 121.6, 62.5, 49.7, 42.2, 30.6, 30.5, 18.8. FT-IR: ν (cm^{-1}) 3084, 3061, 3027, 2934, 2858, 1602, 1561, 1509. HRMS [ESI] calcd for $C_{21}H_{24}NO$ $[M+H]^+$ 306.1852, found 306.1856.

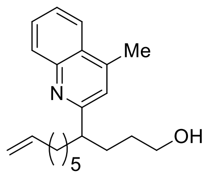

3aa: yellow oil. ^{1}H NMR (400 MHz, $CDCl_3$) δ 8.04 (d, *J* = 8.4 Hz, 1H), 7.92 (dd, *J* = 8.4, 0.8 Hz, 1H), 7.63 (ddd, *J* = 8.4, 7.2, 1.6 Hz, 1H), 7.48 (ddd, *J* = 8.0, 6.8, 1.2 Hz, 1H), 7.10 (s, 1H), 5.79-5.67 (m, 1H), 4.95-4.83 (m, 2H), 3.62-3.52 (m, 2H), 3.42 (br, 1H), 2.98-2.88 (m, 1H), 2.66 (s, 3H), 1.99-1.90 (m, 2H), 1.86-1.78 (m, 2H), 1.78-1.66 (m, 2H), 1.60-1.49 (m, 1H), 1.45-1.34 (m, 1H), 1.32-1.20 (m, 6H); ^{13}C NMR (100 MHz, $CDCl_3$) δ 165.0, 146.8, 144.1, 138.6, 128.7, 128.6, 126.6, 125.1, 123.1, 120.1, 113.7, 62.0, 47.6, 35.3, 33.2, 31.3, 30.2, 28.8, 28.2, 27.0, 18.4. FT-IR: ν (cm^{-1}) 2927, 2855, 2361, 2342, 1639, 1603, 1561, 1509. HRMS [ESI] calcd for $C_{21}H_{30}NO$ $[M+H]^+$ 312.2322, found 312.2336.

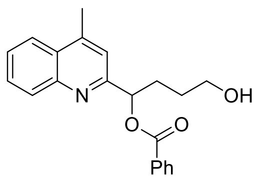

3ab: yellow solid, m.p. 110-111 °C. ^{1}H NMR (400 MHz, $CDCl_3$) δ 8.14 (d, *J* = 7.2 Hz, 2H), 8.08 (d, *J* = 8.8 Hz, 1H), 7.94 (d, *J* = 8.4 Hz, 1H), 7.68 (dd, *J* = 7.6, 7.6 Hz, 1H), 7.60-7.50 (m, 2H), 7.46 (dd, *J* = 7.6, 7.6 Hz, 2H), 7.36 (s, 1H), 6.23 (t, *J* = 6.8 Hz, 1H), 3.74 (t, *J* = 6.0 Hz, 2H), 3.15 (br, 1H), 2.67 (s, 3H), 2.31-2.24 (m, 2H), 1.88-1.71 (m, 2H); ^{13}C NMR (100 MHz, $CDCl_3$) δ 165.5, 159.2, 146.6, 145.2, 132.7, 129.6, 129.3, 129.1, 129.0, 128.0, 127.1, 125.9, 123.2, 118.4, 76.8, 61.5, 31.0, 28.1, 18.6. FT-IR: ν (cm^{-1}) 3245, 2952, 2867, 1717, 1598, 1565, 1489, 1449, 1348. HRMS [ESI] calcd for $C_{21}H_{22}NO_3$ $[M+H]^+$ 336.1594, found 336.1582.

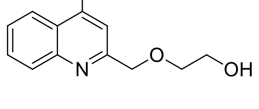

3ac: yellow oil. ^{1}H NMR (400 MHz, $CDCl_3$) δ 8.05 (d, *J* = 8.4 Hz, 1H), 7.95-7.92 (m, 1H), 7.66 (ddd, *J* = 8.0, 6.8, 1.2 Hz, 1H), 7.54-7.49 (ddd, *J* = 8.0, 4.8, 1.2 Hz, 1H), 7.26 (s, 1H), 4.81 (s, 2H), 4.39 (br, 1H), 3.84-3.80 (m, 2H), 3.78-3.74 (m, 2H), 2.65 (s, 3H); ^{13}C NMR (100 MHz, $CDCl_3$) δ 158.4, 147.1, 145.3, 129.5, 129.2, 127.5, 126.2, 123.7, 119.9, 73.8, 73.1, 61.8, 18.8. FT-IR: ν (cm^{-1}) 3259, 2920, 2862, 1602, 1566, 1509, 1447, 1413, 1263. HRMS [ESI] calcd for $C_{13}H_{16}NO_2$ $[M+H]^+$ 218.1176, found 218.1178.

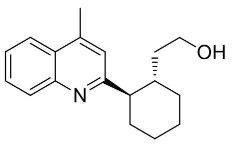

3ad (*d.r.* > 19:1): yellow oil. ^{1}H NMR (400 MHz, $CDCl_3$) δ 8.00 (d, *J* = 8.4 Hz, 1H), 7.90 (dd, *J* = 8.0, 0.8 Hz, 1H), 7.63 (ddd, *J* = 8.4, 6.8, 1.2 Hz, 1H), 7.47 (ddd, *J* = 8.4, 7.2, 1.2 Hz, 1H), 7.12 (s, 1H), 3.53-3.45 (m, 3H), 2.70-2.62 (m, 4H), 2.12-2.02 (m, 1H), 1.98-1.88 (m, 2H), 1.84-1.78 (m, 2H), 1.56-1.44 (m, 1H), 1.44-1.35 (m, 3H), 1.34-1.12 (m, 2H); ^{13}C NMR (100 MHz, $CDCl_3$) δ 165.1, 146.7, 144.3, 128.7, 128.6, 126.5, 125.1, 123.1, 120.9, 59.9, 52.1, 37.2, 37.0, 34.3, 32.3, 26.0, 25.8, 18.4. FT-IR: ν (cm^{-1}) 2923, 2852, 2360, 2341, 1737, 1603, 1561, 1508. HRMS [ESI] calcd for $C_{18}H_{24}NO$ $[M+H]^+$ 270.1852, found 270.1854.

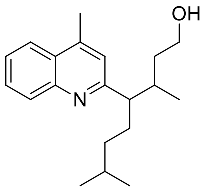

3ae (*d.r.* = 2:1): yellow oil. ^{1}H NMR (400 MHz, $CDCl_3$) δ 8.05 (d, *J* = 8.4 Hz, 3H, two isomers), 7.97-7.92 (m, 3H, two isomers), 7.69-7.62 (m, 3H, two isomers), 7.53-7.47 (m, 3H, two isomers), 7.11 (s, 3H, two isomers), 3.86-3.79 (m, 1H, one isomer), 3.77-3.71 (m, 1H, one isomer), 3.71-3.64 (m, 2H, one isomer), 3.61-3.54 (m, 2H, one isomer), 3.52-3.10 (br, 3H, two isomers), 3.08-3.02 (m, 1H, one isomer), 2.86-2.78 (m, 2H, one isomer), 2.70-2.66 (m, 9H , two isomers), 2.16-2.00 (m, 3H, two isomers), 1.96-1.64 (m, 8H, two isomers), 1.56-1.41 (m, 7H, two isomers), 1.40-1.30 (m, 3H , two isomers), 1.09-0.97 (m, 9H, two isomers), 0.84-0.77 (m, 21H, two isomers); ^{13}C NMR (100 MHz, $CDCl_3$) δ 164.4 & 163.1 (two isomers), 146.4 & 146.2 (two isomers), 144.1 & 143.7 (two isomers), 128.7 & 128.6 (two isomers), 128.6 & 128.5 (two isomers), 126.5 & 126.4 (two isomers), 125.2 (two isomers), 123.1 & 123.1 (two isomers), 121.2 & 121.1 (two isomers), 61.0 & 59.9 (two isomers), 52.9 & 51.6 (two isomers), 37.3 & 36.9 (two isomers), 36.7 & 36.2 (two isomers), 35.7 & 34.1 (two isomers), 29.2 (two isomers), 28.5 & 27.7 (two isomers), 22.3 & 22.2 (two isomers), 21.9 & 21.8 (two isomers), 18.5 & 18.5 (two isomers), 17.0 & 16.6 (two isomers). FT-IR: ν (cm^{-1}) 2955, 2929, 2870, 1603, 1560, 1509, 1449, 1382. HRMS [ESI] calcd for $C_{20}H_{29}NONa$ $[M+Na]^+$ 322.2141, found 322.2132.

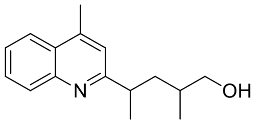

3af (*d.r.* = 1:1): yellow oil. ^{1}H NMR (400 MHz, $CDCl_3$) δ 8.04 (d, *J* = 8.4 Hz, 2H, two isomers), 7.94-7.89 (m, 2H, two isomers), 7.67-7.61 (m, 2H, two isomers), 7.51-7.45 (m, 2H, two isomers), 7.17-7.14 (m, 2H, two isomers), 4.00 (br, 2H, two isomers), 3.52-3.42 (m, 2H, two isomers), 3.41-3.38 (m, 2H, two isomers), 3.33-3.23 (m, 1H, one isomer), 3.23-3.15 (m, 1H, one isomer), 2.66 (d, *J* = 0.8 Hz, 3H, one isomer), 2.65 (d, *J* = 0.8 Hz, 3H, one isomer), 2.22-2.13 (m, 1H, one isomer), 1.78-1.71 (m, 2H, two isomers), 1.71-1.64 (m, 1H, one isomer), 1.57-1.48 (m, 1H, one isomer), 1.44-1.37 (m, 1H, one isomer), 1.34 (d, *J* = 3.6 Hz, 3H, one isomer), 1.32 (d, *J* = 3.6 Hz, 3H, one isomer), 0.96 (d, *J* = 6.8 Hz, 3H, one isomer), 1.87 (d, *J* = 6.8 Hz, 3H, one isomer); ^{13}C NMR (100 MHz, $CDCl_3$) δ 166.5 & 166.1 (two isomers), 147.0 & 146.9 (two isomers), 145.1 & 145.0 (two isomers), 129.3 & 129.2 (two isomers), 129.0 & 129.0 (two isomers), 127.0 & 127.0 (two isomers), 125.7 (two isomers), 123.6 (two isomers), 120.8 & 120.1 (two isomers), 68.1 & 67.5 (two isomers), 41.1 & 40.1 (two isomers), 39.7 & 39.5 (two isomers), 34.3 & 33.6 (two isomers), 22.4 & 21.0 (two isomers), 18.9 & 18.9 (two isomers), 17.6 & 17.4 (two isomers). FT-IR: ν (cm^{-1}) 3063, 2959, 2925, 2870, 2360, 2342, 1732, 1509. HRMS [ESI] calcd for $C_{16}H_{22}NO$ $[M+H]^+$ 244.1696, found 244.1698.

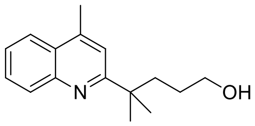

3ag: yellow oil. ^{1}H NMR (400 MHz, $CDCl_3$) δ 8.05 (d, *J* = 8.4 Hz, 1H), 7.92 (dd, *J* = 8.0, 0.8 Hz, 1H), 7.64 (ddd, *J* = 8.0, 6.8, 1.2 Hz, 1H), 7.48 (ddd, *J* = 8.0, 6.8, 1.2 Hz, 1H), 7.31 (s, 1H), 3.53 (t, *J* = 6.0 Hz, 2H), 3.31 (br, 1H), 2.67 (s, 3H), 1.98-1.92 (m, 2H), 1.51-1.45 (m, 2H), 1.42 (s, 6H); ^{13}C NMR (100 MHz, $CDCl_3$) δ 167.3, 146.5, 143.6, 129.0, 128.5, 126.1, 125.1, 123.0, 119.0, 62.4, 40.2, 37.4, 27.9, 27.7, 18.5. FT-IR: ν (cm^{-1}) 3062, 2949, 2868, 1601, 1558, 1508, 1474, 1447. HRMS [ESI] calcd for $C_{16}H_{22}NO$ $[M+H]^+$ 244.1696, found 244.1695.

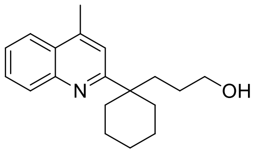

3ah: yellow oil. ^{1}H NMR (400 MHz, $CDCl_3$) δ 8.00 (d, *J* = 8.4 Hz, 1H), 7.91 (d, *J* = 8.0 Hz, 1H), 7.64 (ddd, *J* = 8.0, 7.2, 1.2 Hz, 1H), 7.48 (ddd, *J* = 8.0, 7.2, 1.2 Hz, 1H), 7.13 (s, 1H), 3.56-3.45 (m, 2H), 2.71-2.61 (m, 1H), 2.65 (s, 3H), 2.12-2.02 (m, 1H), 1.99-1.89 (m, 2H), 1.85-1.78 (m, 2H), 1.67 (br, 1H), 1.57-1.48 (m, 1H), 1.53-1.48 (m, 4H), 1.33-1.27 (m, 1H), 1.26-1.12 (m, 2H); ^{13}C NMR (100 MHz, $CDCl_3$) δ 165.1, 146.7, 144.3, 128.7, 128.6, 126.5, 125.1, 123.1, 120.9, 59.9, 52.1, 37.2, 37.0, 34.3, 32.9, 32.3, 26.1, 25.8, 18.4. FT-IR: ν (cm^{-1}) 3318, 2923, 2852, 2361, 2342, 2233, 1604, 1508. HRMS [ESI] calcd for $C_{19}H_{25}NONa$ $[M+Na]^+$ 306.1828, found 306.1842.

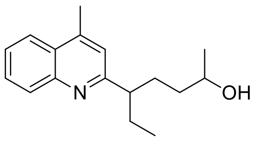

3ai: (*d.r.* = 1:1): yellow oil. ^{1}H NMR (400 MHz, $CDCl_3$) δ 8.05 (d, *J* = 8.4 Hz, 2H, two isomers), 7.96-7.90 (m, 2H, two isomers), 7.68-7.62 (m, 2H, two isomers), 7.52-7.46 (m, 2H, two isomers), 7.13-7.09 (m, 2H, two isomers), 3.83-3.74 (m, 1H, one isomer), 3.73-3.64 (m, 1H, one isomer), 2.89-2.81 (m, 2H, two isomers), 2.69-2.64 (m, 8H, two isomers), 1.96-1.73 (m, 8H, two isomers), 1.50-1.40 (m, 2H, two isomers), 1.36-1.18 (m, 2H, two isomers), 1.10 (d, *J* = 6.0 Hz, 3H, one isomer), 1.09 (d, *J* = 6.0 Hz, 3H, one isomer), 0.83 (t, *J* = 7.2 Hz, 3H, one isomer), 0.82 (t, *J* = 7.2 Hz, 3H, one isomer); ^{13}C NMR (100 MHz, $CDCl_3$) δ 165.4 & 165.2 (two isomers), 147.4 & 147.3 (two isomers), 144.5 & 144.3 (two isomers), 129.4 & 129.3 (two isomers), 129.1 & 129.0 (two isomers), 127.0 (two isomers), 125.6 & 125.5 (two isomers), 123.6 (two isomers), 120.9 & 120.8 (two isomers), 68.0 & 67.7 (two isomers), 50.0 & 49.8 (two isomers), 37.2 & 37.0 (two isomers), 31.0 & 30.9 (two isomers), 28.8 & 28.8 (two isomers), 23.5 & 23.4 (two isomers), 18.9 (two isomers), 12.1 & 12.1 (two isomers). FT-IR: ν (cm^{-1}) 3340, 2962, 2929, 2873, 2361, 2232, 1684, 1509. HRMS [ESI] calcd for $C_{17}H_{24}NO$ $[M+H]^+$ 258.1852, found 258.1856.

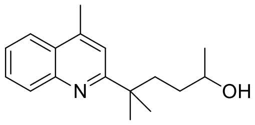

3aj: yellow oil. ^{1}H NMR (400 MHz, $CDCl_3$) δ 8.06 (d, *J* = 8.4 Hz, 1H), 7.95-7.91 (m, 1H), 7.65 (ddd, *J* = 8.4, 7.2, 1.2 Hz, 1H), 7.52-7.47 (ddd, *J* = 8.0, 6.8, 0.8 Hz, 1H), 7.33-7.30 (m, 1H), 3.78-3.69 (m, 1H), 3.58 (br, 1H), 2.68 (d, *J* = 0.8 Hz, 3H), 2.11-2.02 (m, 1H), 2.01-1.92 (m, 1H), 1.50-1.44 (m, 1H), 1.41 (s, 3H), 1.40 (s, 3H), 1.38-1.30 (m, 1H), 1.12 (d, *J* = 6.0 Hz, 3H); ^{13}C NMR (100 MHz, $CDCl_3$) δ 167.2, 146.3, 143.8, 128.9, 128.6, 126.0, 125.2, 122.9, 119.2, 67.5, 40.3, 36.6, 34.1, 28.3, 28.0, 22.9, 18.5. FT-IR: ν (cm^{-1}) 3356, 3063, 2964, 2928, 2867, 2361, 2341, 1508. HRMS [ESI] calcd for $C_{17}H_{24}NO$ $[M+H]^+$ 258.1852, found 258.1845.

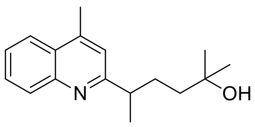

3ak: yellow oil. ^{1}H NMR (400 MHz, $CDCl_3$) δ 8.07-8.03 (m, 1H), 7.93 (dd, *J* = 8.4, 0.8 Hz, 1H), 7.65 (ddd, *J* = 8.0, 6.8, 1.2 Hz, 1H), 7.48 (ddd, *J* = 8.4, 7.2, 1.6 Hz, 1H), 7.14-7.13 (m, 1H), 3.10-3.00 (m, 1H), 2.67 (d, *J*

= 0.8 Hz, 3H), 2.32 (br, 1H), 2.00-1.89 (m, 1H), 1.82-1.72 (m, 1H), 1.62-1.49 (m, 1H), 1.36 (d, *J* = 7.2 Hz, 3H), 1.33-1.28 (m, 1H), 1.18 (s, 3H), 1.17 (s, 3H); ^{13}C NMR (100 MHz, $CDCl_3$) δ 166.2, 147.3, 144.6, 129.4, 129.1, 127.0, 125.5, 123.6, 120.4, 70.7, 42.7, 41.4, 31.1, 29.4, 29.3, 21.2, 18.9. FT-IR: ν (cm^{-1}) 3368, 2966, 2870, 1670, 1604, 1561, 1449, 1344, 1296. HRMS [ESI] calcd for $C_{17}H_{24}NO$ [M+H]$^+$ 258.1852, found 258.1845.

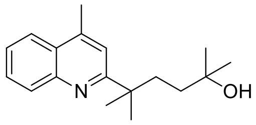

3al: yellow oil. ^{1}H NMR (400 MHz, $CDCl_3$) δ 8.07 (d, *J* = 8.4 Hz, 1H), 7.93 (dd, *J* = 8.4, 0.8 Hz, 1H), 7.65 (ddd, *J* = 8.4, 6.8, 1.2 Hz, 1H), 7.50 (ddd, *J* = 8.0, 6.8, 1.2 Hz, 1H), 7.32 (s, 1H), 3.74 (br, 1H), 2.68 (s, 3H), 2.05-2.00 (m, 2H), 1.45-1.42 (m, 2H), 1.41 (s, 6H), 1.19 (s, 6H); ^{13}C NMR (100 MHz, $CDCl_3$) δ 167.1, 146.4, 143.7, 128.9, 128.5, 126.0, 125.2, 122.9, 119.3, 70.1, 40.3, 38.1, 35.0, 28.9, 28.2, 18.5. FT-IR: ν (cm^{-1}) 3062, 2966, 2930, 2869, 2360, 2341, 2226, 1508. HRMS [ESI] calcd for $C_{18}H_{26}NO$ [M+H]$^+$ 272.2009, found 272.2011.

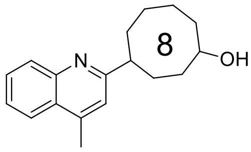

3am (*dr* = 1:1): yellow oil. ^{1}H NMR (400 MHz, $CDCl_3$) δ 8.03 (d, *J* = 8.4 Hz, 2H, two isomers), 7.94 (dd, *J* = 8.4, 0.8 Hz, 2H, two isomers), 7.66 (ddd, *J* = 8.0, 6.8, 1.2 Hz, 2H, two isomers), 7.49 (ddd, *J* = 8.0, 6.8, 1.2 Hz, 2H, two isomers), 7.13 (d, *J* = 0.4 Hz, 1H, one isomer), 7.10 (d, *J* = 0.8 Hz, 1H, one isomer), 4.06-3.96 (m, 2H, two isomers), 3.14-3.01 (m, 2H, two isomers), 2.67 (d, *J* = 0.8 Hz, 6H , two isomers), 2.15-2.00 (m, 6H, two isomers), 1.98-1.53 (m, 20H, two isomers); ^{13}C NMR (100 MHz, $CDCl_3$) δ 167.8 & 167.7 (two isomers), 144.6 & 144.5 (two isomers), 129.5 & 129.4 (two isomers), 129.0 & 129.0 (two isomers), 126.9 & 126.9 (two isomers), 125.4 (two isomers), 123.6 (two isomers), 120.7 (two isomers), 120.4 (two isomers), 72.3 & 71.4 (two isomers), 48.2 & 47.9 (two isomers), 34.8 & 34.5 (two isomers), 33.9 & 33.4 (two isomers), 31.9 & 31.6 (two isomers), 29.6 & 28.5 (two isomers), 26.7 & 26.6 (two isomers), 23.0 & 22.4 (two isomers), 18.9 & 18.8 (two isomers). FT-IR: ν (cm^{-1}) 3328, 2923, 2854, 2360, 2342, 2203, 1731, 1603. HRMS [ESI] calcd for $C_{18}H_{24}NO$ [M+H]$^+$ 270.1852, found 270.1850.

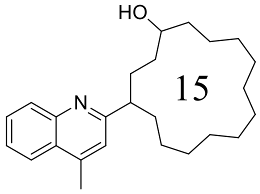

3an (*dr* = 1.8:1): yellow oil. ^{1}H NMR (400 MHz, $CDCl_3$) δ 8.10-8.04 (m, 2.8H, two isomers), 7.94 (d, *J* = 8.4 Hz, 2.8H, two isomers), 7.69-7.62 (m, 2.8H, two isomers), 7.53-7.46 (m, 2.8H, two isomers), 7.15 (s, 1.8H, one isomer), 7.12 (s, 1H, one isomer), 3.94-3.86 (m, 1.8H, one isomer), 3.76-3.67 (m, 1H, one isomer), 3.12-2.98 (m, 2.8H, two isomers), 2.69-2.66 (m, 8.4H, two isomers), 1.98-1.86 (m, 2.8H, two isomers), 1.86-1.71 (m, 8.4H, two isomers), 1.68-1.55 (m, 8.4H, two isomers), 1.52-1.32 (m, 53.2H, two isomers); ^{13}C NMR (100 MHz, $CDCl_3$) δ 165.5 (two isomers), 128.9 (two isomers), 128.7 (two isomers), 128.6 & 128.5 (two isomers), 126.5 (two isomers), 125.2 (two isomers), 125.0 (two isomers), 123.1 & 123.1 (two isomers), 120.3 & 120.1 (two isomers), 71.0 & 68.5 (two isomers), 46.4 & 44.2 (two isomers), 34.8 & 34.8 (two isomers), 33.6 & 33.4 (two isomers), 33.0 (two isomers), 32.4 (two isomers), 29.1 & 28.7 (two isomers), 26.7 & 26.6 (two isomers), 26.5 (two isomers), 26.4 & 26.3 (two isomers), 26.2 & 26.2 (two isomers) , 26.2 & 26.1 (two isomers), 25.3 & 25.2 (two isomers), 23.2 & 22.4 (two isomers), 18.5 & 18.4 (two isomers). FT-IR: ν (cm^{-1}) 2927, 2855, 1603, 1561, 1509, 1413, 1346, 1215. HRMS [ESI] calcd for $C_{25}H_{38}NO$ [M+H]$^+$ 368.2948, found 368.2945.

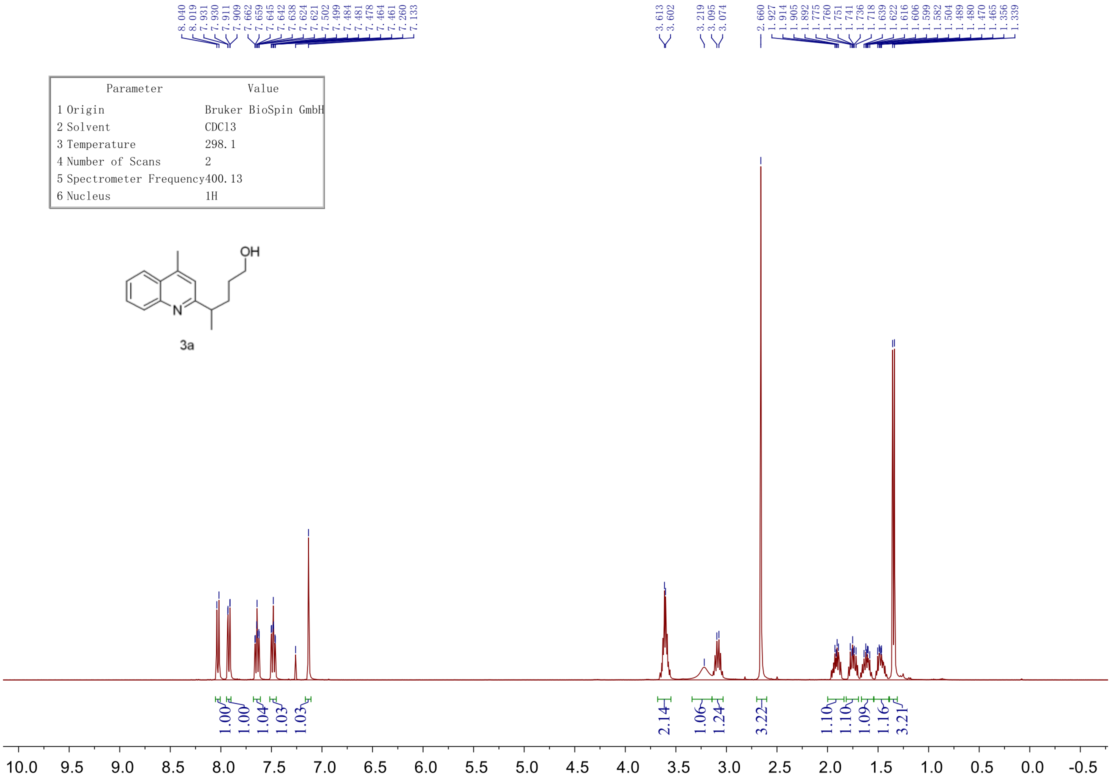

Supplementary Figure 2. ^{1}H NMR (400 MHz, $CDCl_3$) spectra for compound 3a.

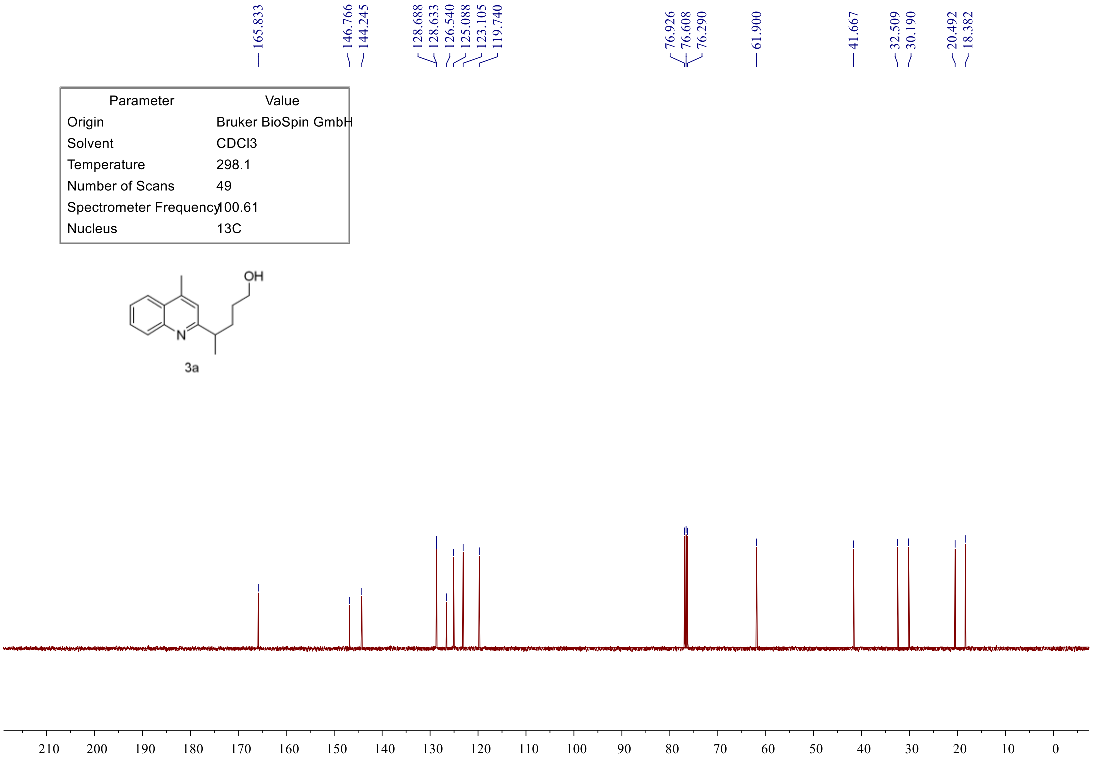

Supplementary Figure 3. ^{13}C NMR (100 MHz, $CDCl_3$) spectra for compound 3a.

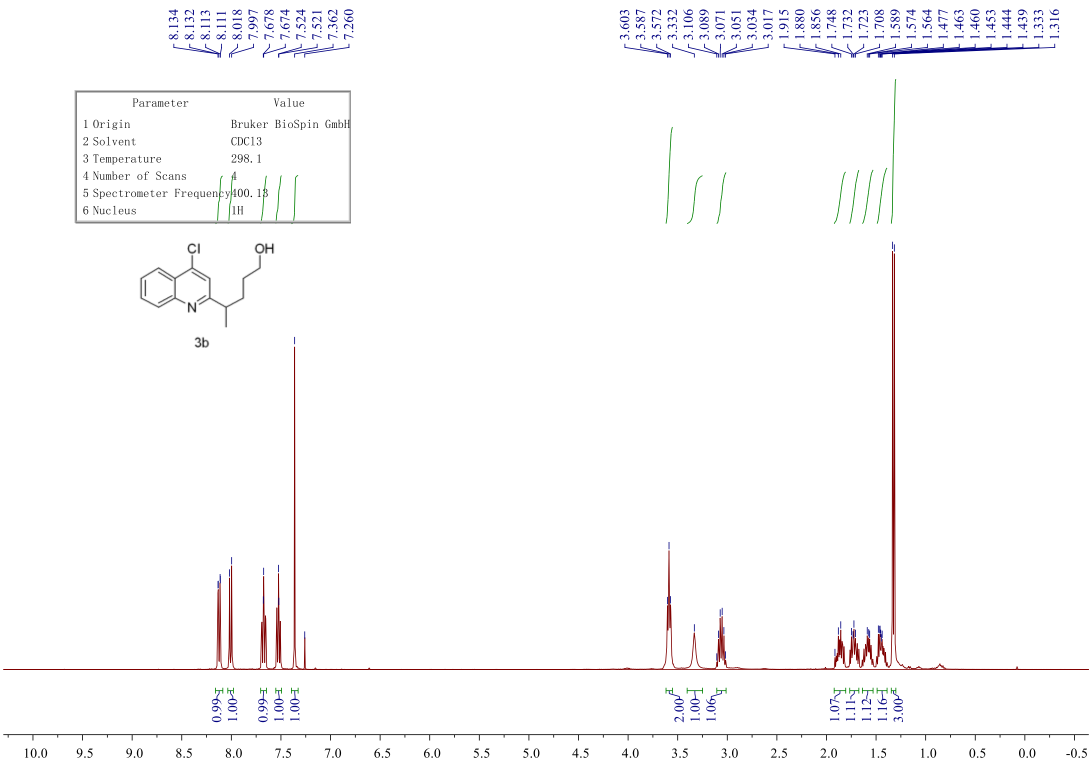

Supplementary Figure 4. ^{1}**H NMR (400 MHz,** $CDCl_3$**) spectra for compound 3b.**

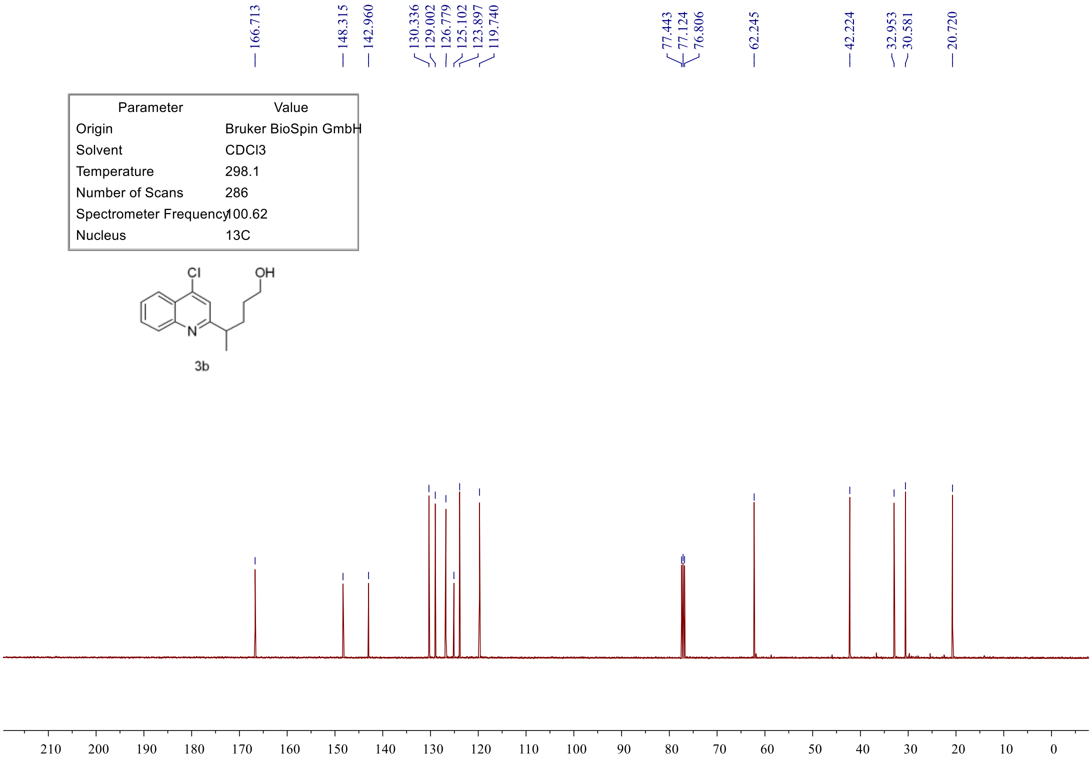

Supplementary Figure 5. ^{13}**C NMR (100 MHz,** $CDCl_3$**) spectra for compound 3b.**

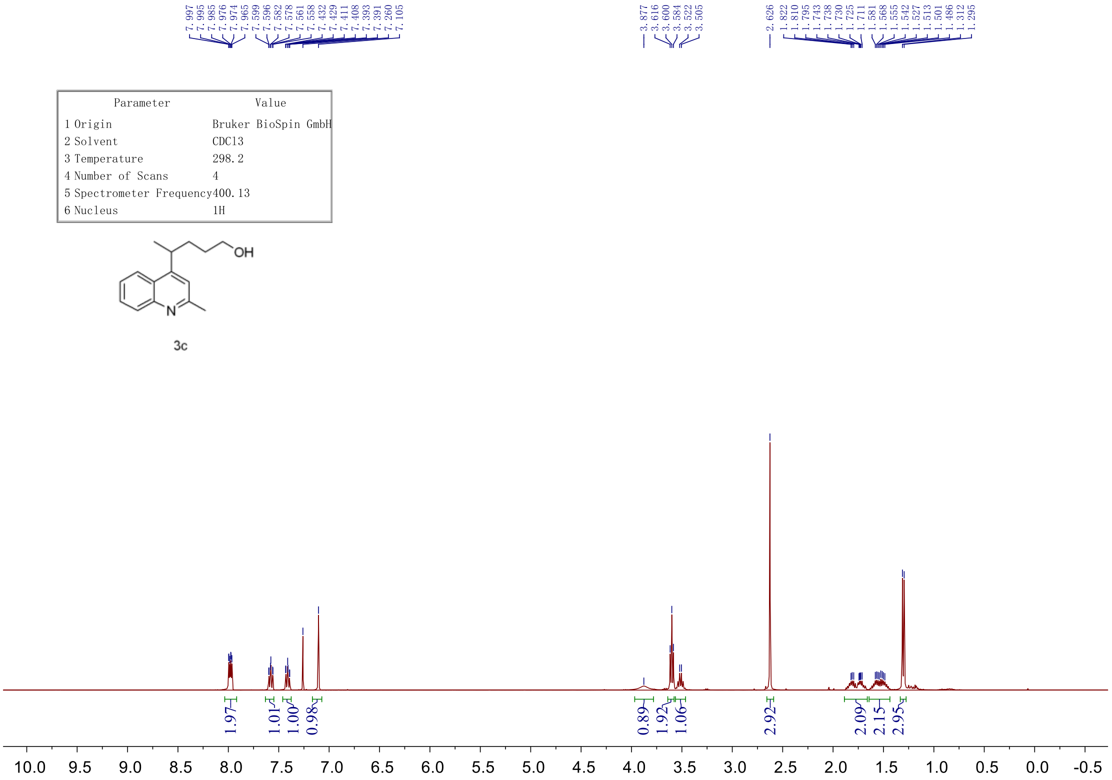

Supplementary Figure 6. ^{1}H **NMR (400 MHz,** $CDCl_3$**) spectra for compound 3c.**

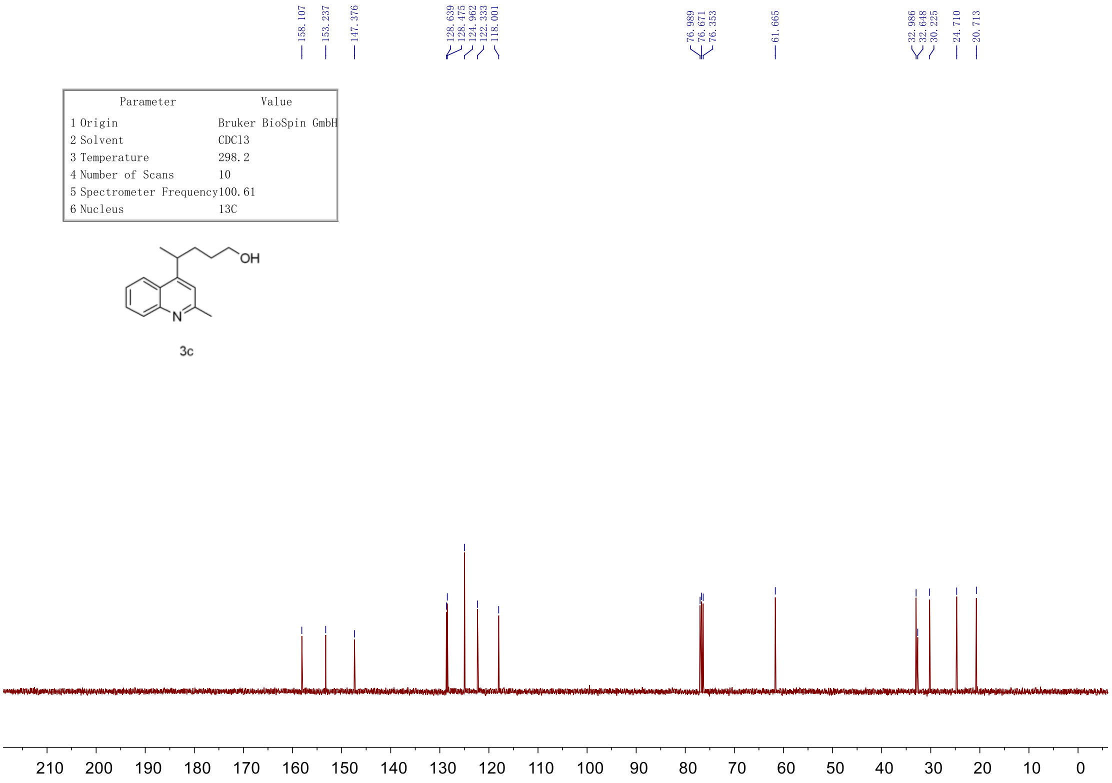

Supplementary Figure 7. ^{13}C **NMR (100 MHz,** $CDCl_3$**) spectra for compound 3c.**

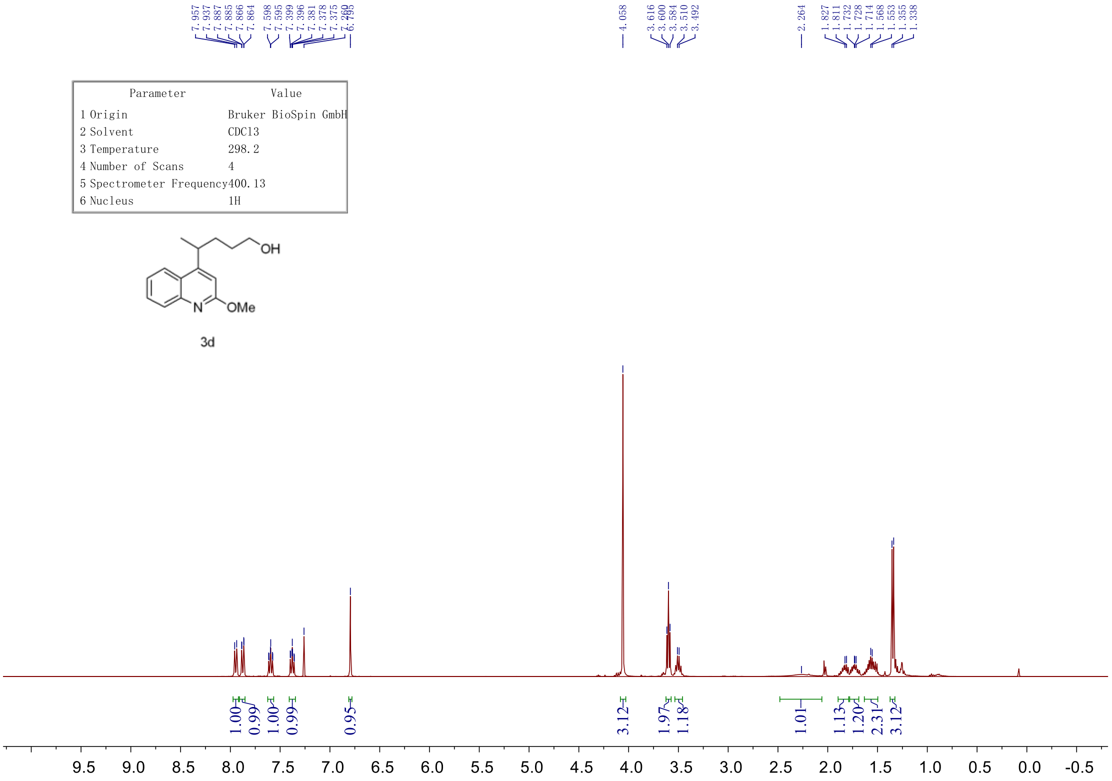

Supplementary Figure 8. ^{1}H NMR (400 MHz, $CDCl_3$) spectra for compound 3d.

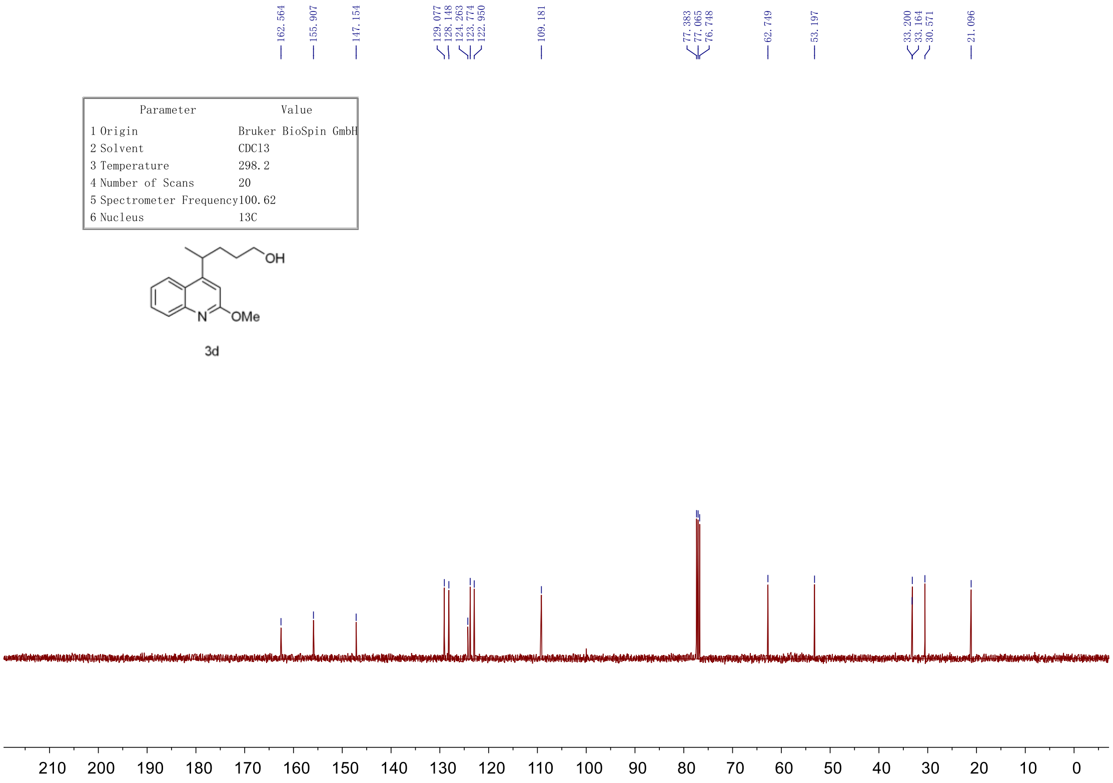

Supplementary Figure 9. ^{13}C NMR (100 MHz, $CDCl_3$) spectra for compound 3d.

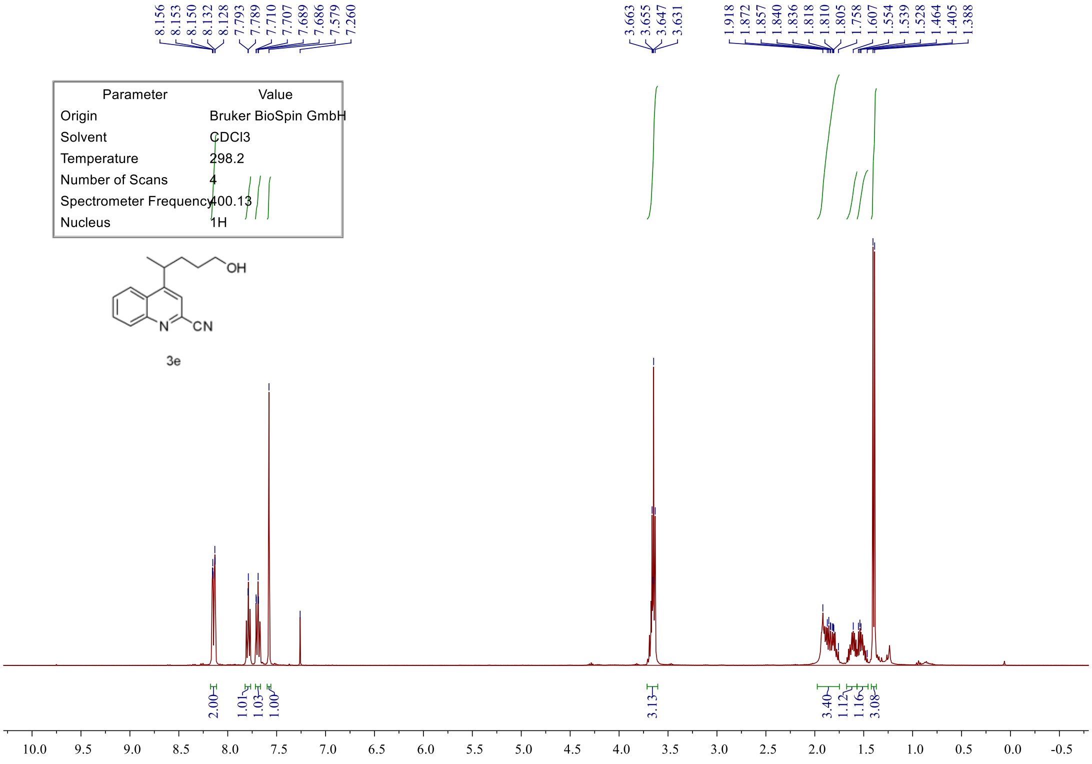

Supplementary Figure 10. ^{1}H NMR (400 MHz, $CDCl_3$) spectra for compound 3e.

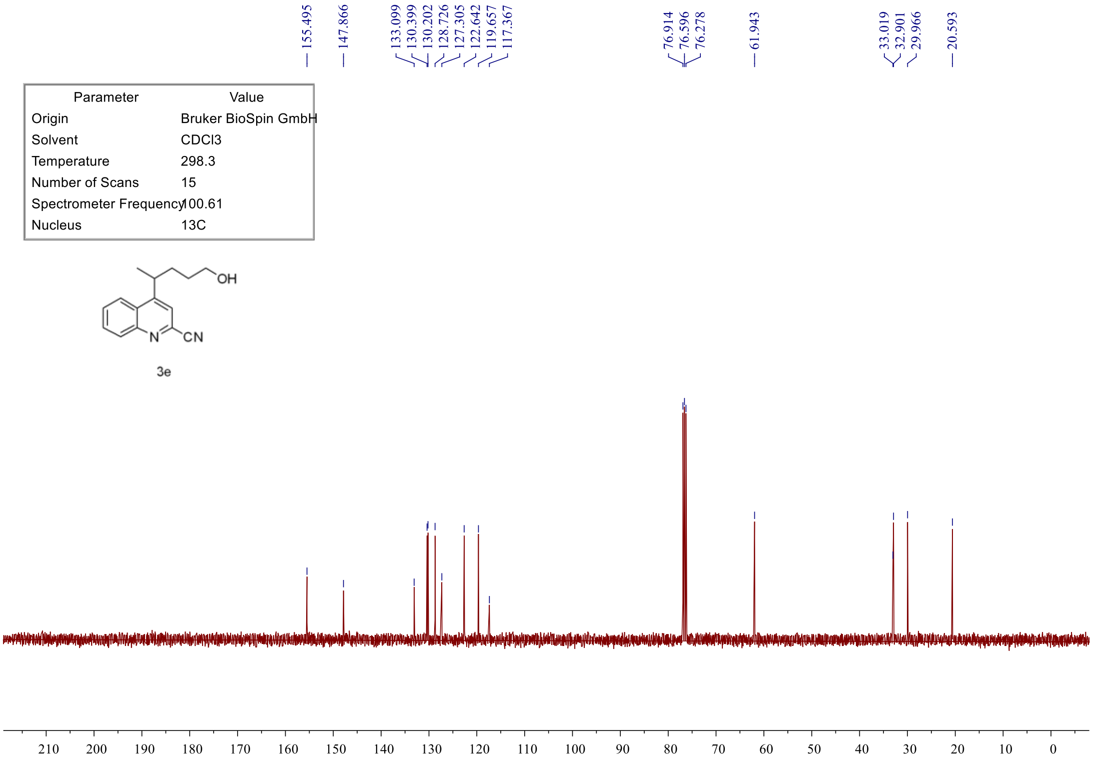

Supplementary Figure 11. ^{13}C NMR (100 MHz, $CDCl_3$) spectra for compound 3e.

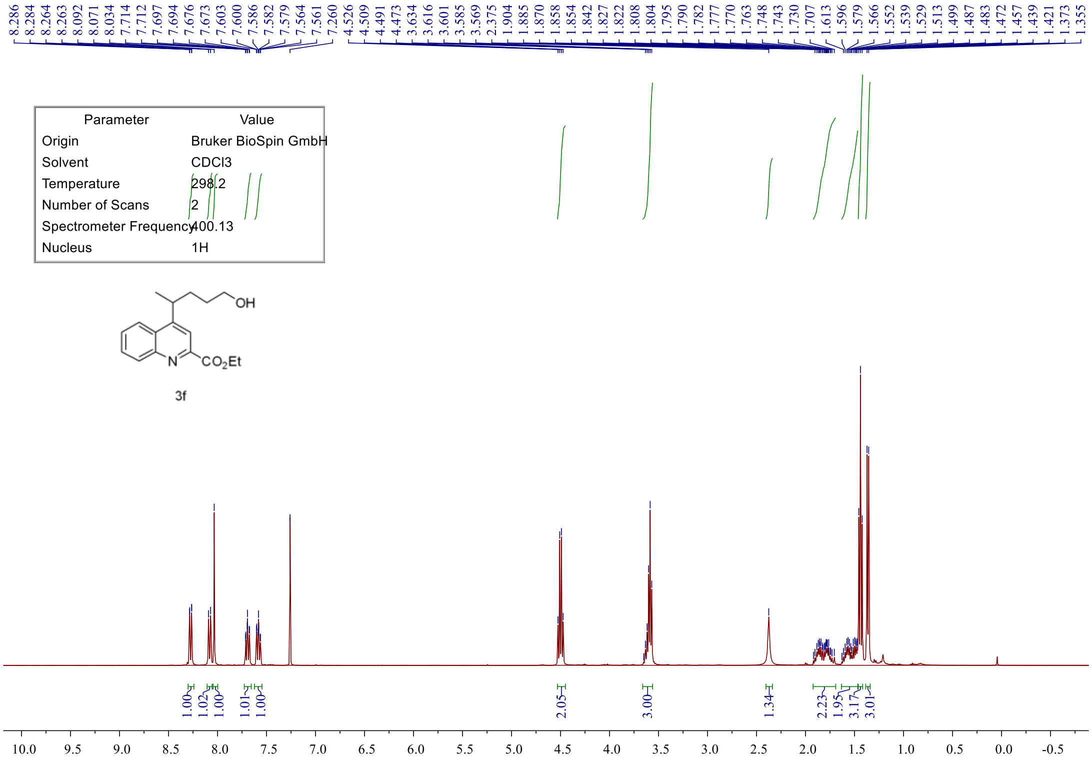

Supplementary Figure 12. ^{1}H NMR (400 MHz, $CDCl_3$) spectra for compound 3f.

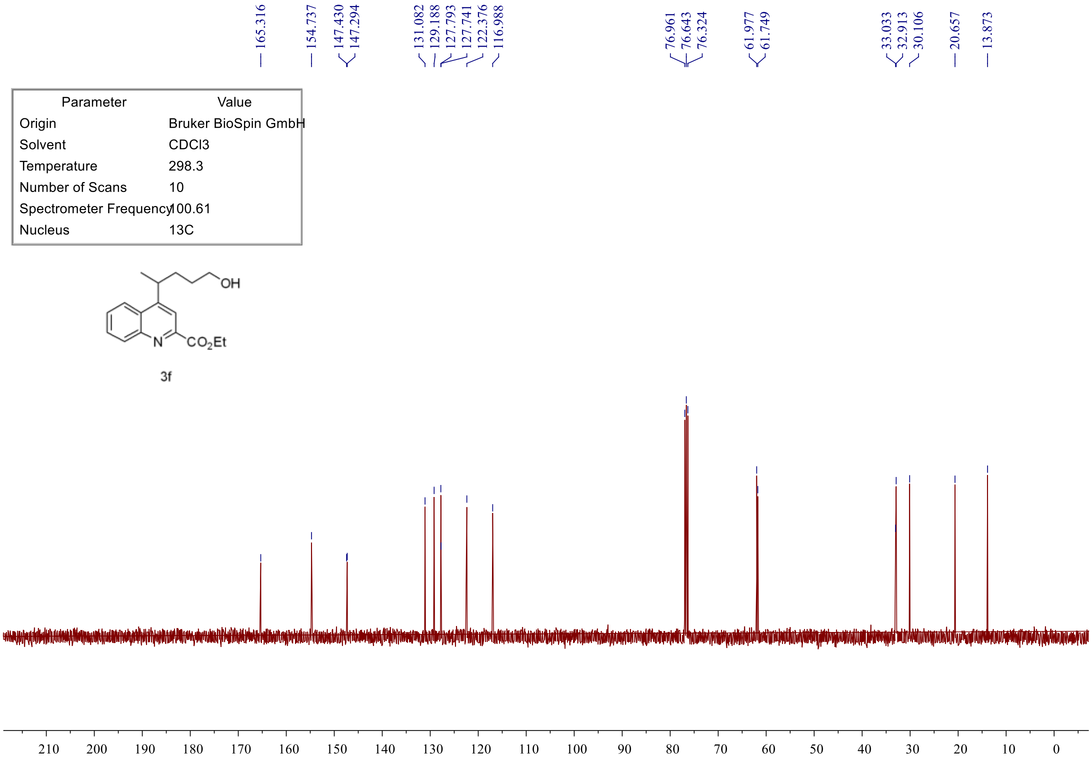

Supplementary Figure 13. ^{13}C NMR (100 MHz, $CDCl_3$) spectra for compound 3f.

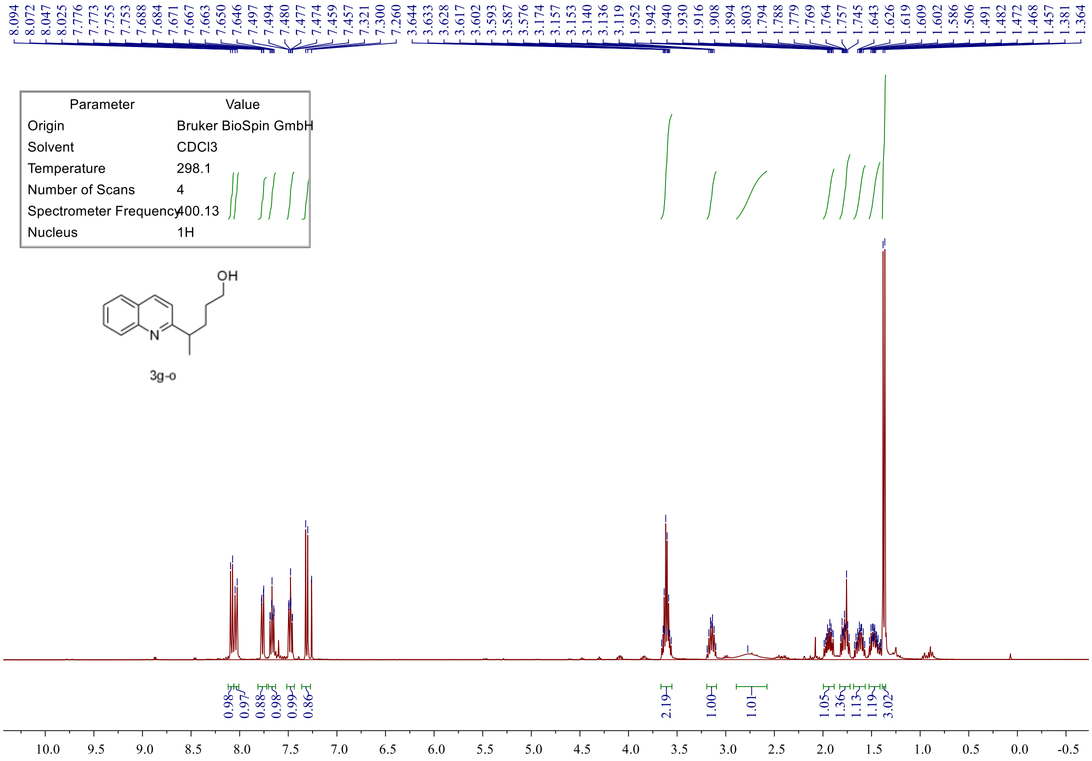

Supplementary Figure 14. **^{1}H NMR (400 MHz, $CDCl_3$) spectra for compound 3g-o.**

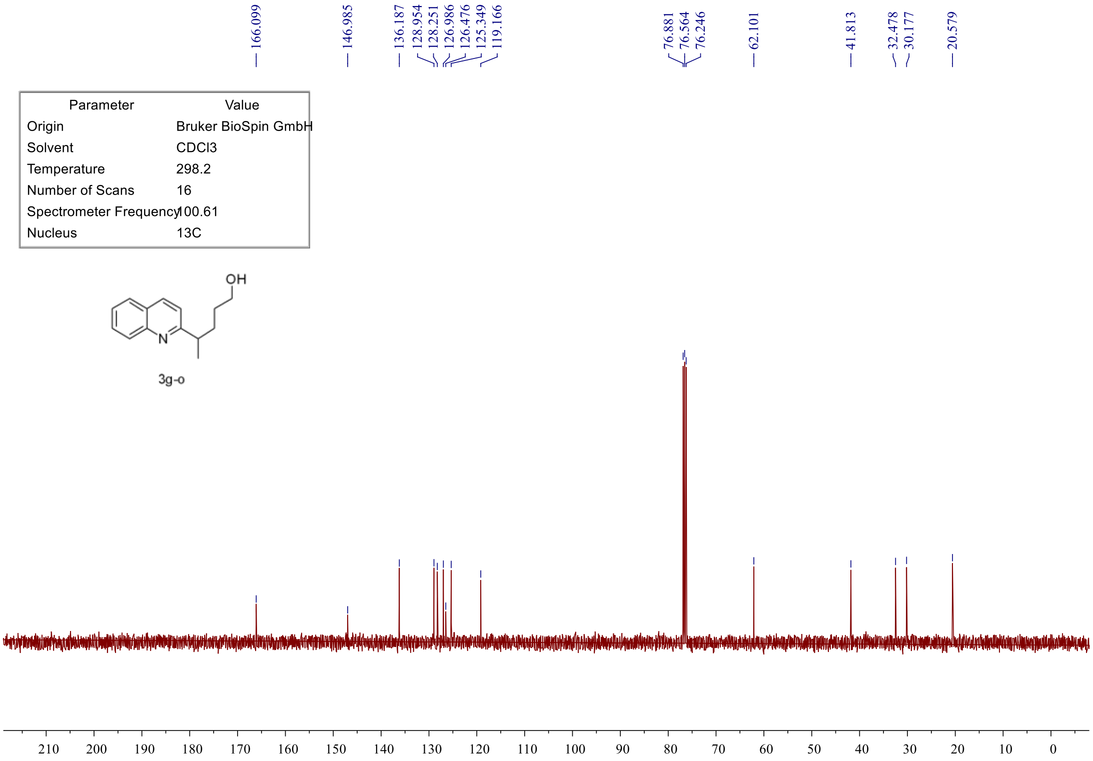

Supplementary Figure 15. **^{13}C NMR (100 MHz, $CDCl_3$) spectra for compound 3g-o.**

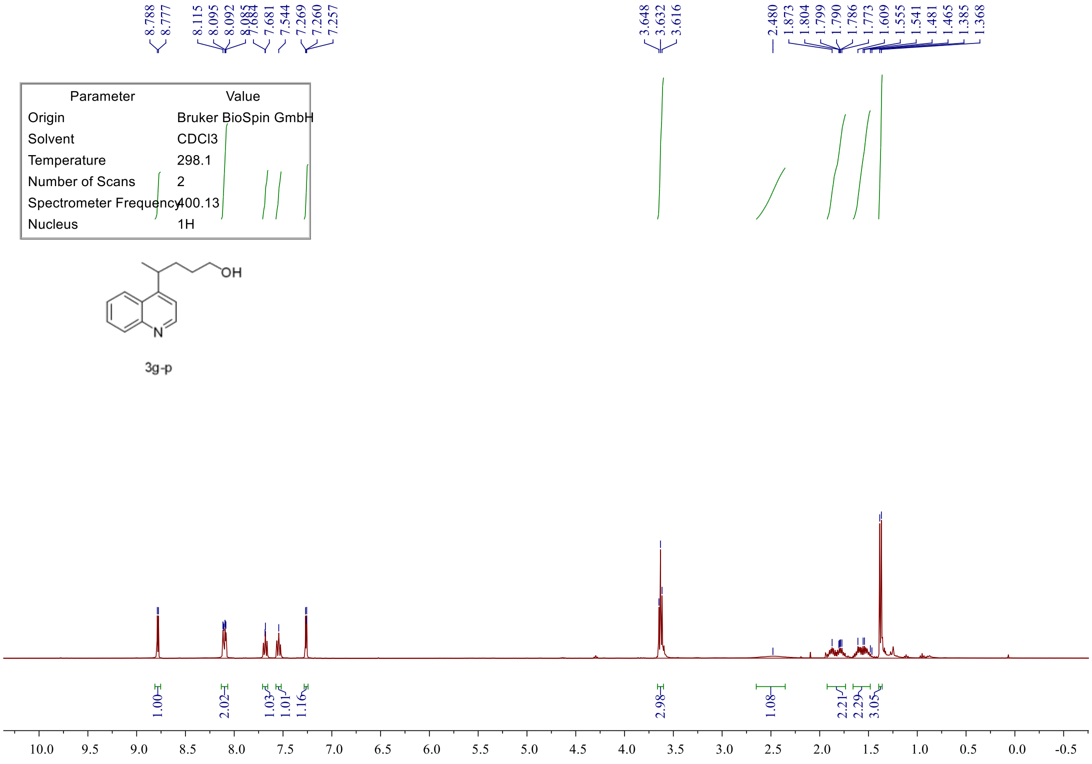

Supplementary Figure 16. ^{1}H NMR (400 MHz, $CDCl_3$) spectra for compound 3g-p.

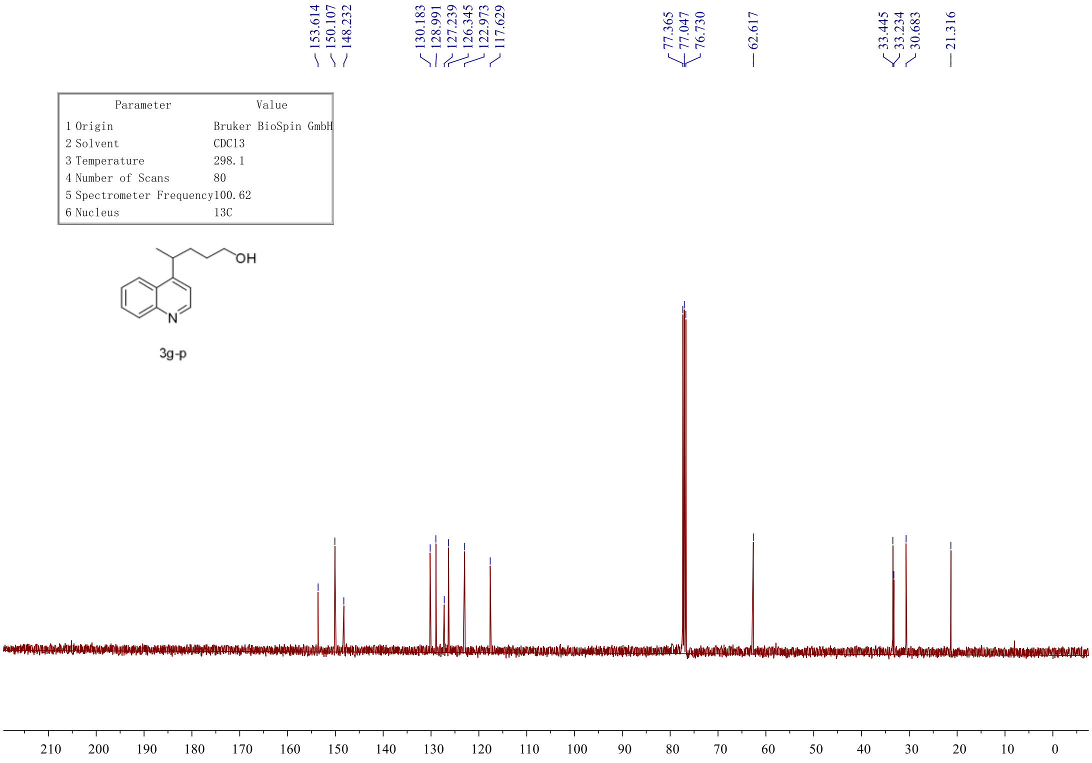

Supplementary Figure 17. ^{13}C NMR (100 MHz, $CDCl_3$) spectra for compound 3g-p.

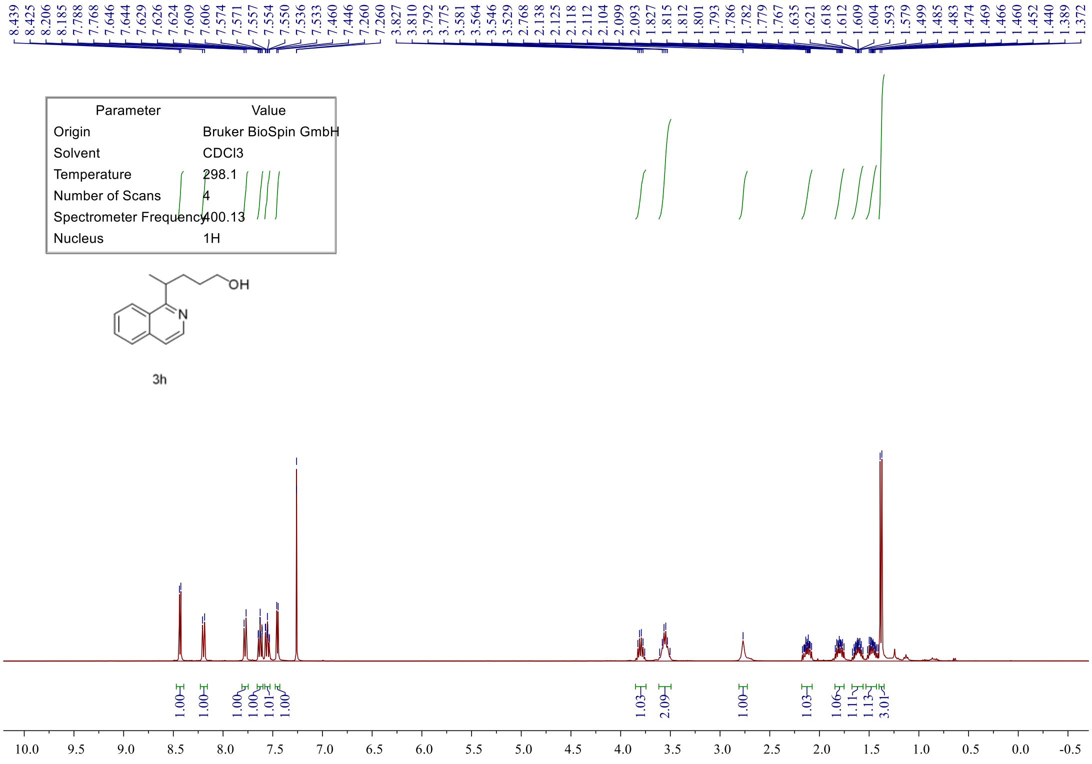

Supplementary Figure 18. ^{1}H NMR (400 MHz, $CDCl_3$) spectra for compound 3h.

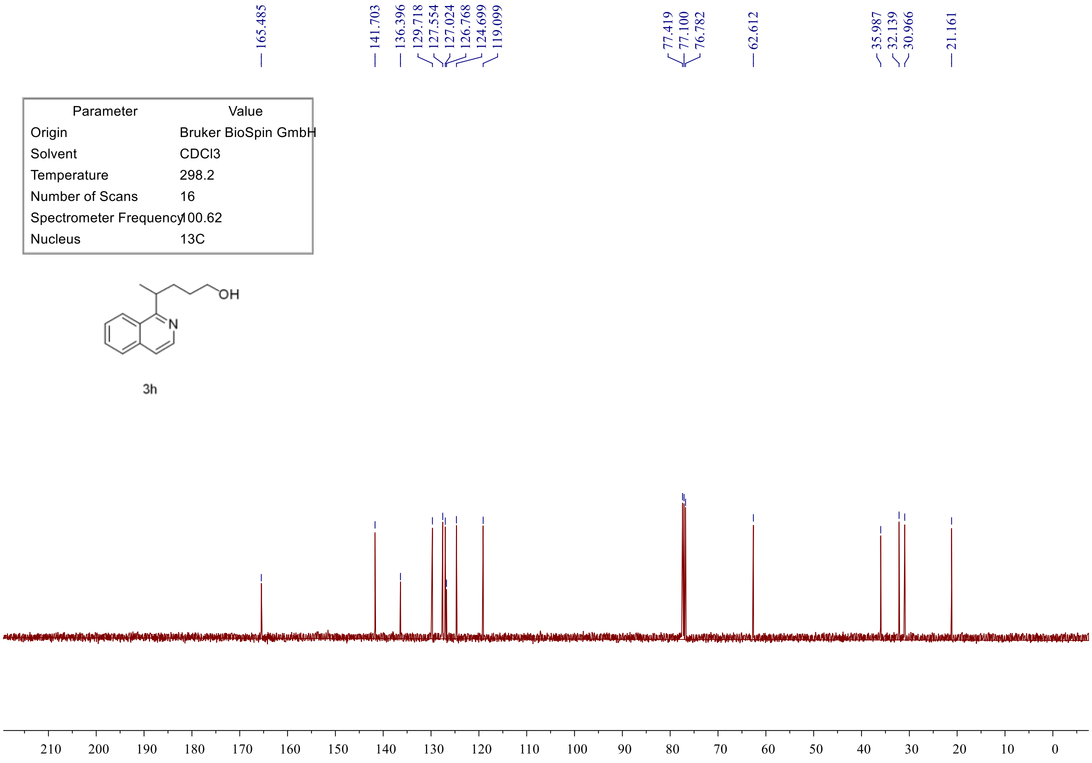

Supplementary Figure 19. ^{13}C NMR (100 MHz, $CDCl_3$) spectra for compound 3h.

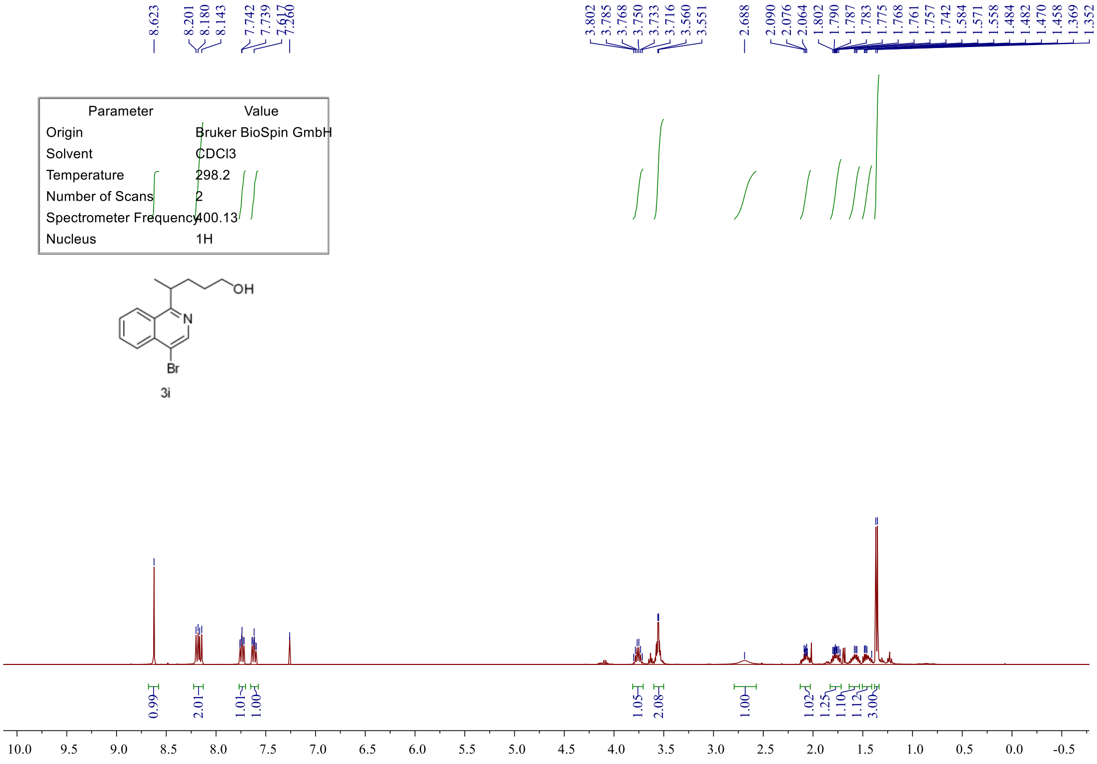

Supplementary Figure 20. ^{1}**H NMR (400 MHz, CDCl$_3$) spectra for compound 3i.**

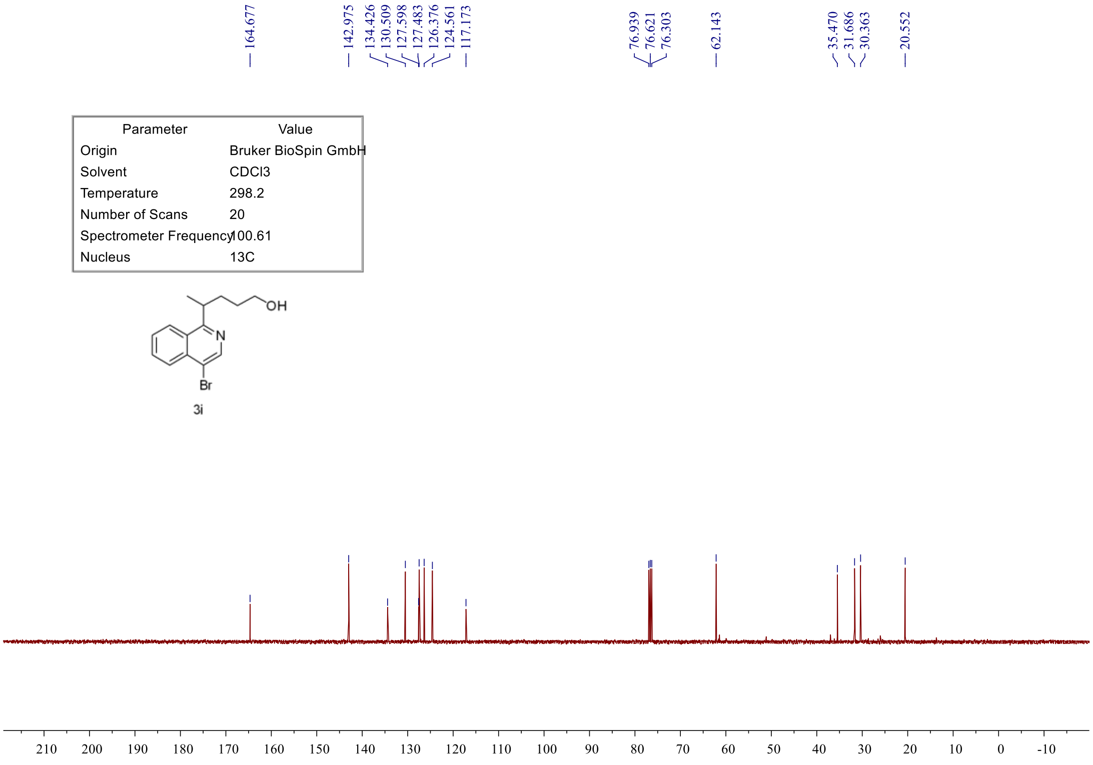

Supplementary Figure 21. ^{13}**C NMR (100 MHz, CDCl$_3$) spectra for compound 3i.**

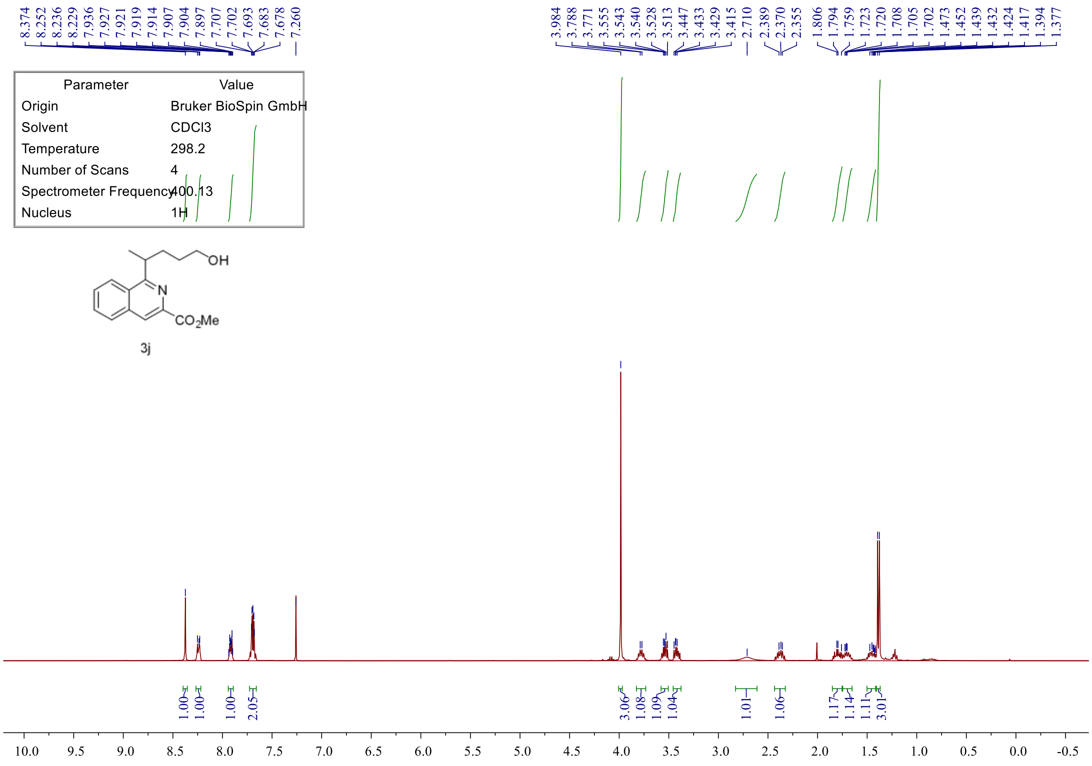

Supplementary Figure 22. ^{1}H NMR (400 MHz, $CDCl_3$) spectra for compound 3j.

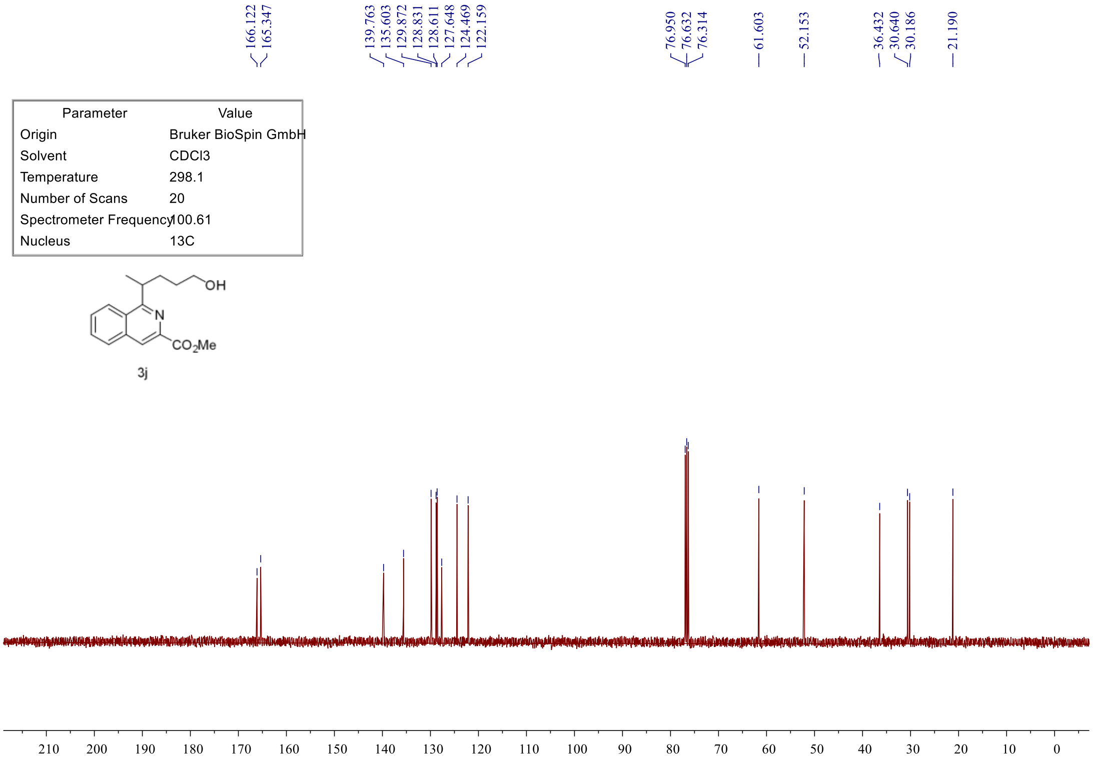

Supplementary Figure 23. ^{13}C NMR (100 MHz, $CDCl_3$) spectra for compound 3j.

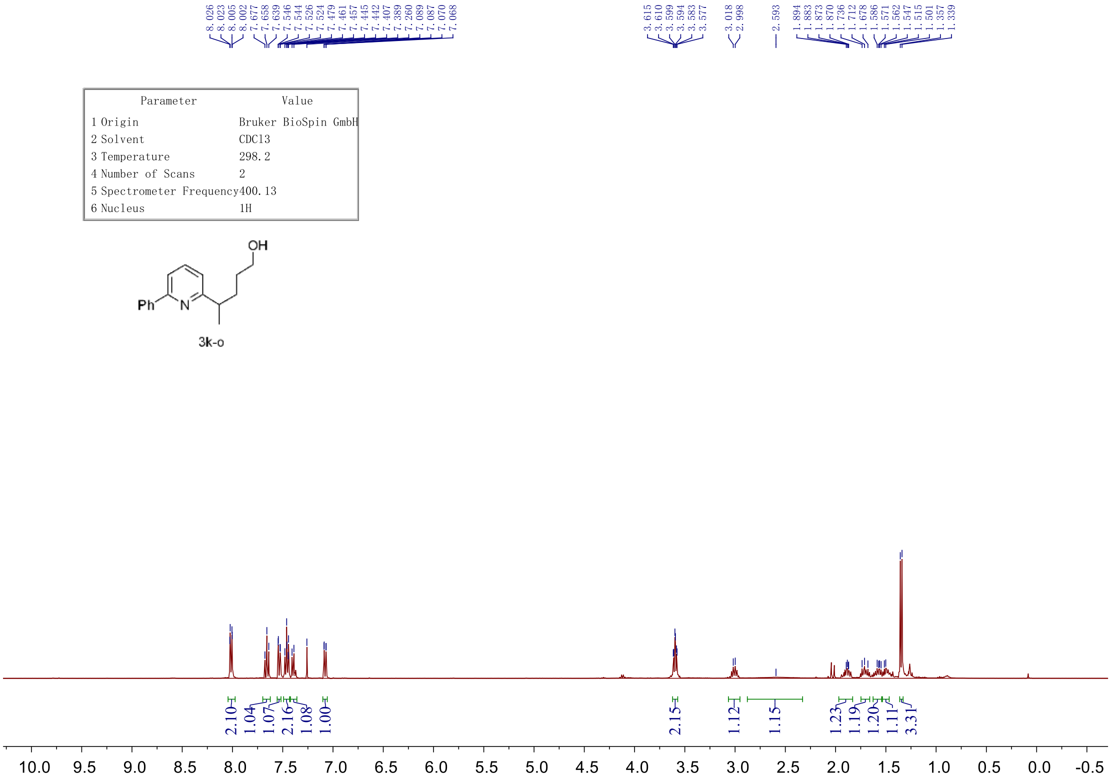

Supplementary Figure 24. ^{1}H NMR (400 MHz, $CDCl_3$) spectra for compound 3k-o.

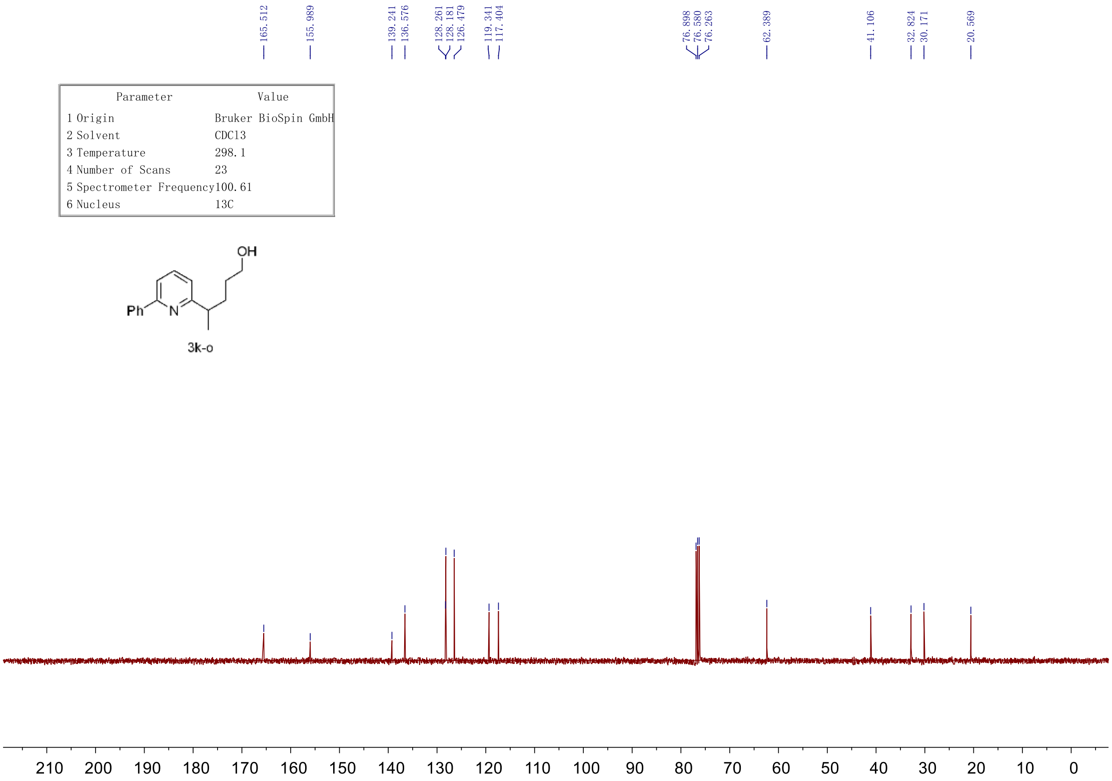

Supplementary Figure 25. ^{13}C NMR (100 MHz, $CDCl_3$) spectra for compound 3k-o.

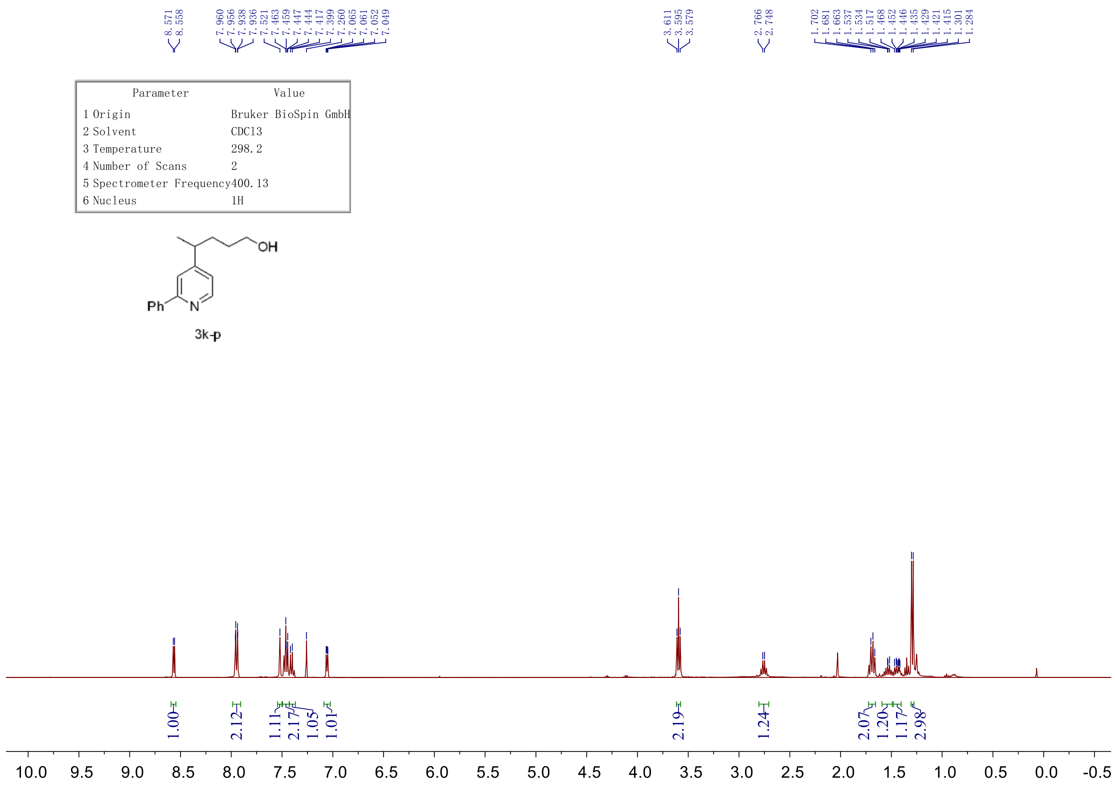

Supplementary Figure 26. ^{1}H **NMR (400 MHz,** $CDCl_3$**) spectra for compound 3k-p.**

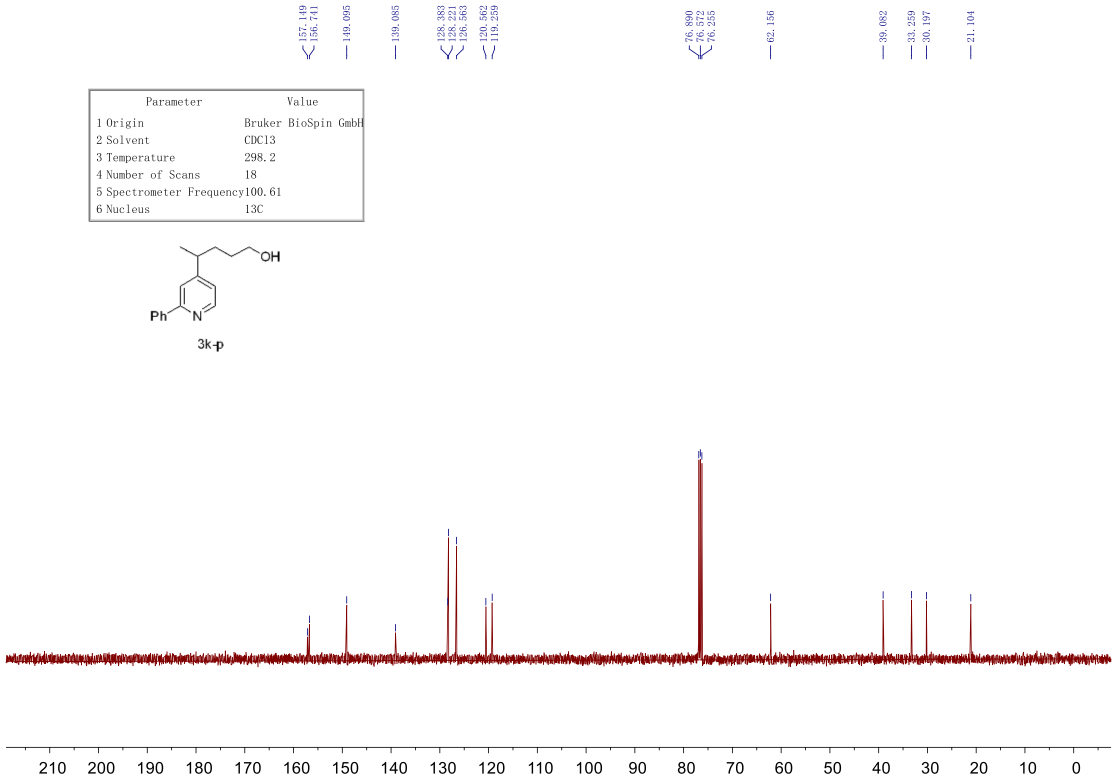

Supplementary Figure 27. ^{13}C **NMR (100 MHz,** $CDCl_3$**) spectra for compound 3k-p.**

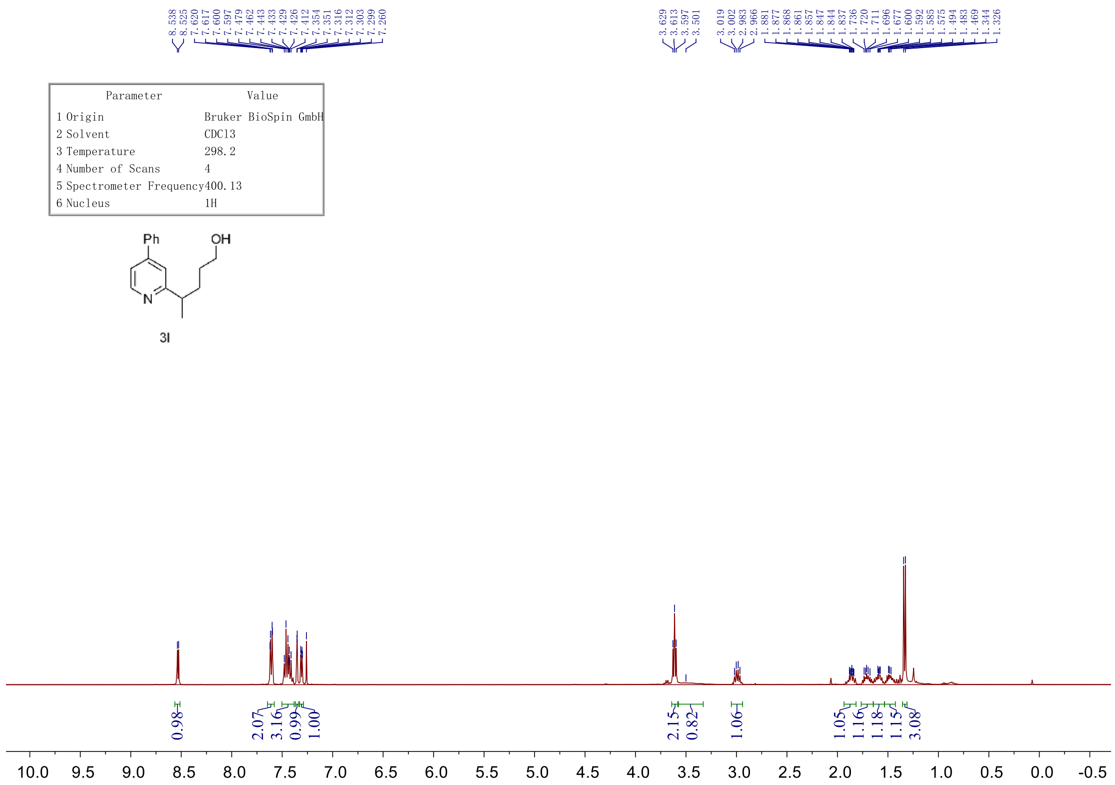

Supplementary Figure 28. ^{1}H NMR (400 MHz, $CDCl_3$) spectra for compound 3l.

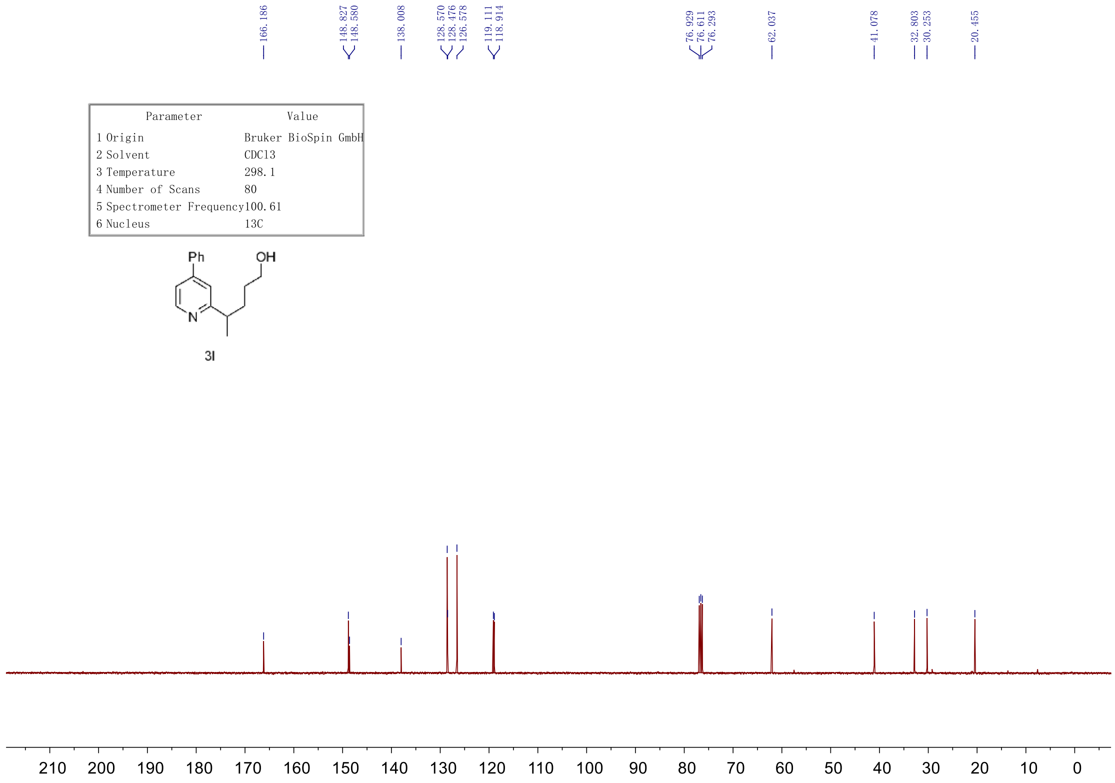

Supplementary Figure 29. ^{13}C NMR (100 MHz, $CDCl_3$) spectra for compound 3l.

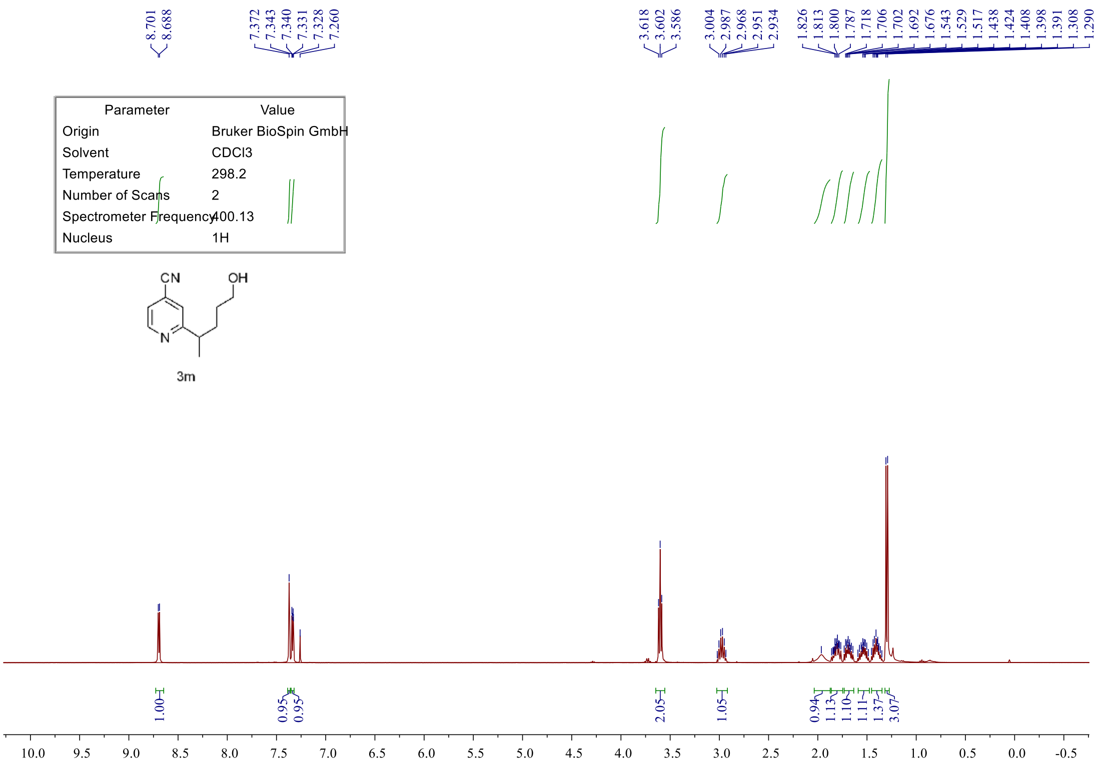

Supplementary Figure 30. ^{1}H NMR (400 MHz, $CDCl_3$) spectra for compound 3m.

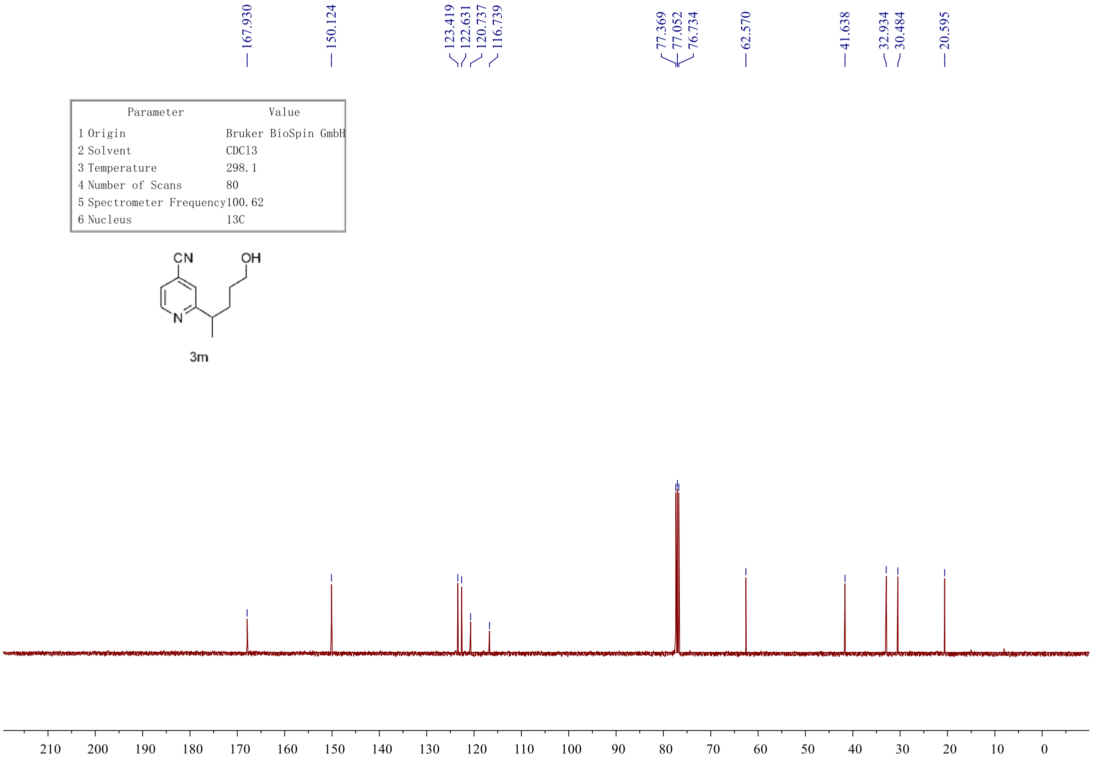

Supplementary Figure 31. ^{13}C NMR (100 MHz, $CDCl_3$) spectra for compound 3m.

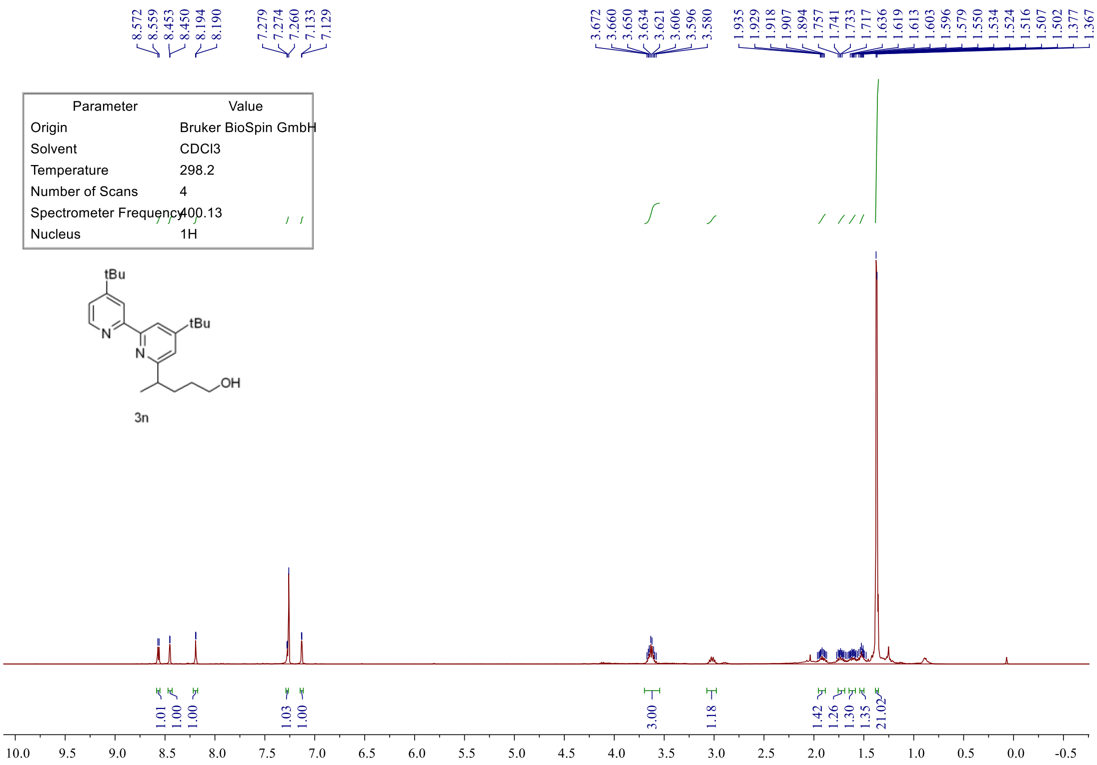

Supplementary Figure 32. ^{1}H NMR (400 MHz, $CDCl_3$) spectra for compound 3n.

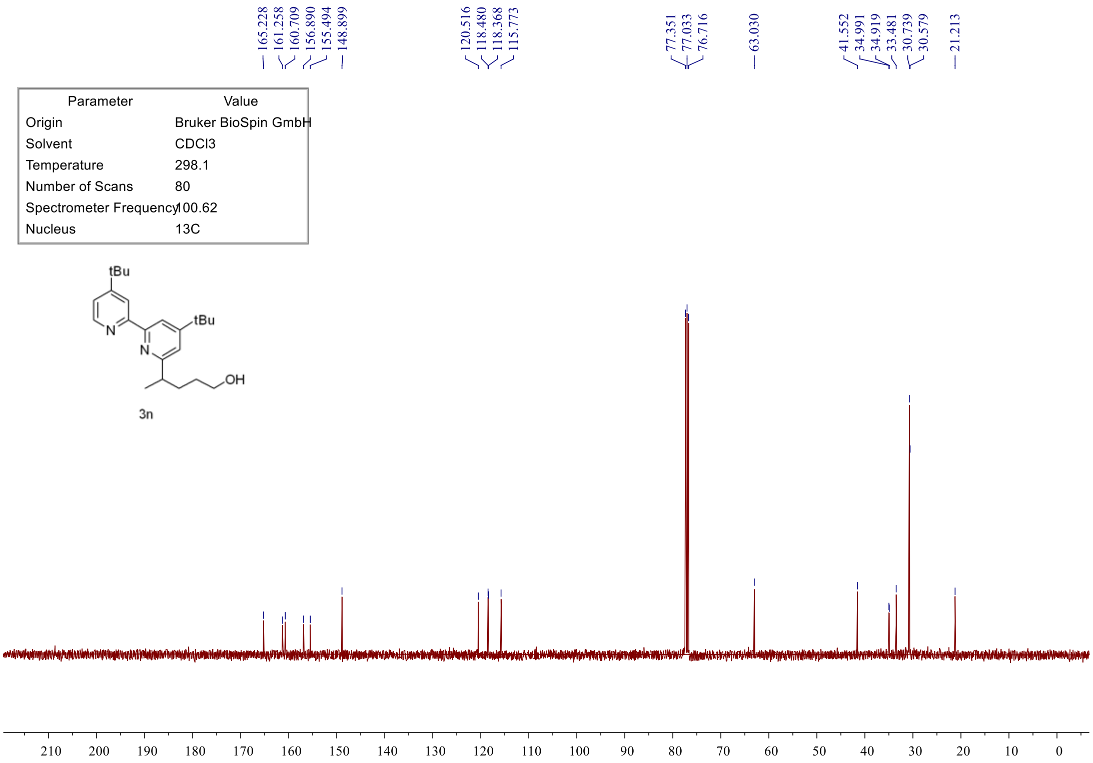

Supplementary Figure 33. ^{13}C NMR (100 MHz, $CDCl_3$) spectra for compound 3n.

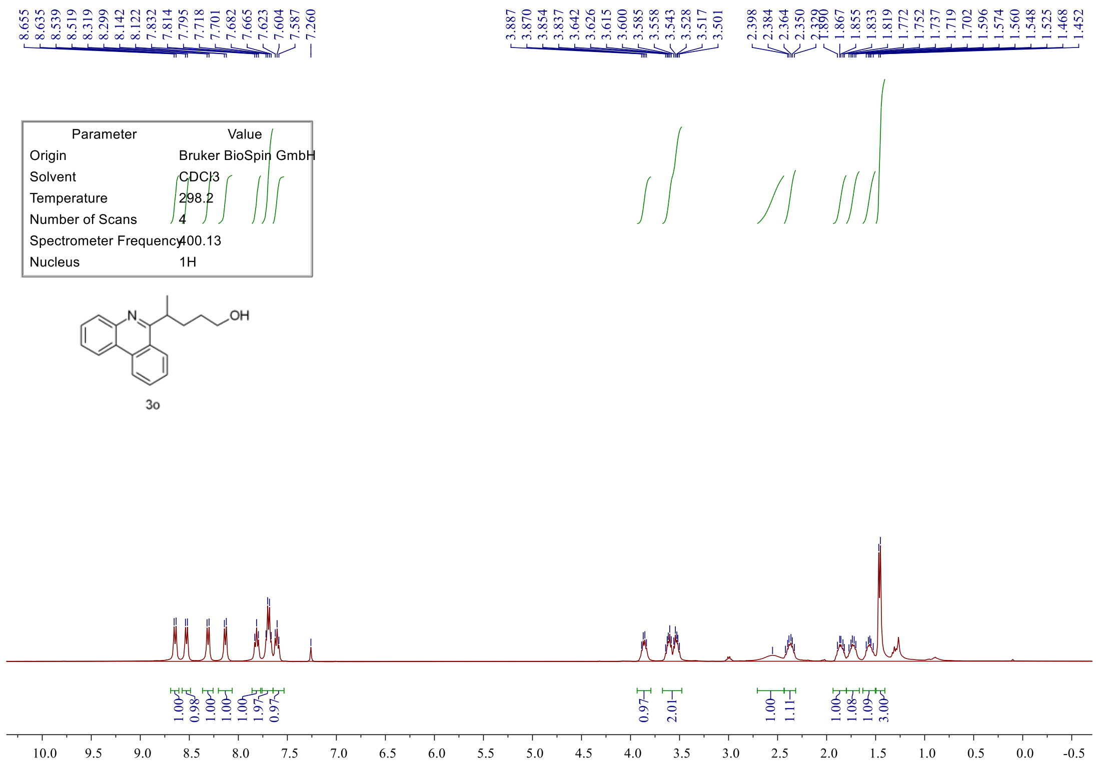

Supplementary Figure 34. ^{1}H NMR (400 MHz, $CDCl_3$) spectra for compound 3o.

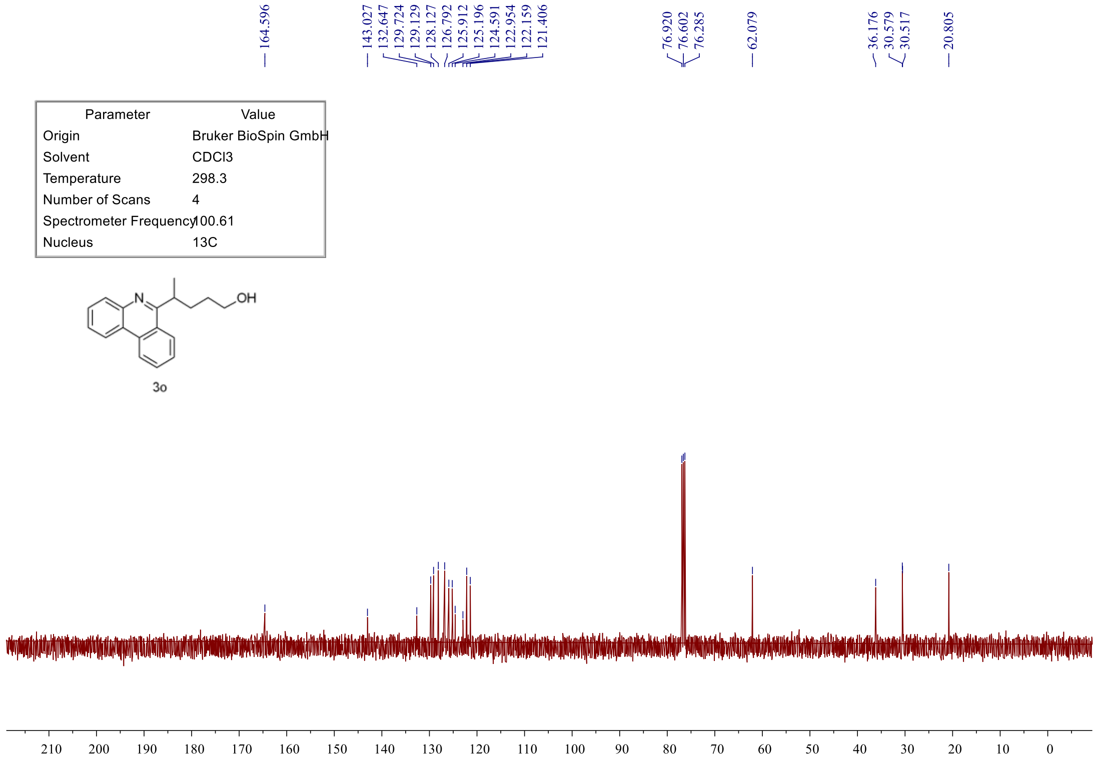

Supplementary Figure 35. ^{13}C NMR (100 MHz, $CDCl_3$) spectra for compound 3o.

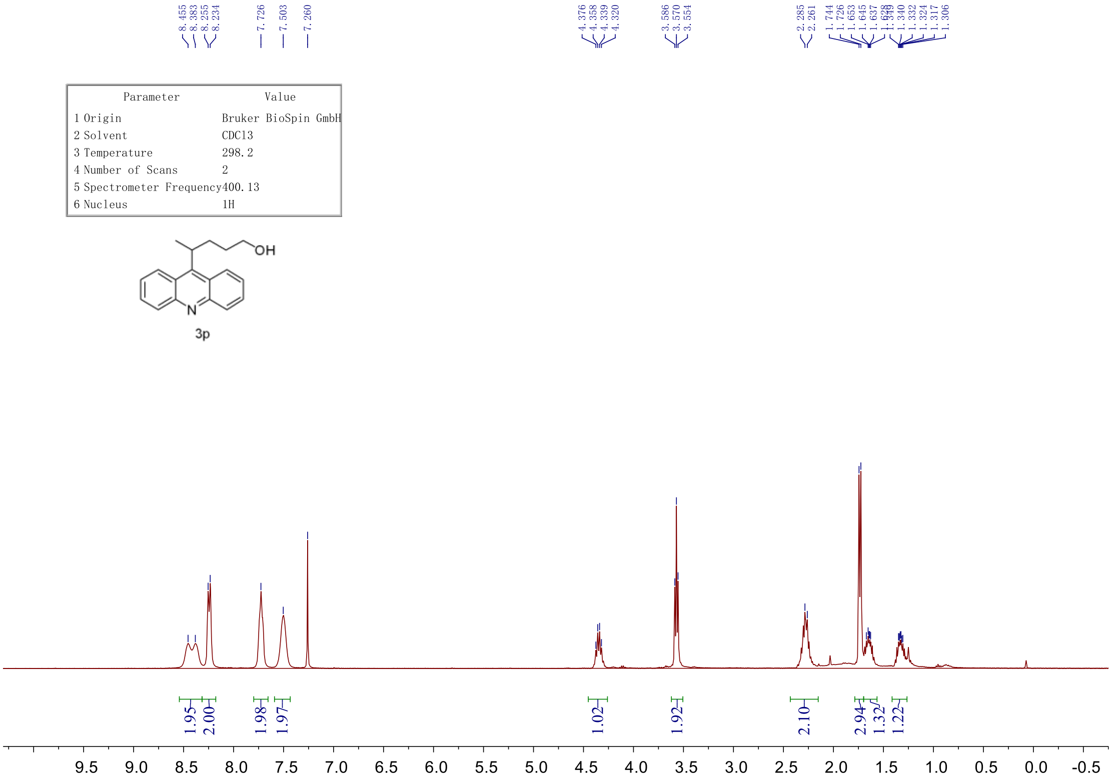

Supplementary Figure 36. ^{1}H NMR (400 MHz, $CDCl_3$) spectra for compound 3p.

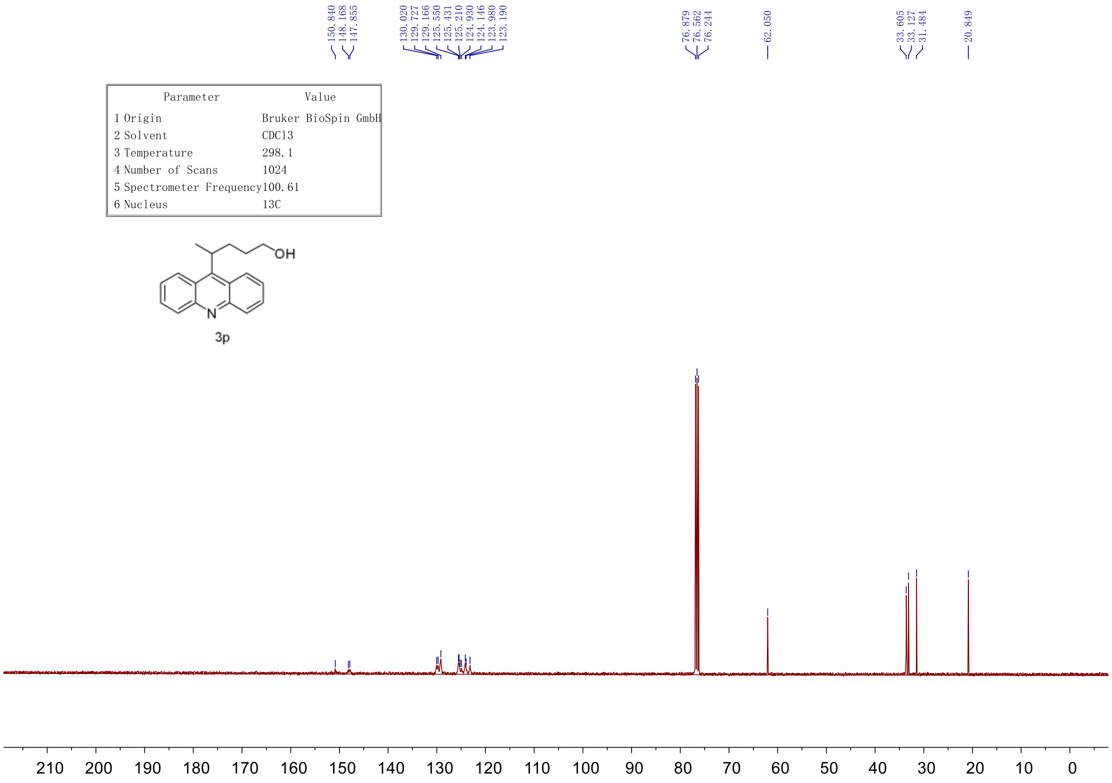

Supplementary Figure 37. ^{13}C NMR (100 MHz, $CDCl_3$) spectra for compound 3p.

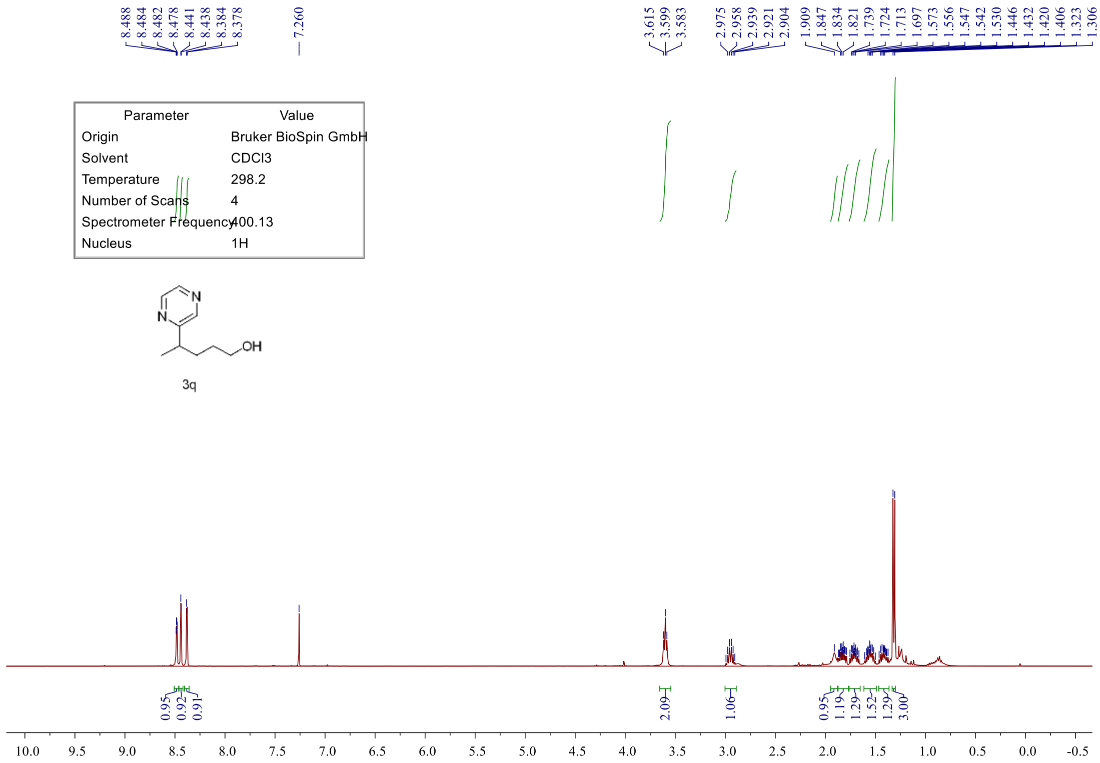

Supplementary Figure 38. ^{1}H NMR (400 MHz, $CDCl_3$) spectra for compound 3q.

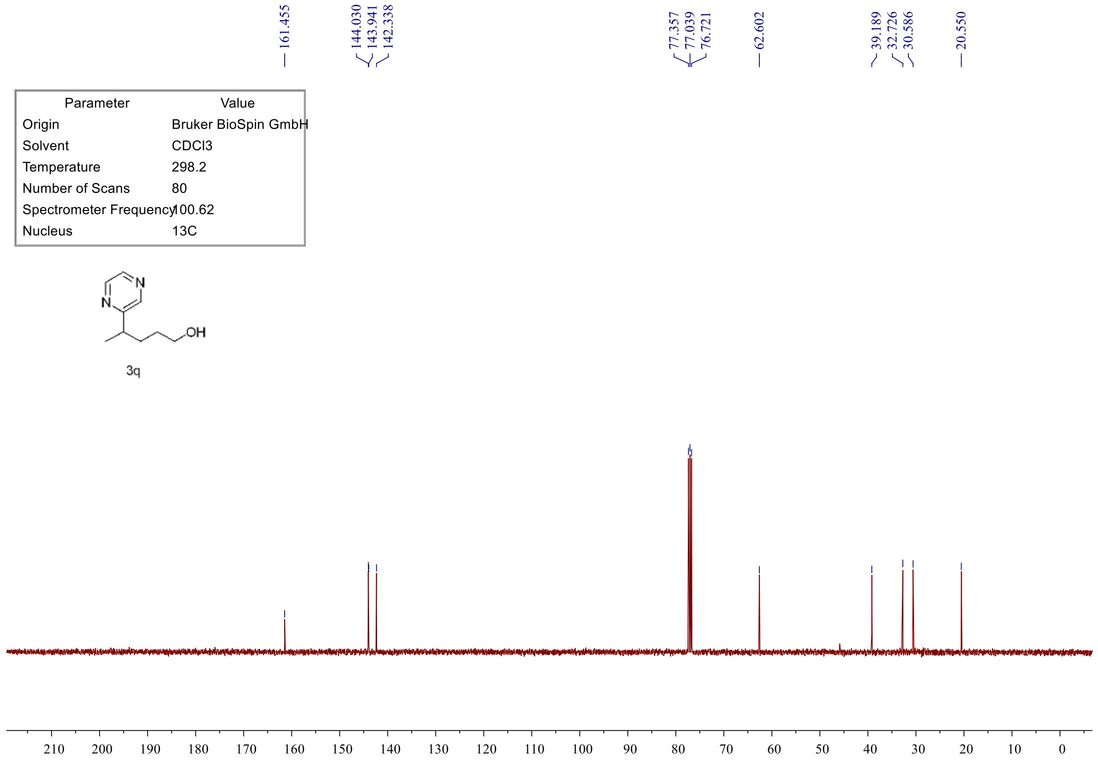

Supplementary Figure 39. ^{13}C NMR (100 MHz, $CDCl_3$) spectra for compound 3q.

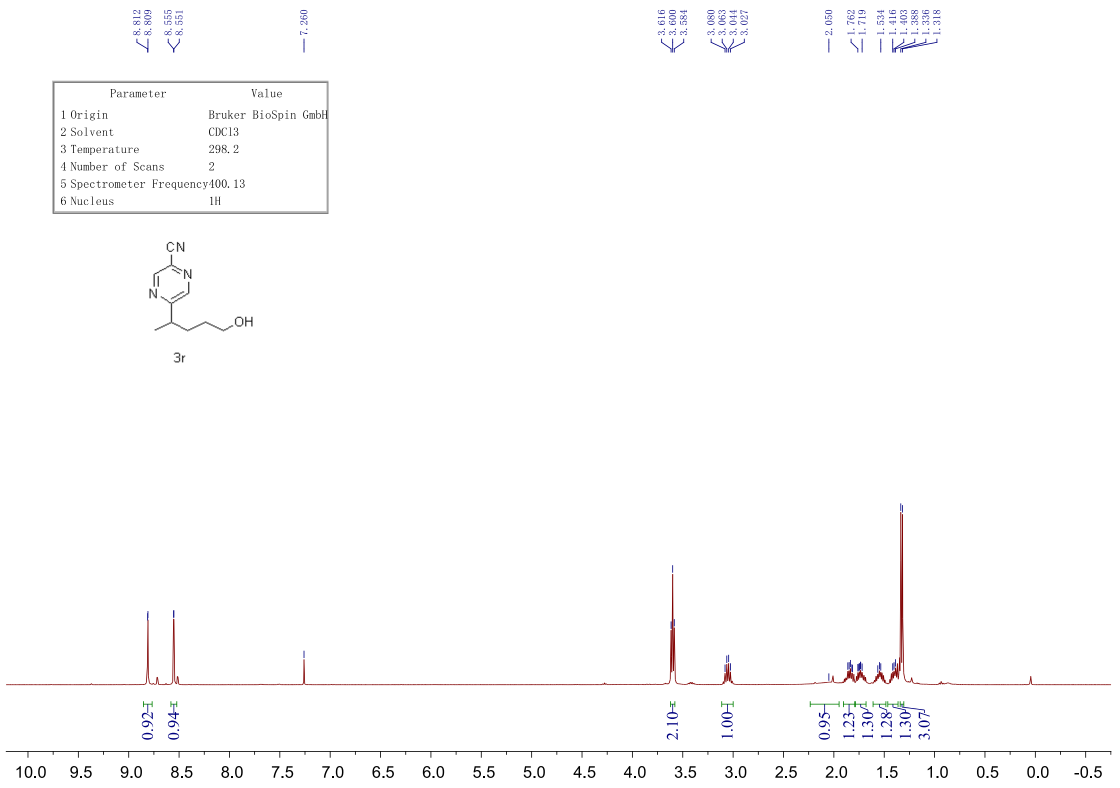

Supplementary Figure 40. ^{1}H **NMR (400 MHz,** $CDCl_3$**) spectra for compound 3r.**

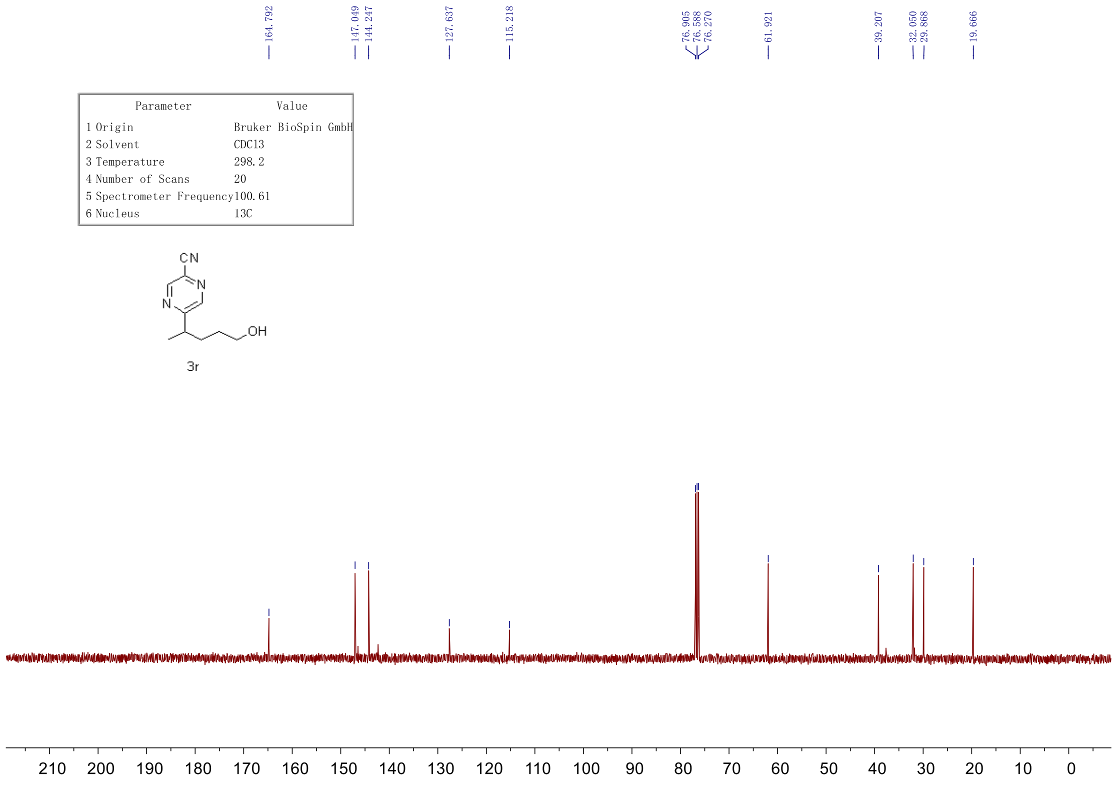

Supplementary Figure 41. ^{13}C **NMR (100 MHz,** $CDCl_3$**) spectra for compound 3r.**

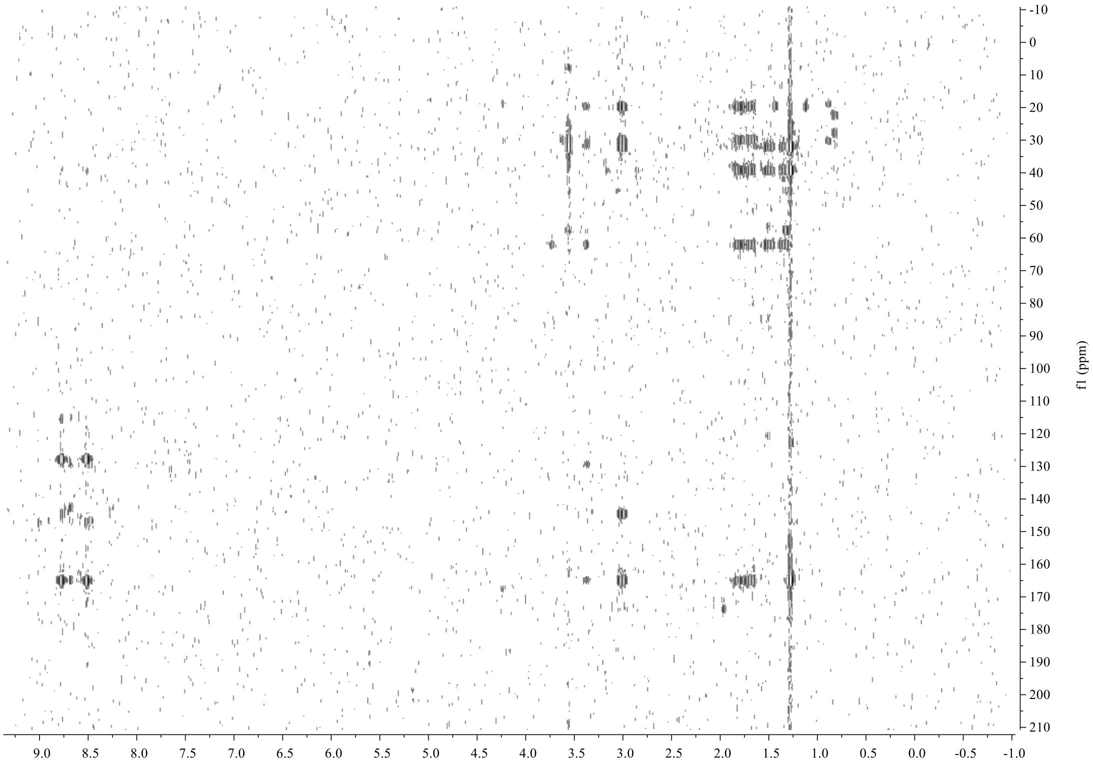

Supplementary Figure 42. HMBC spectra for compound 3r.

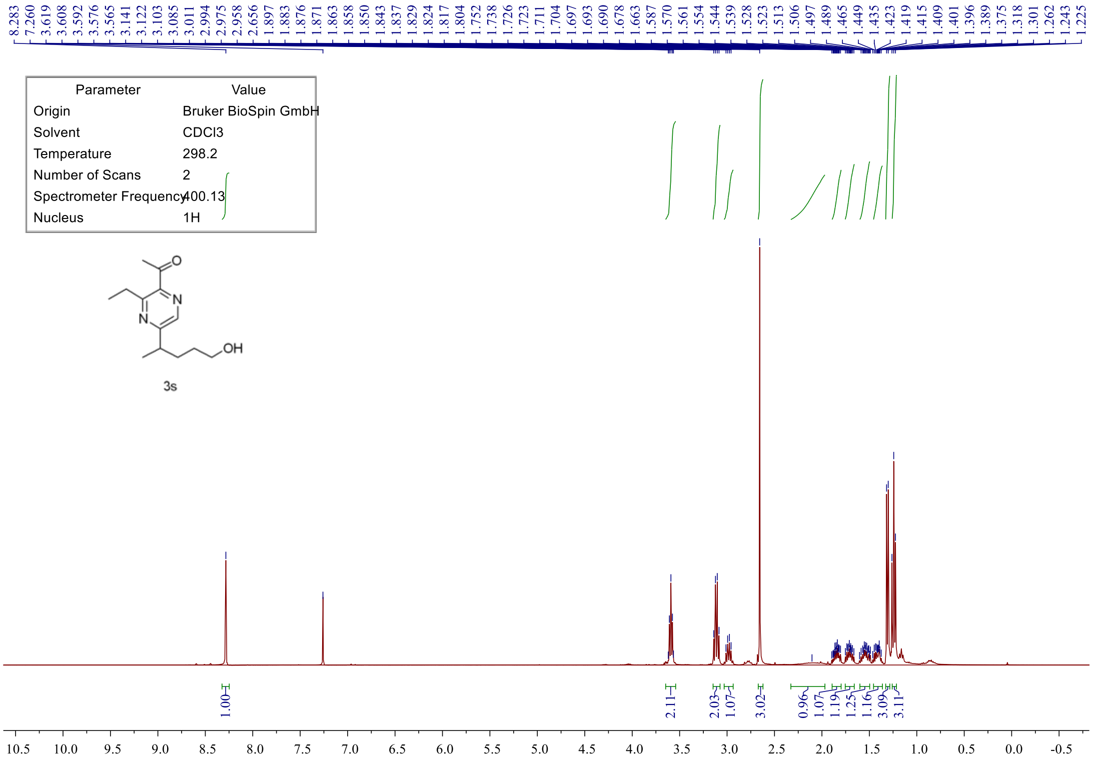

Supplementary Figure 43. ^{1}H NMR (400 MHz, $CDCl_3$) spectra for compound 3s.

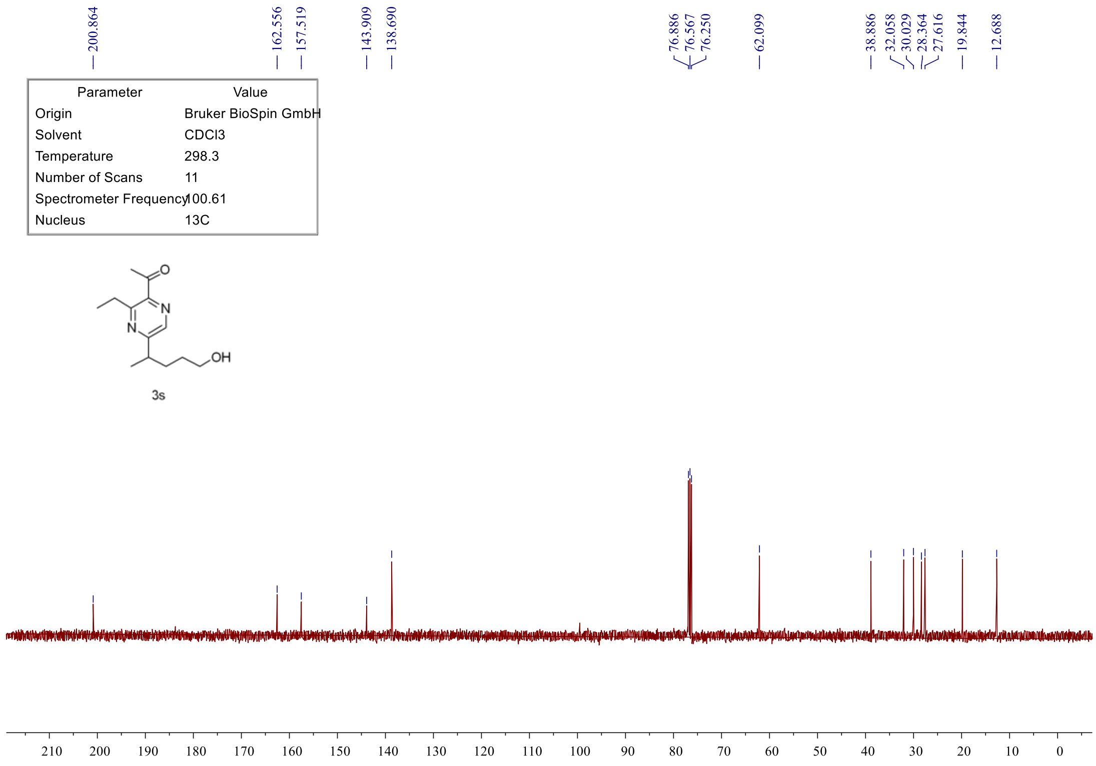

Supplementary Figure 44. ^{13}**C NMR (100 MHz, CDCl$_3$) spectra for compound 3s.**

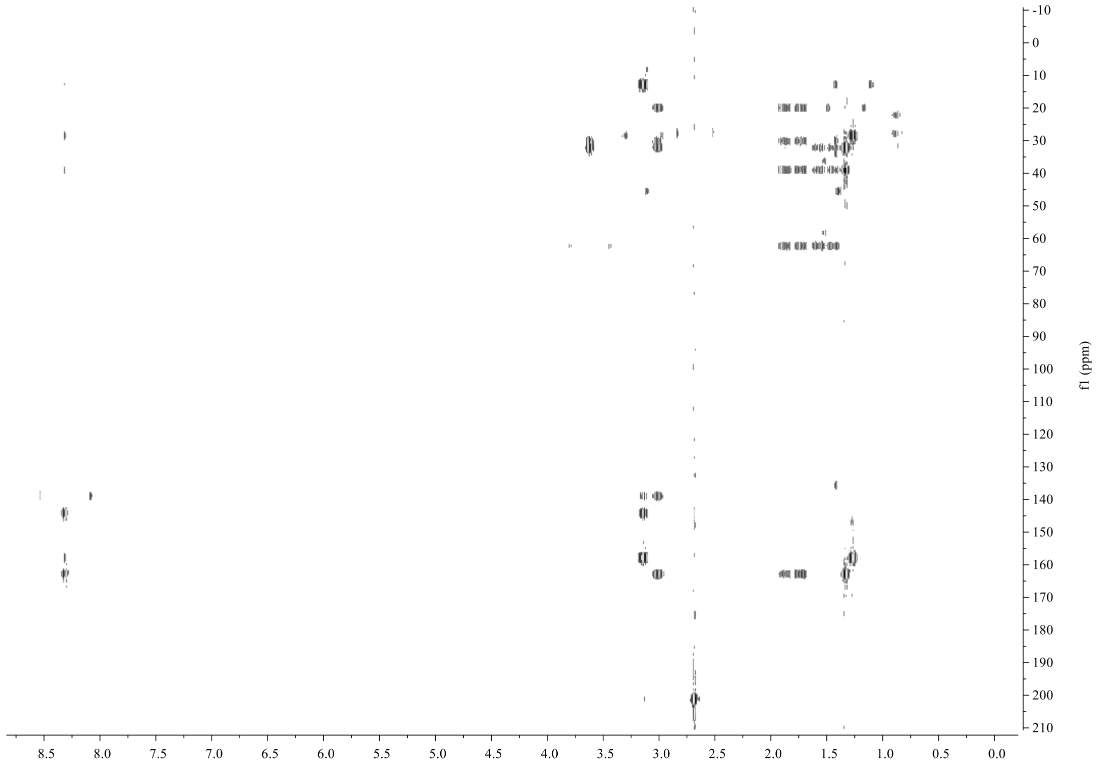

Supplementary Figure 45. HMBC spectra for compound 3s.

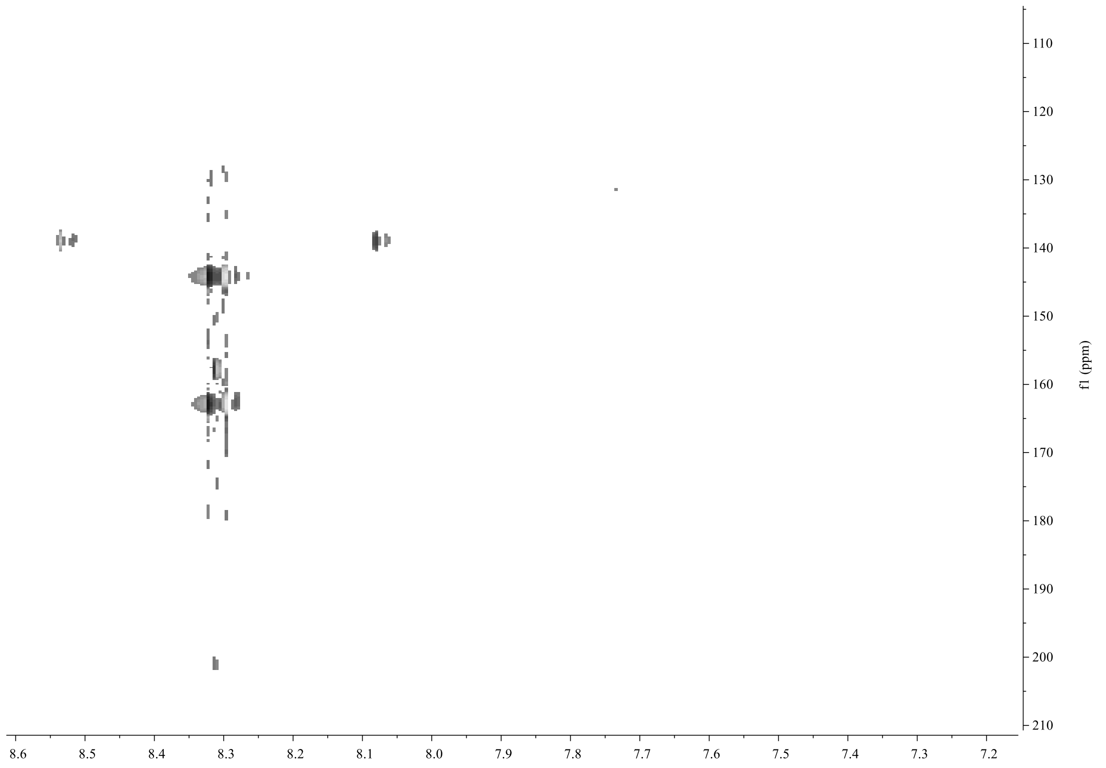

Supplementary Figure 46. Magnified HMBC spectra for compound 3s.

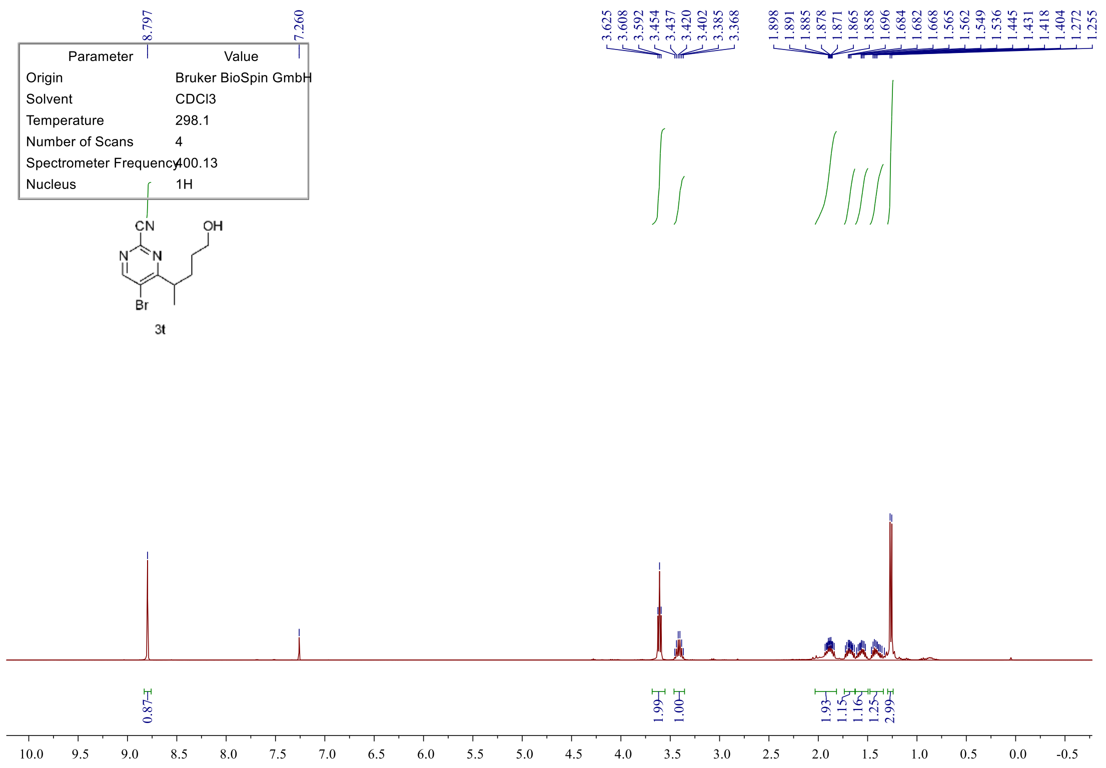

Supplementary Figure 47. ^{1}H NMR (400 MHz, $CDCl_3$) spectra for compound 3t.

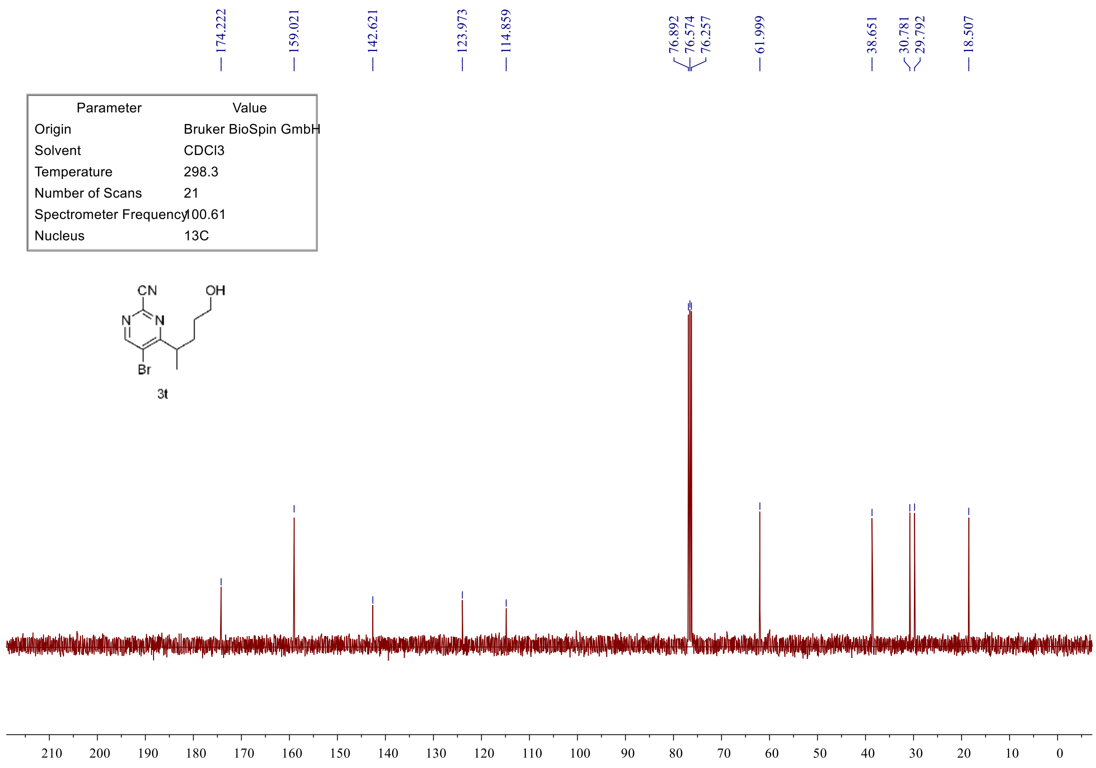

Supplementary Figure 48. ^{13}C NMR (100 MHz, $CDCl_3$) spectra for compound 3t.

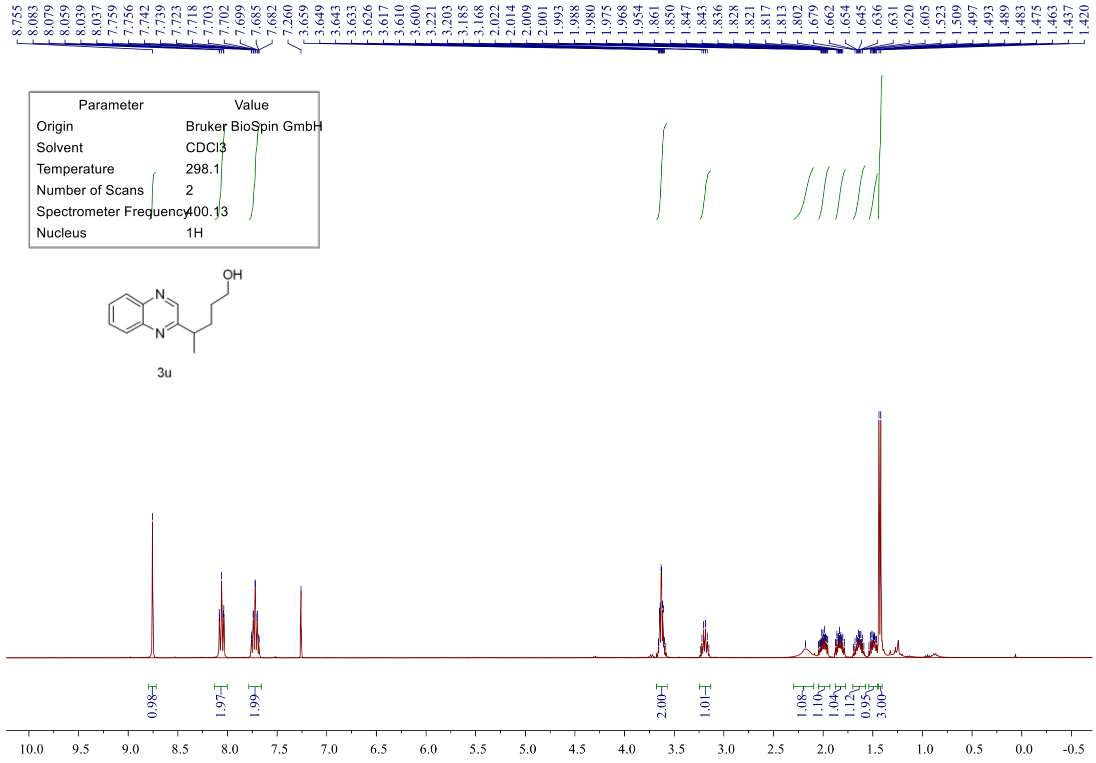

Supplementary Figure 49. ^{1}H NMR (400 MHz, $CDCl_3$) spectra for compound 3u.

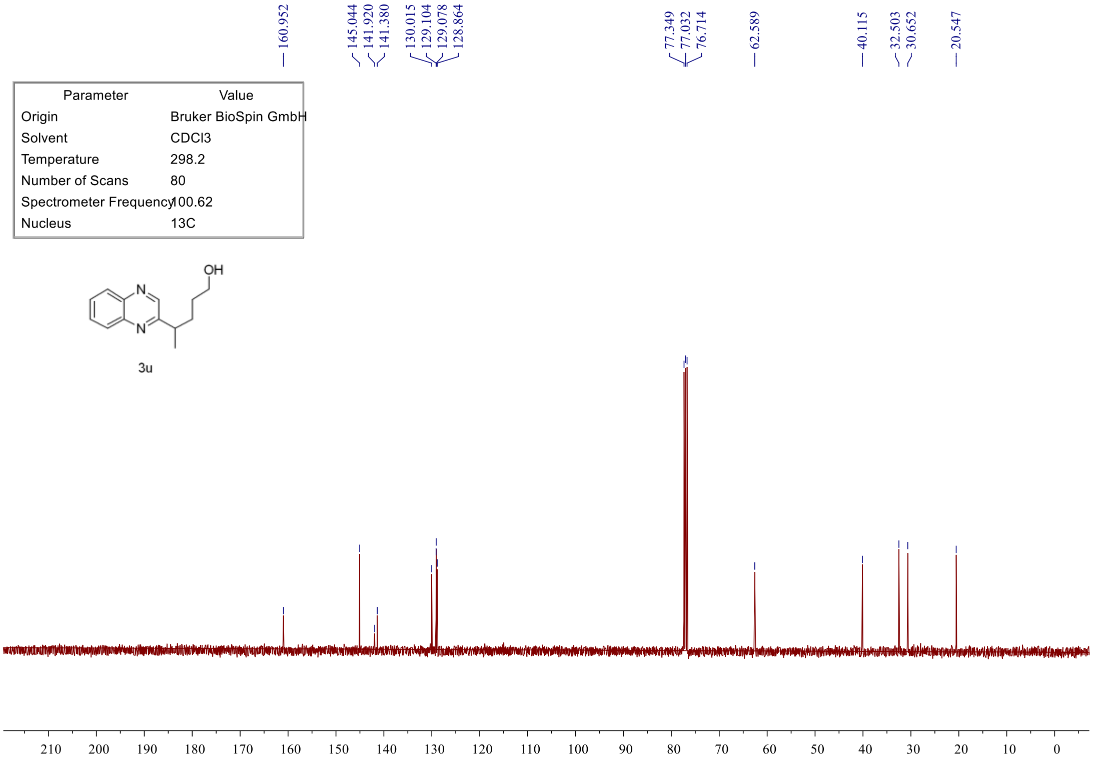

Supplementary Figure 50. ^{13}**C NMR (100 MHz, CDCl$_3$) spectra for compound 3u.**

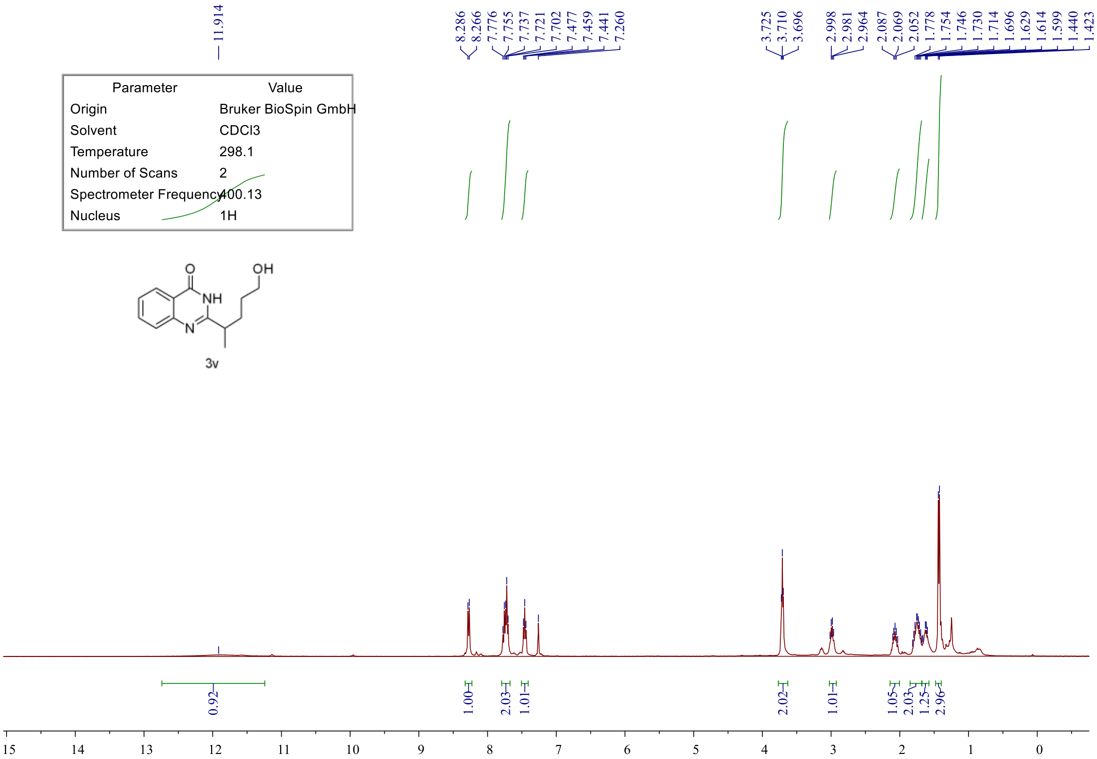

Supplementary Figure 51. ^{1}**H NMR (400 MHz, CDCl$_3$) spectra for compound 3v.**

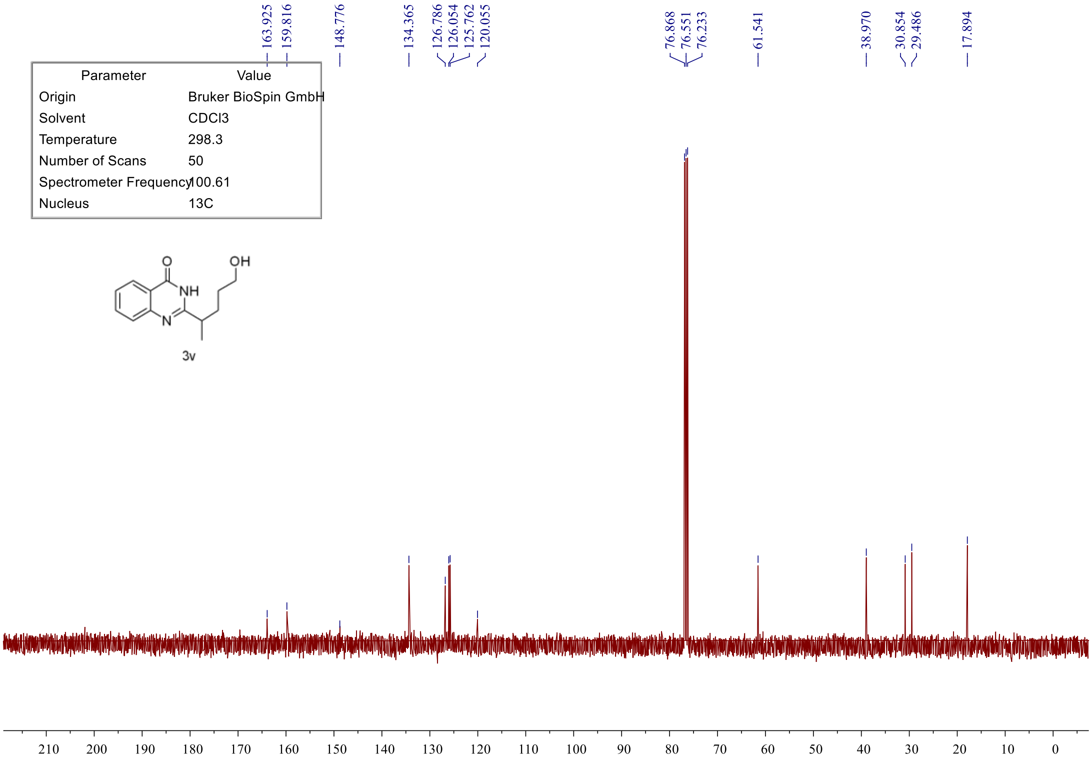

Supplementary Figure 52. ^{13}**C NMR (100 MHz, CDCl$_3$) spectra for compound 3v.**

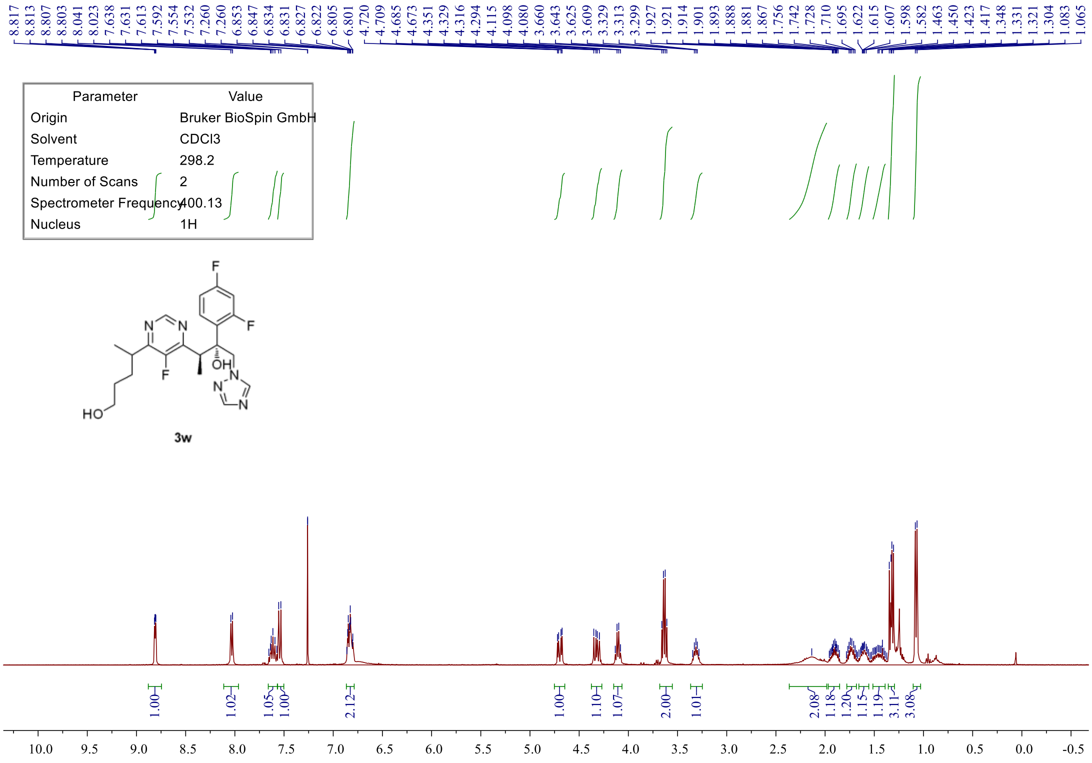

Supplementary Figure 53. ^{1}**H NMR (400 MHz, CDCl$_3$) spectra for compound 3w.**

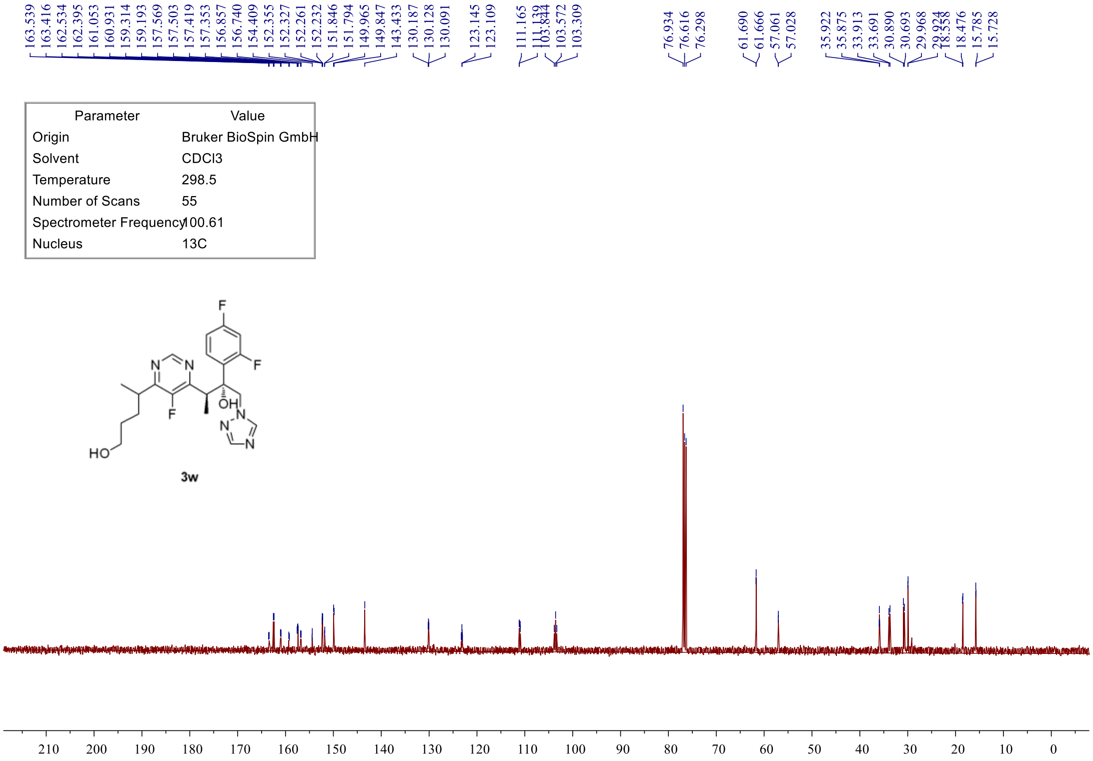

Supplementary Figure 54. ^{13}C **NMR (100 MHz,** $CDCl_3$**) spectra for compound 3w.**

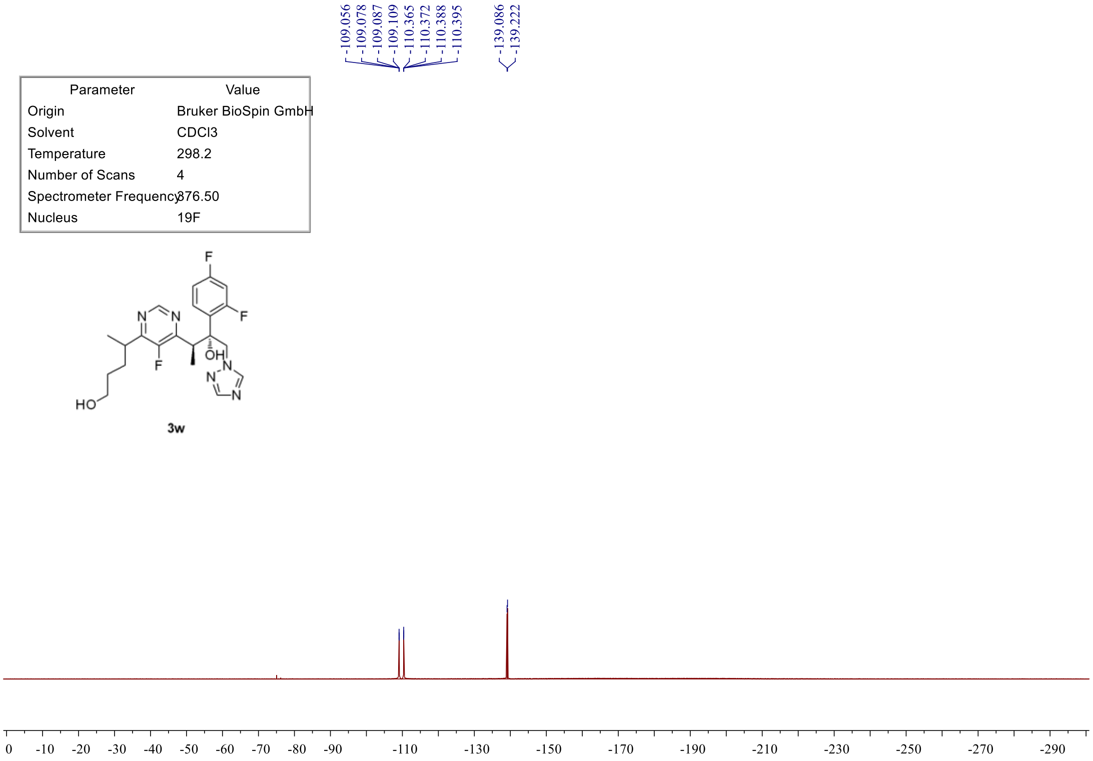

Supplementary Figure 55. ^{19}F **NMR (376 MHz,** $CDCl_3$**) spectra for compound 3w.**

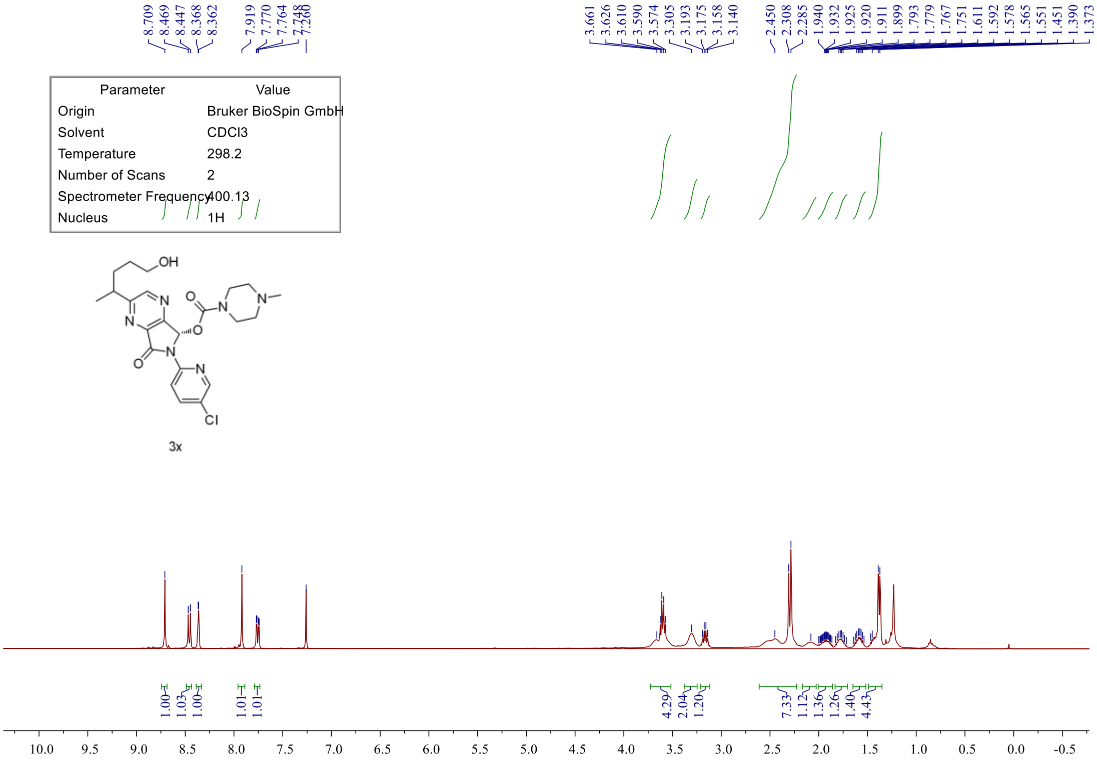

Supplementary Figure 56. ^{1}H NMR (400 MHz, $CDCl_3$) spectra for compound 3x.

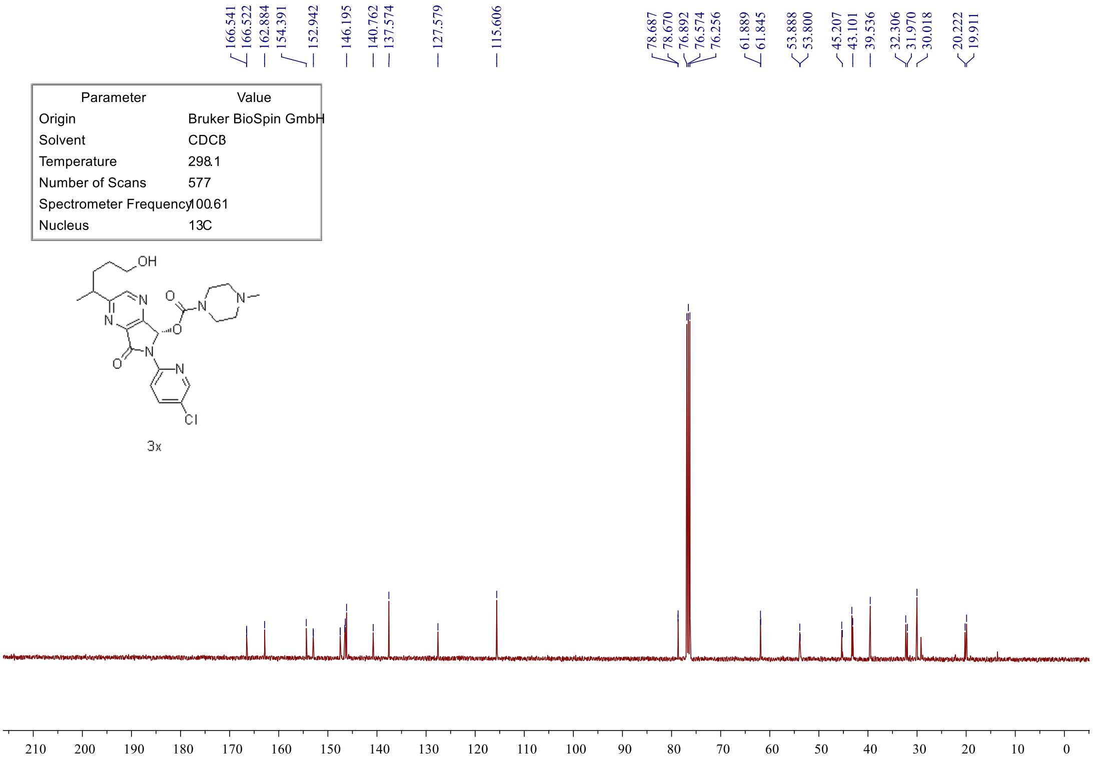

Supplementary Figure 57. ^{13}C NMR (100 MHz, $CDCl_3$) spectra for compound 3x.

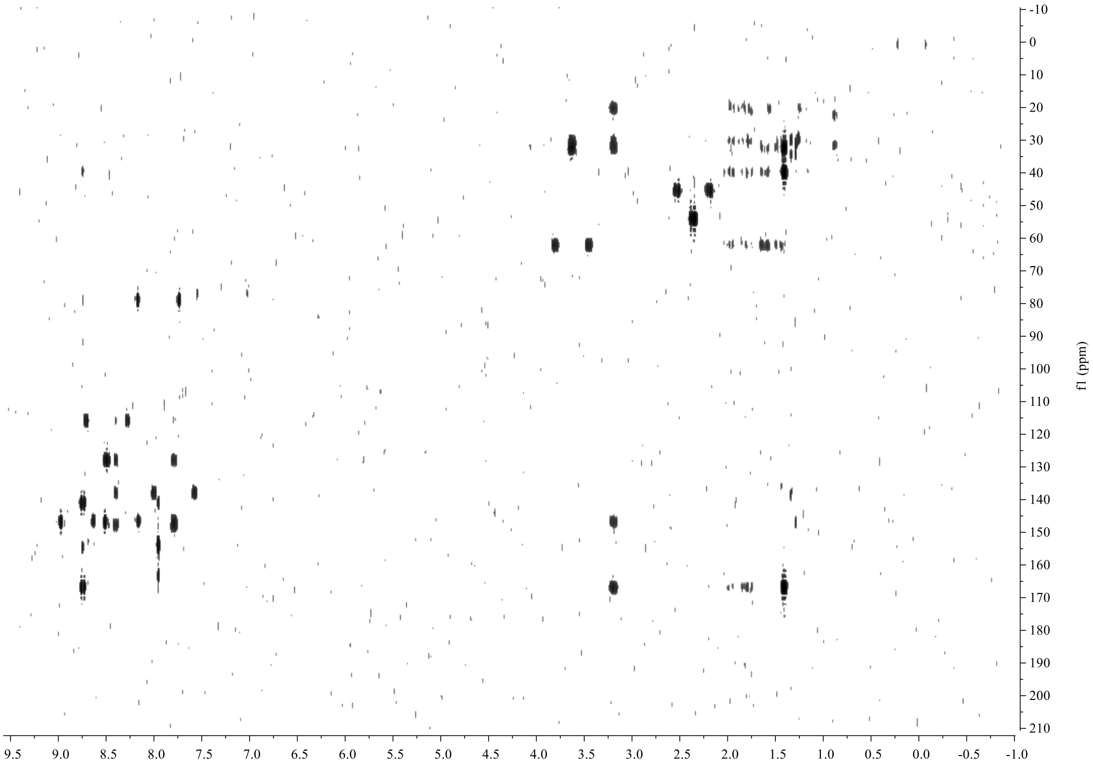

Supplementary Figure 58. HMBC spectra for compound 3x.

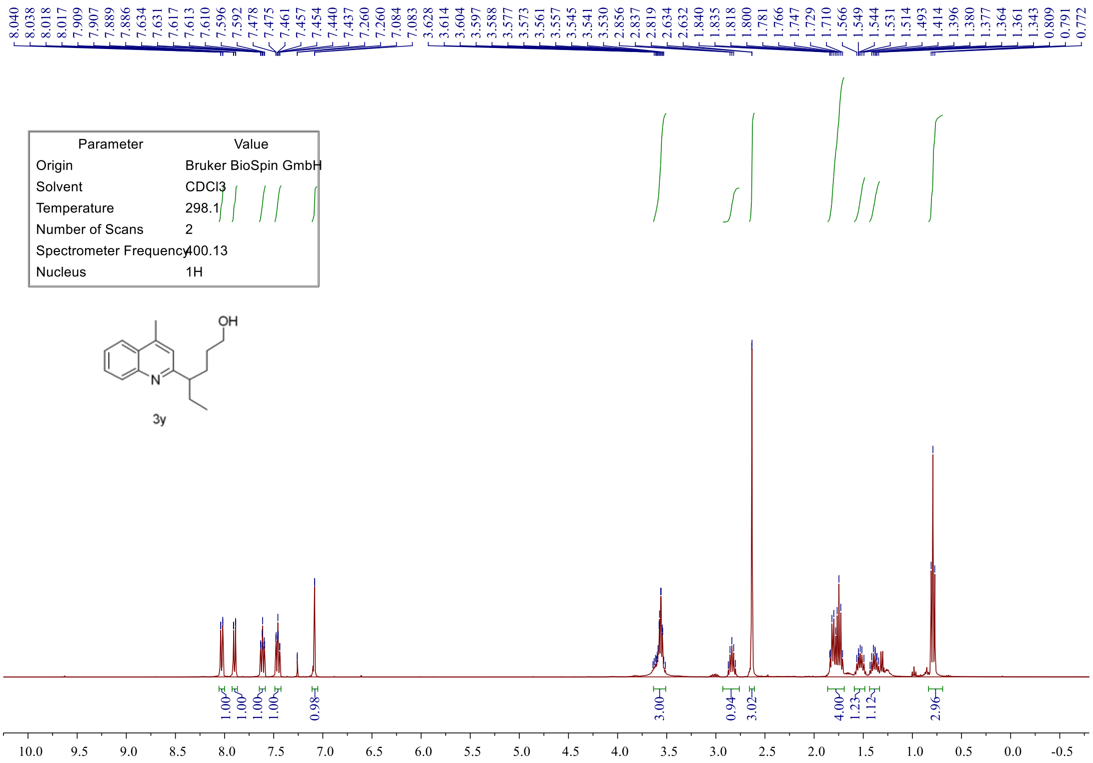

Supplementary Figure 59. ^{1}H NMR (400 MHz, $CDCl_3$) spectra for compound 3y.

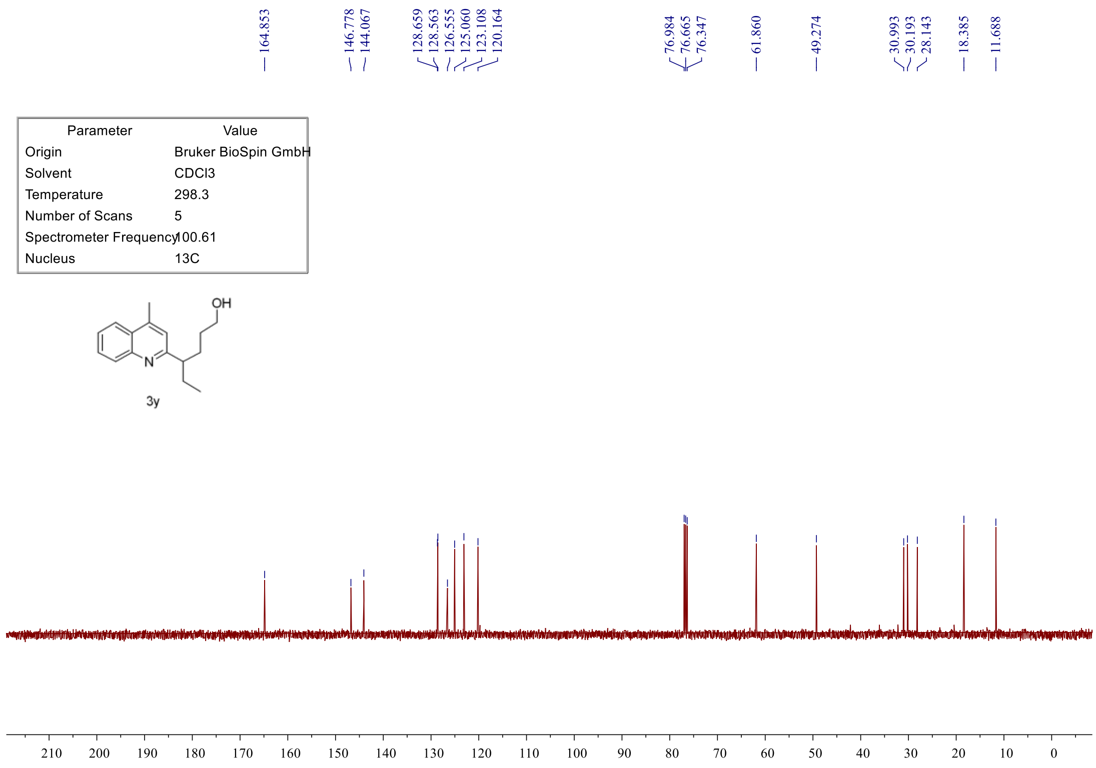

Supplementary Figure 60. ^{13}C NMR (100 MHz, $CDCl_3$) spectra for compound 3y.

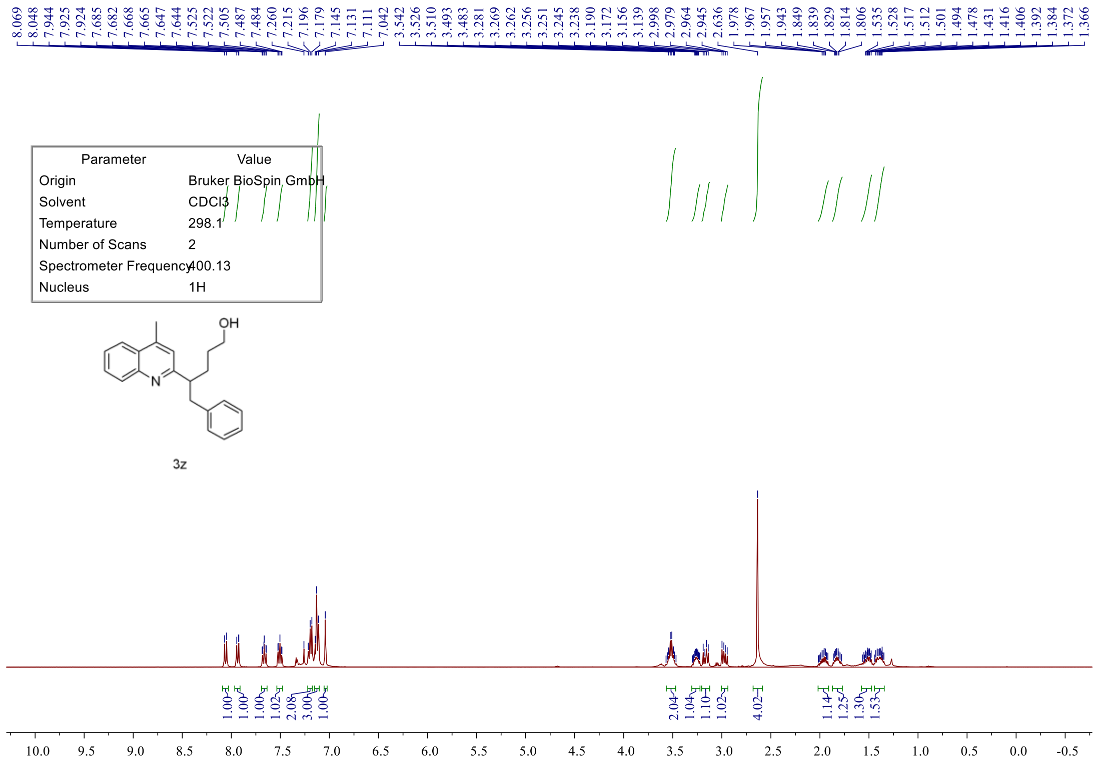

Supplementary Figure 61. ^{1}H NMR (400 MHz, $CDCl_3$) spectra for compound 3z.

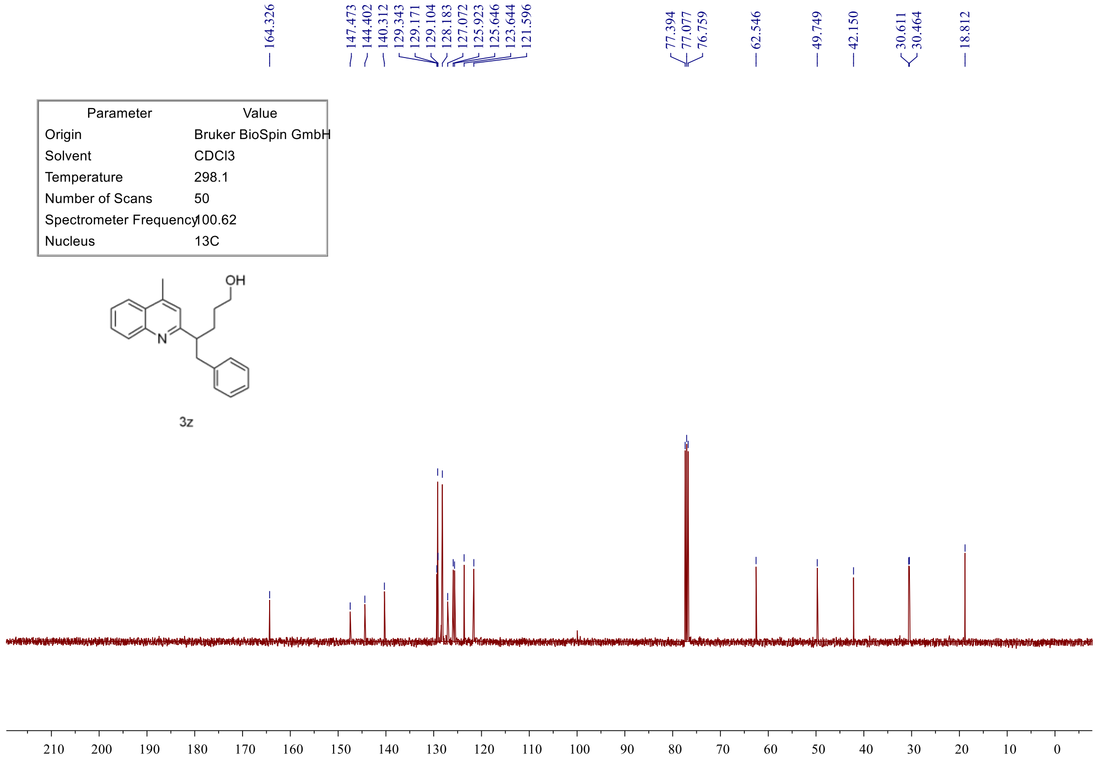

Supplementary Figure 62. ^{13}C NMR (100 MHz, $CDCl_3$) spectra for compound 3z.

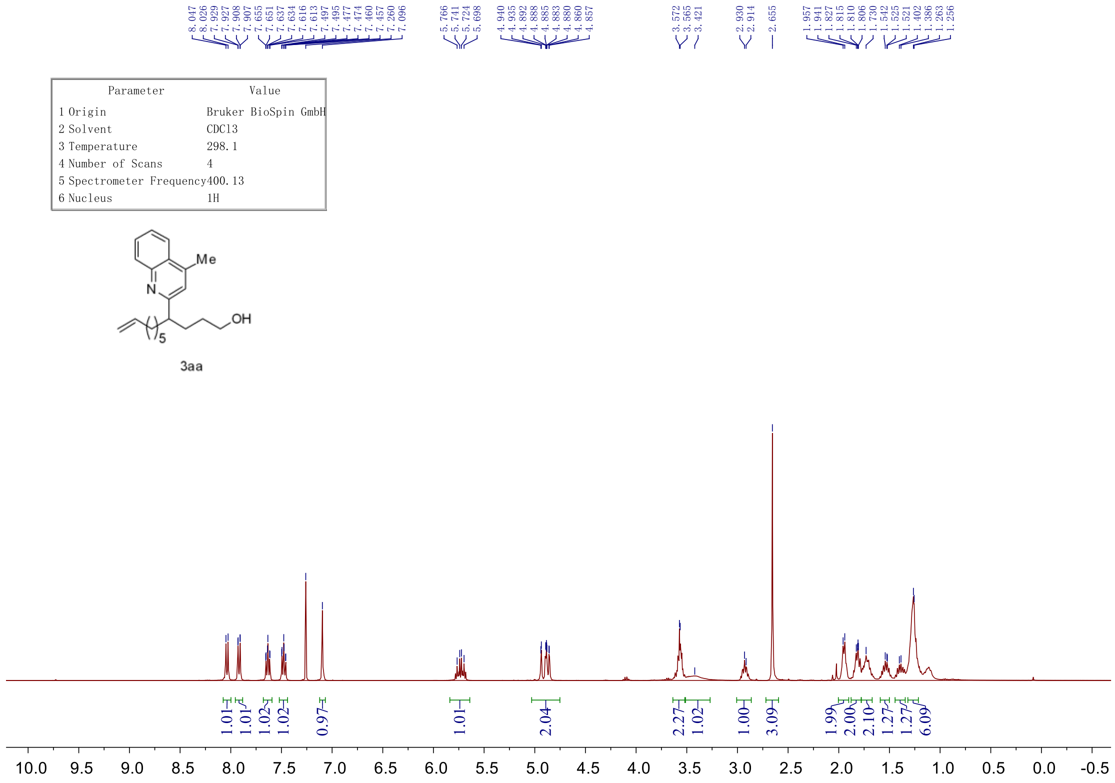

Supplementary Figure 63. ^{1}H NMR (400 MHz, $CDCl_3$) spectra for compound 3aa.

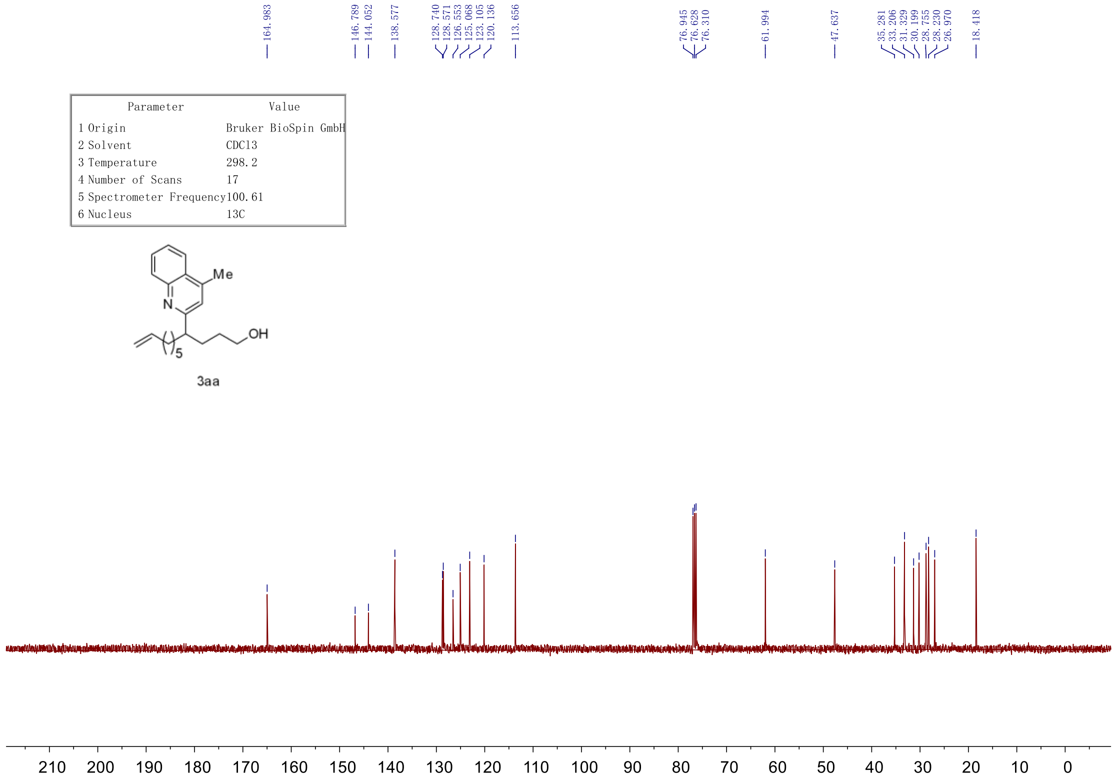

Supplementary Figure 64. ^{13}C NMR (100 MHz, $CDCl_3$) spectra for compound 3aa.

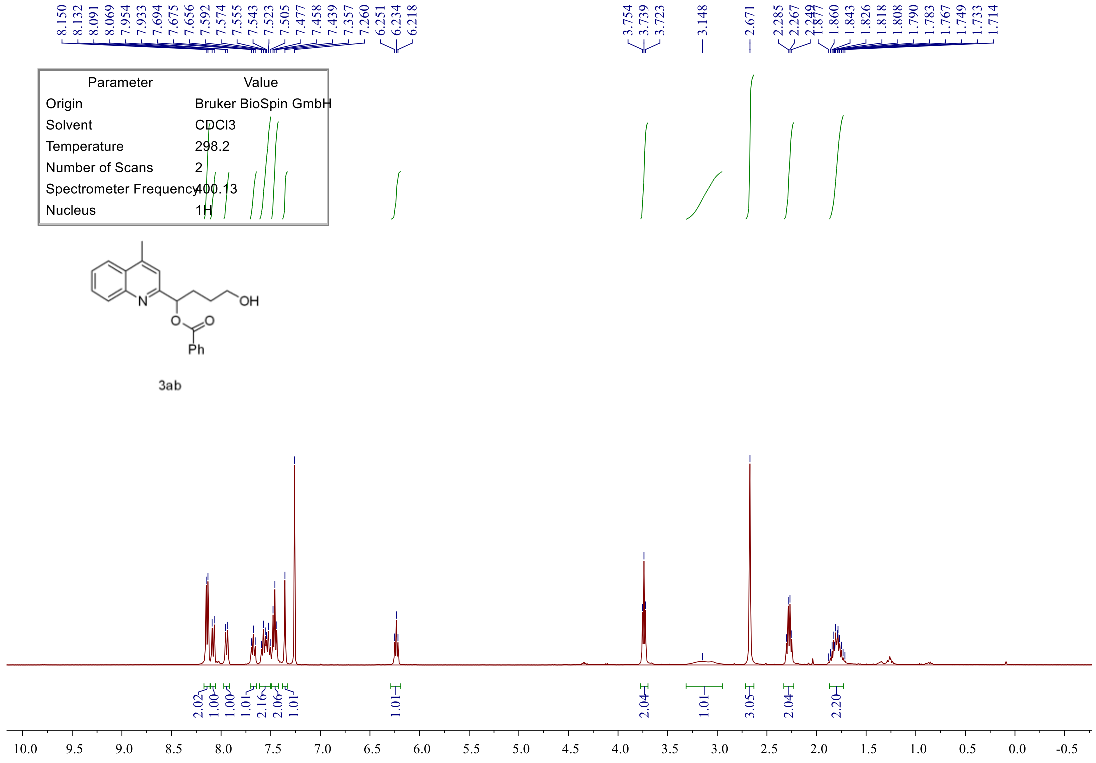

Supplementary Figure 65. ^{1}H NMR (400 MHz, $CDCl_3$) spectra for compound 3ab.

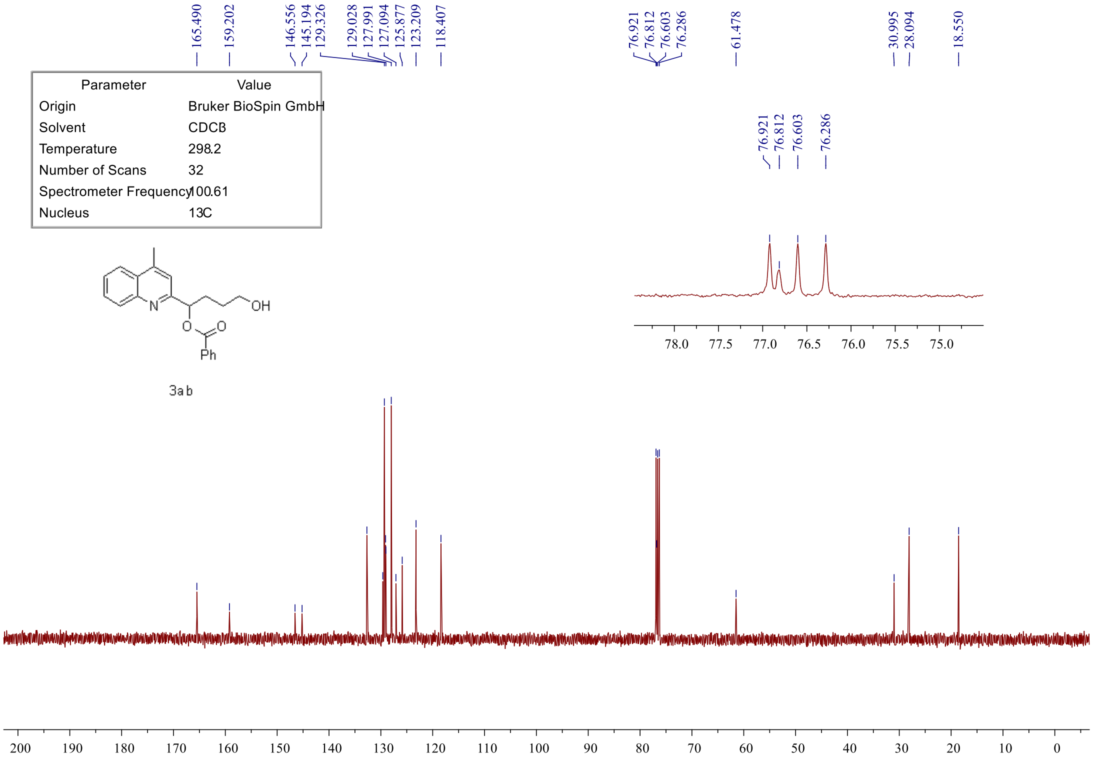

Supplementary Figure 66. ^{13}C NMR (100 MHz, $CDCl_3$) spectra for compound 3ab.

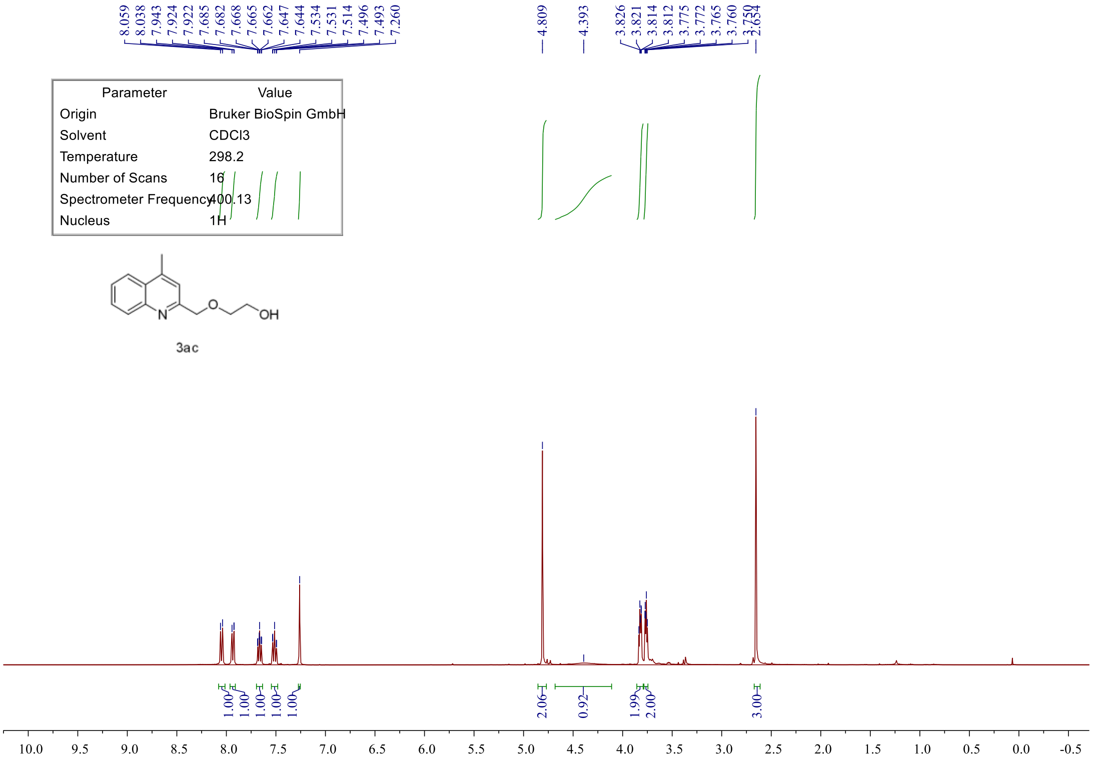

Supplementary Figure 67. ^{1}H NMR (400 MHz, $CDCl_3$) spectra for compound 3ac.

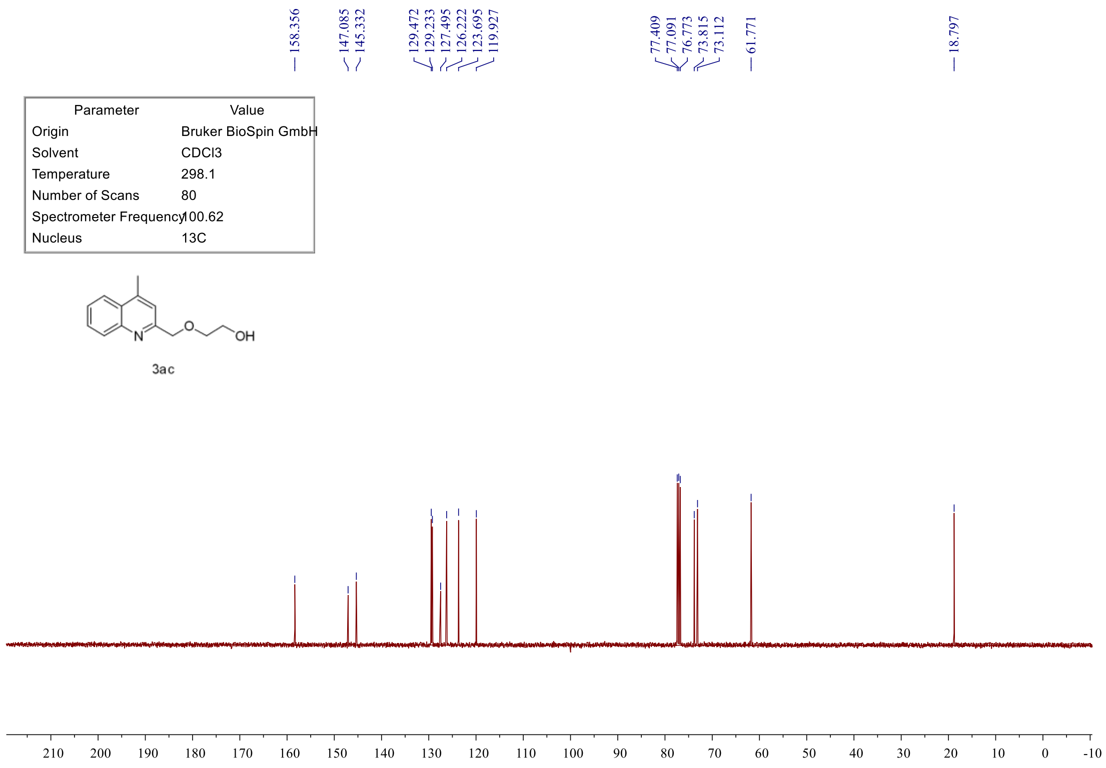

Supplementary Figure 68. ^{13}C NMR (100 MHz, $CDCl_3$) spectra for compound 3ac.

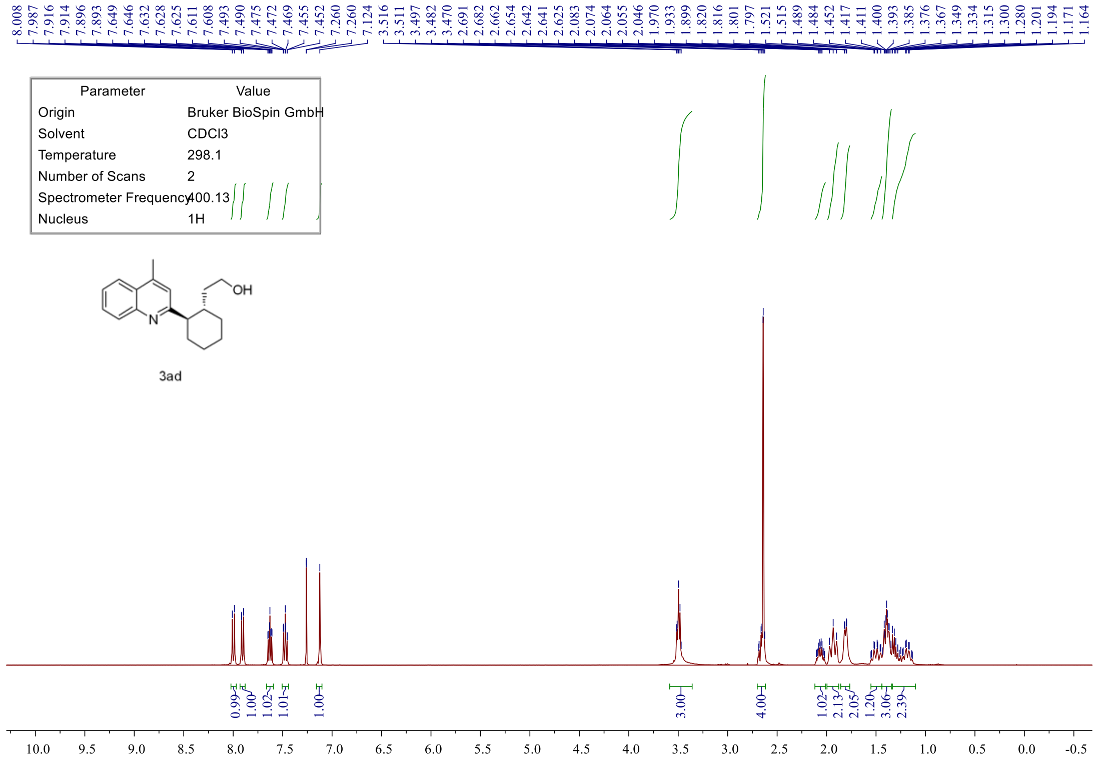

Supplementary Figure 69. ^{1}H NMR (400 MHz, $CDCl_3$) spectra for compound 3ad.

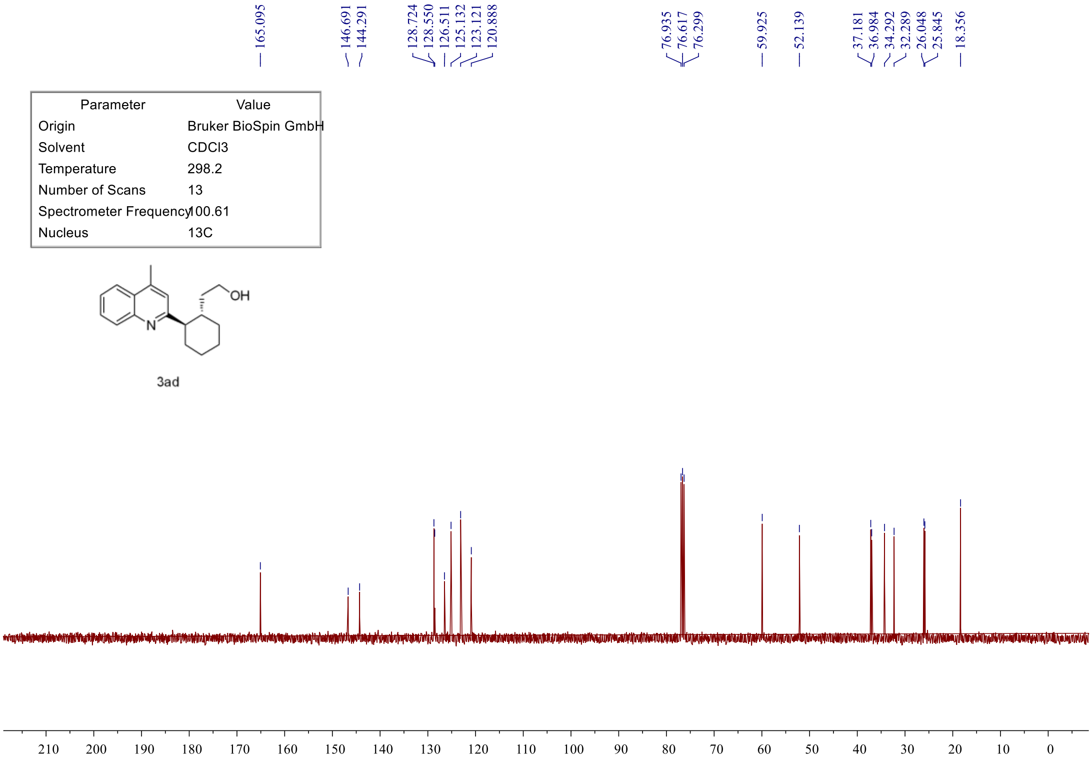

Supplementary Figure 70. ^{13}C NMR (100 MHz, $CDCl_3$) spectra for compound 3ad.

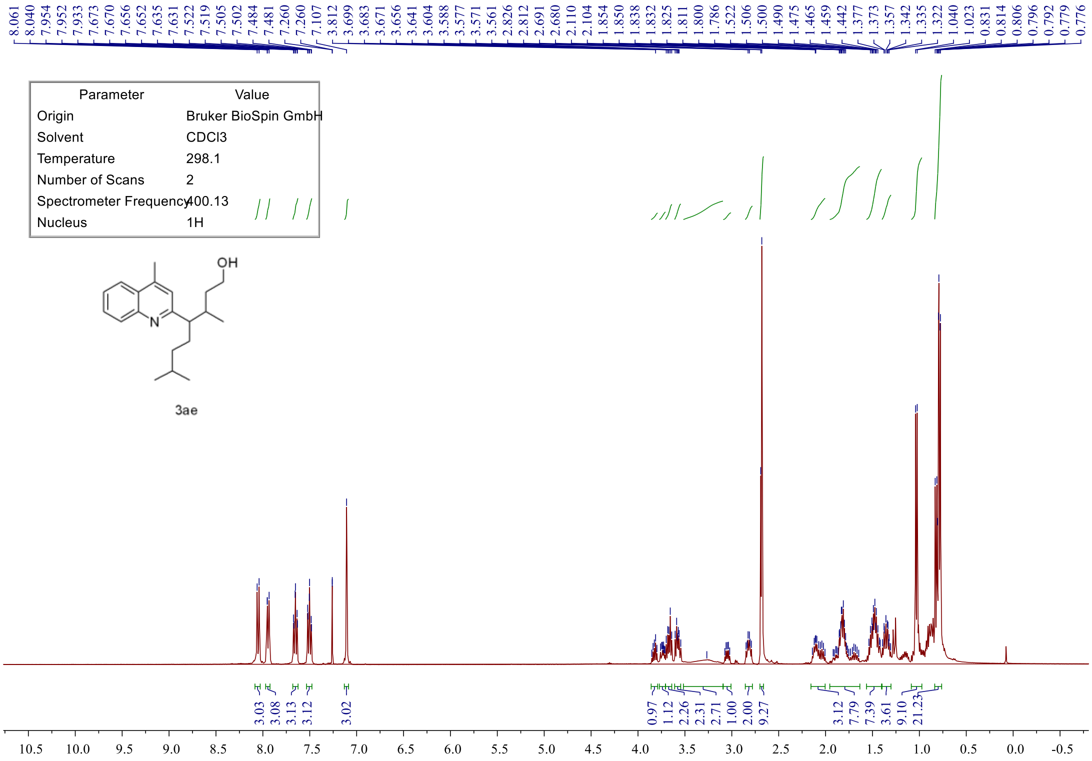

Supplementary Figure 71. ^{1}H NMR (400 MHz, $CDCl_3$) spectra for compound 3ae.

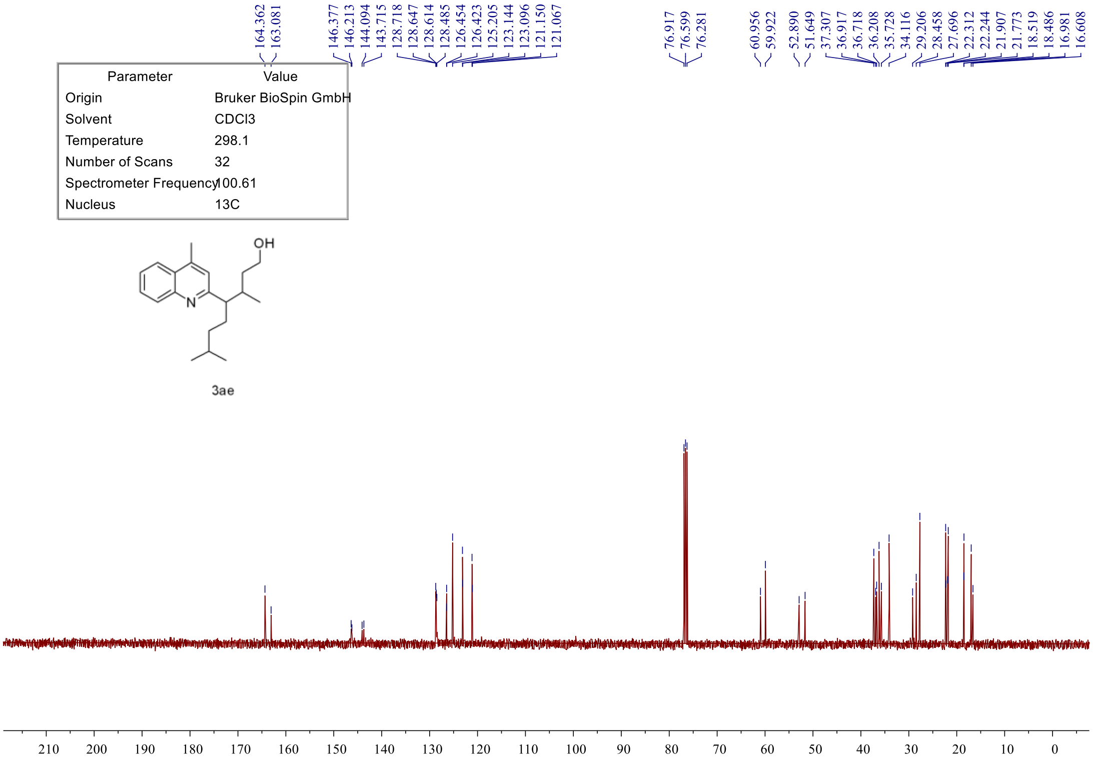

Supplementary Figure 72. ^{13}C NMR (100 MHz, $CDCl_3$) spectra for compound 3ae.

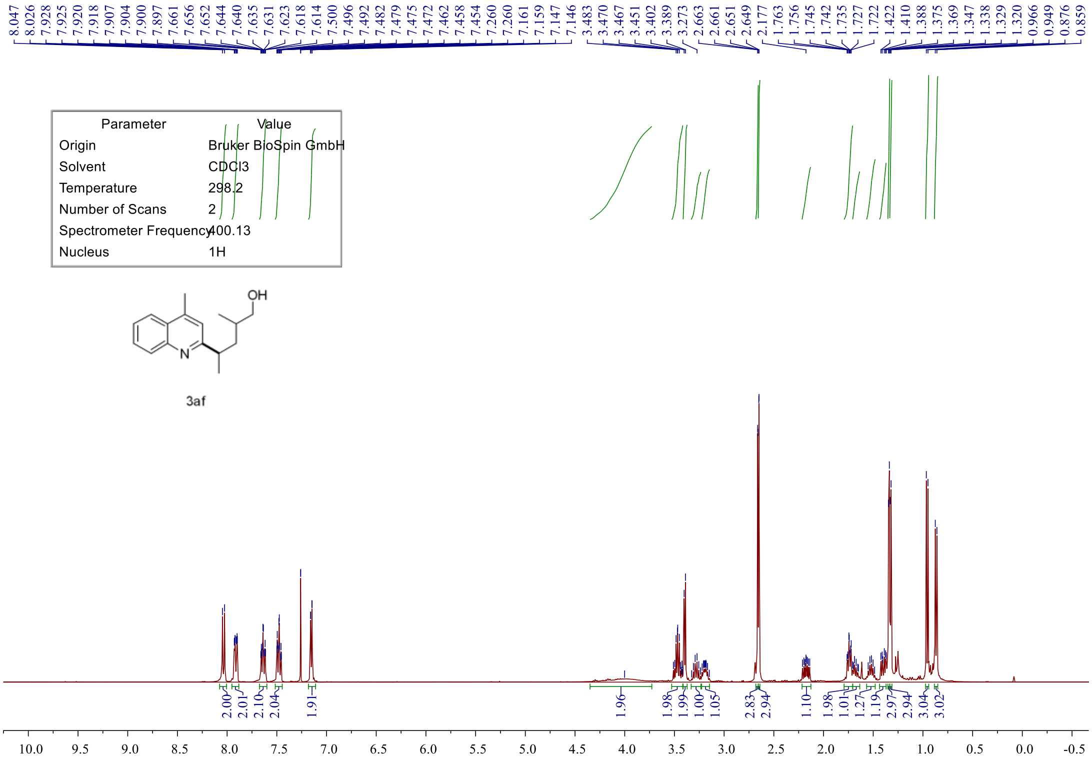

Supplementary Figure 73. ^{1}H NMR (400 MHz, $CDCl_3$) spectra for compound 3af.

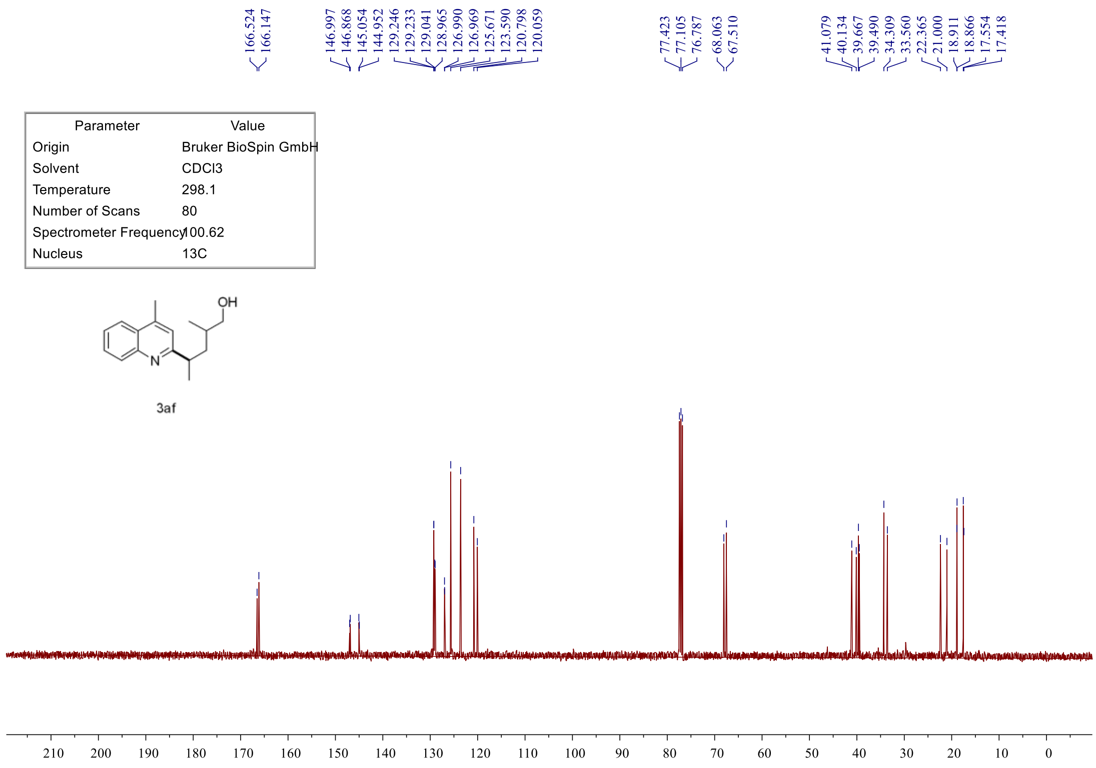

Supplementary Figure 74. ^{13}**C NMR (100 MHz, CDCl**$_3$**) spectra for compound 3af.**

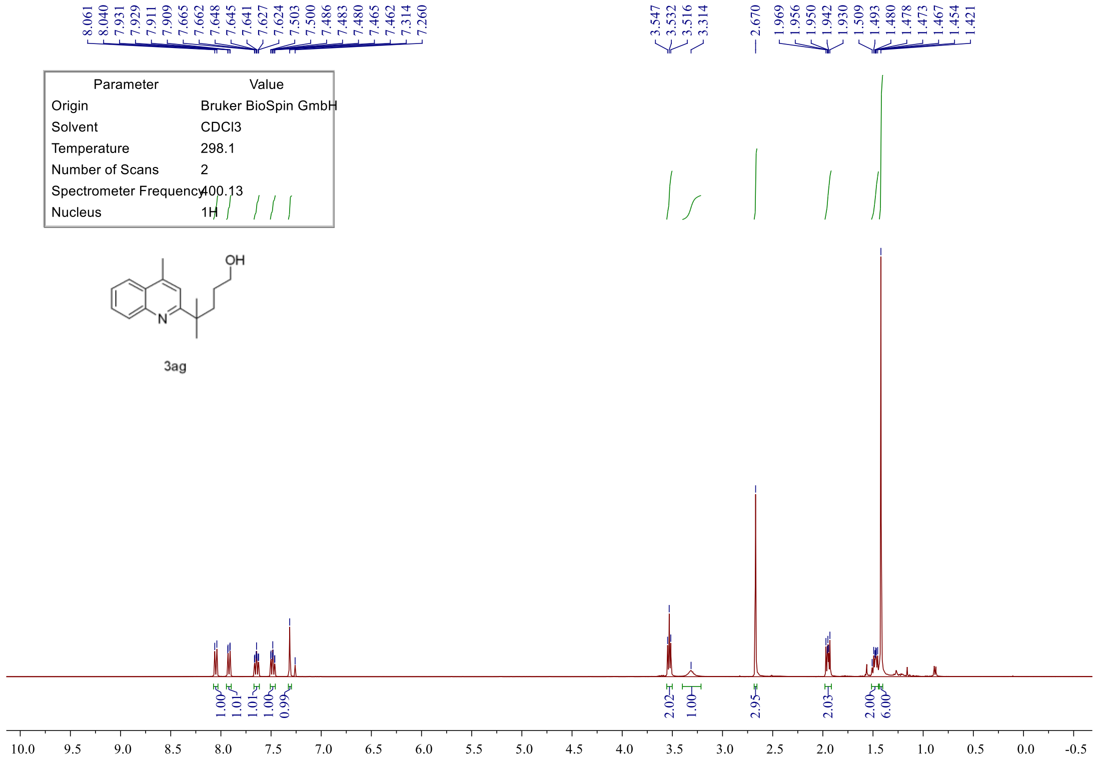

Supplementary Figure 75. ^{1}**H NMR (400 MHz, CDCl**$_3$**) spectra for compound 3ag.**

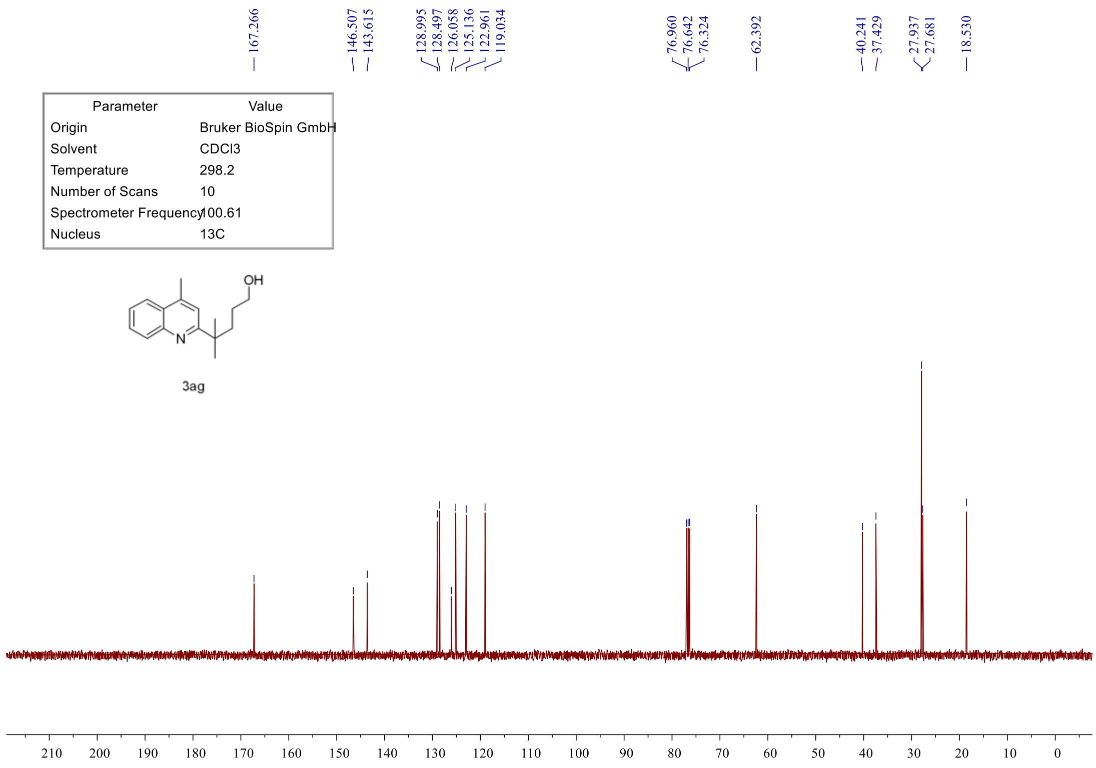

Supplementary Figure 76. ^{13}C NMR (100 MHz, $CDCl_3$) spectra for compound 3ag.

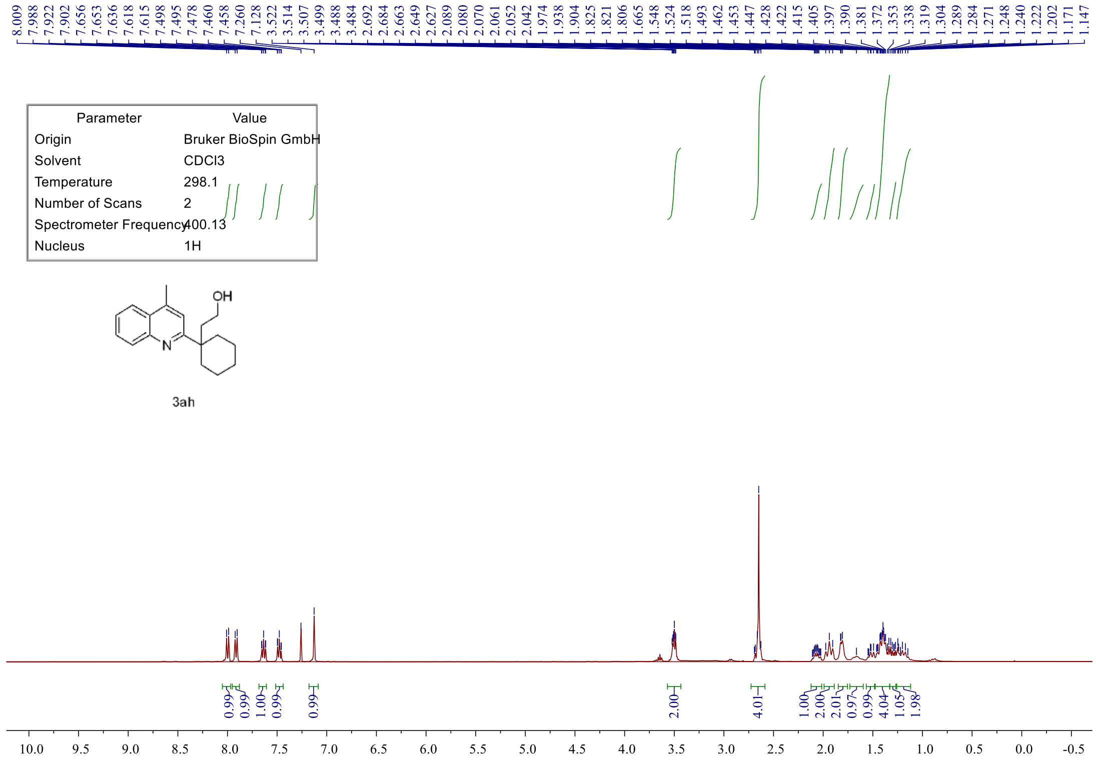

Supplementary Figure 77. ^{1}H NMR (400 MHz, $CDCl_3$) spectra for compound 3ah.

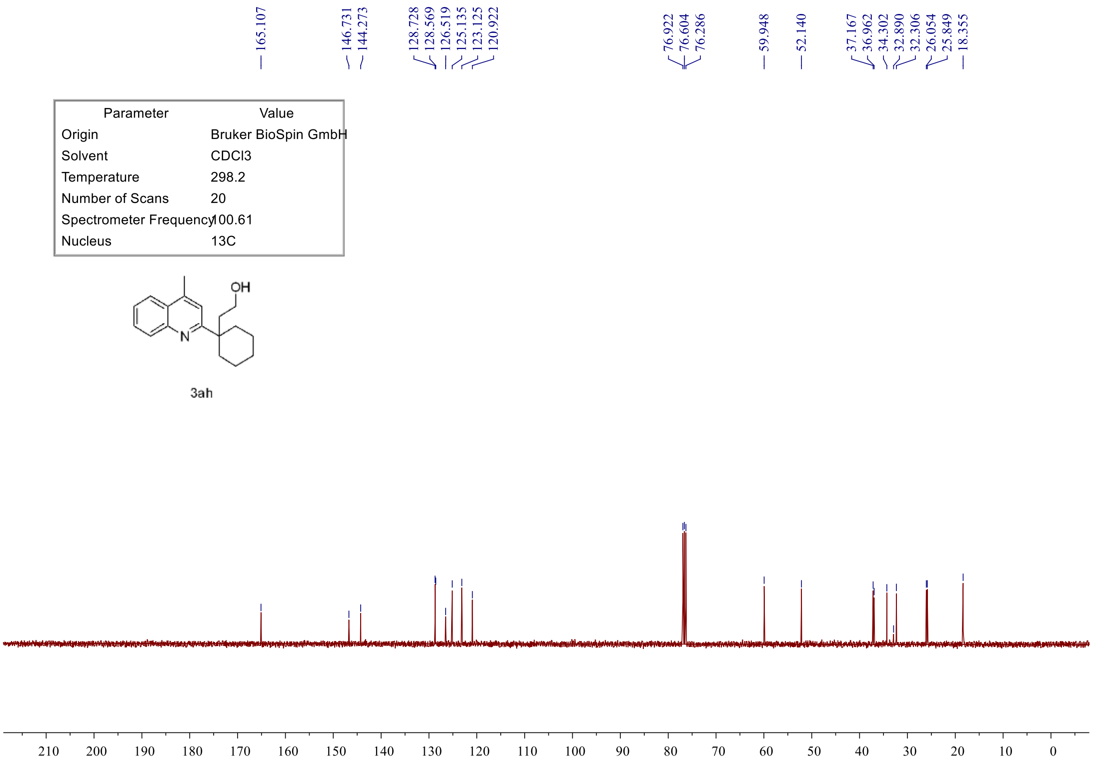

Supplementary Figure 78. ^{13}C NMR (100 MHz, $CDCl_3$) spectra for compound 3ah.

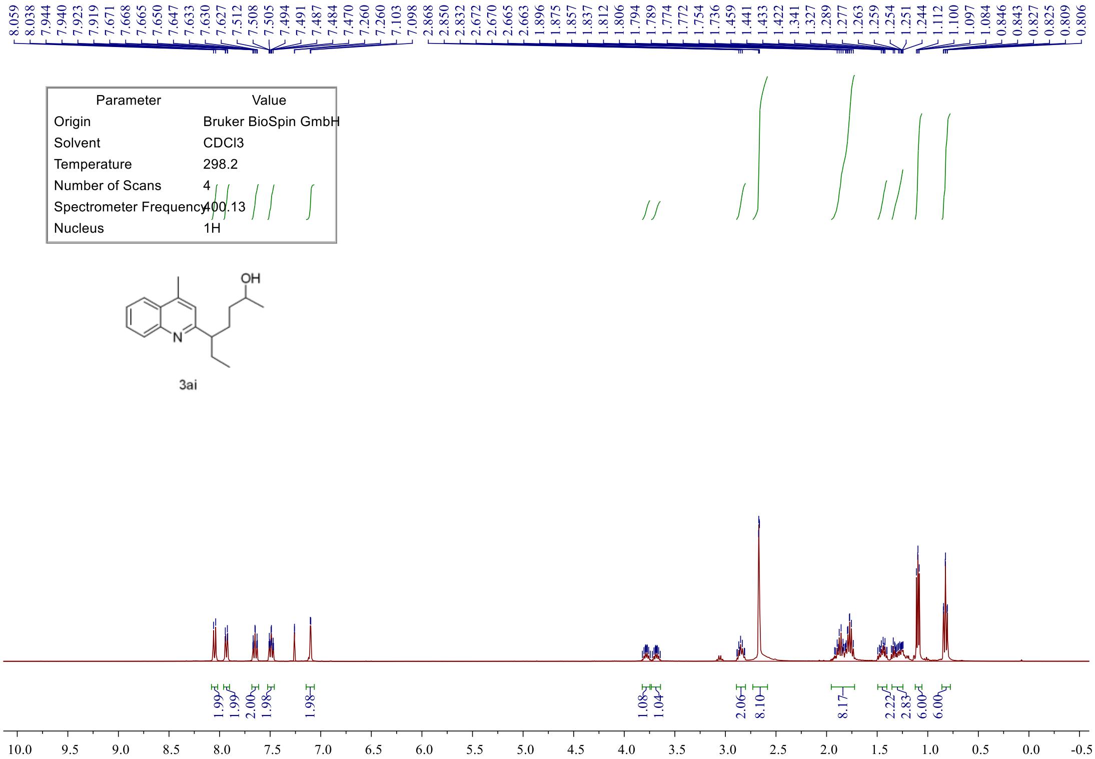

Supplementary Figure 79. ^{1}H NMR (400 MHz, $CDCl_3$) spectra for compound 3ai.

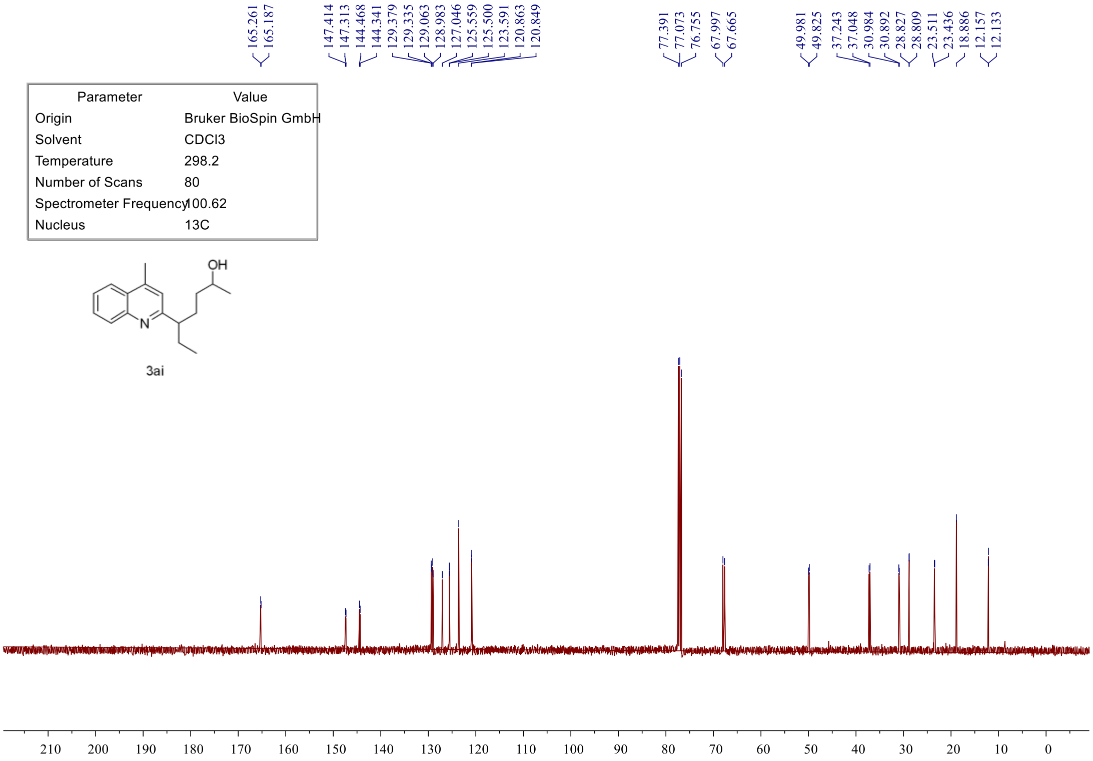

Supplementary Figure 80. ^{13}**C NMR (100 MHz, CDCl$_3$) spectra for compound 3ai.**

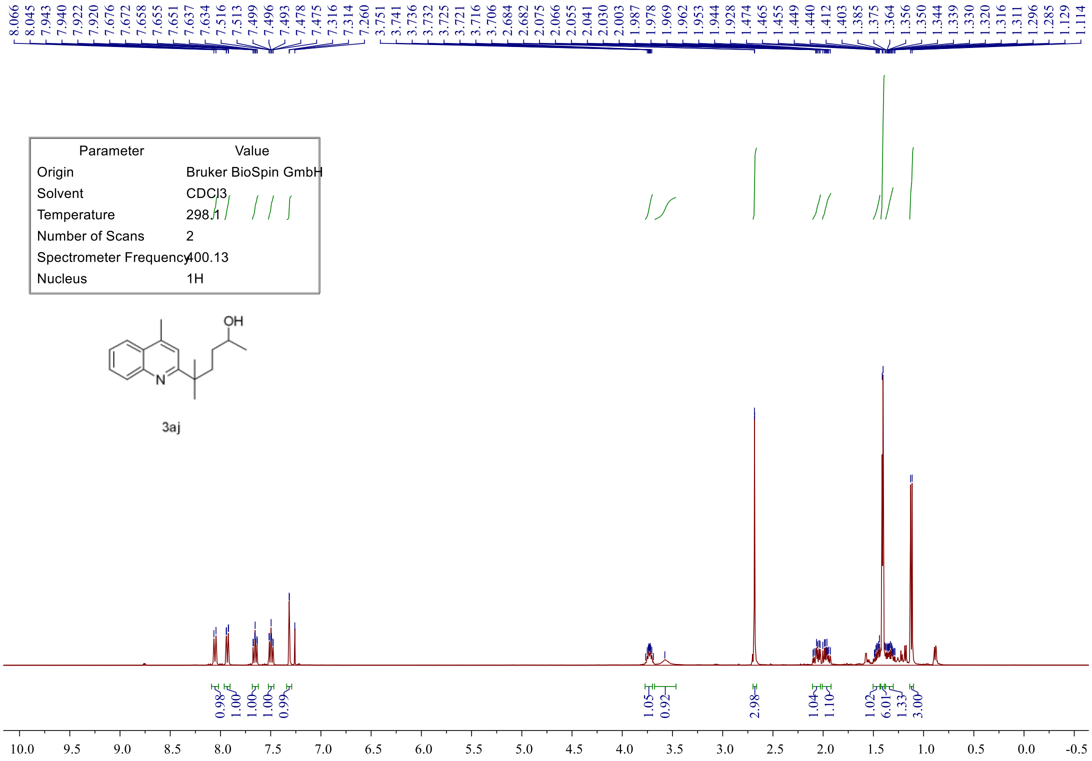

Supplementary Figure 81. ^{1}**H NMR (400 MHz, CDCl$_3$) spectra for compound 3aj.**

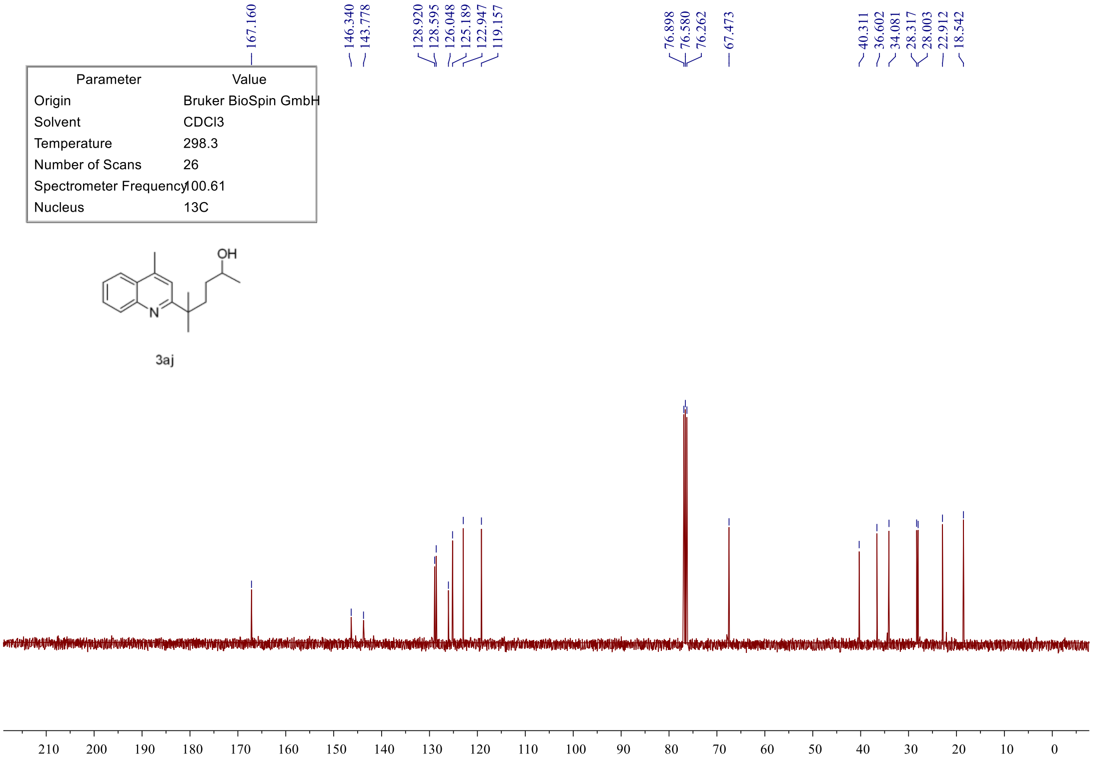

Supplementary Figure 82. ^{13}**C NMR (100 MHz,** $CDCl_3$**) spectra for compound 3aj.**

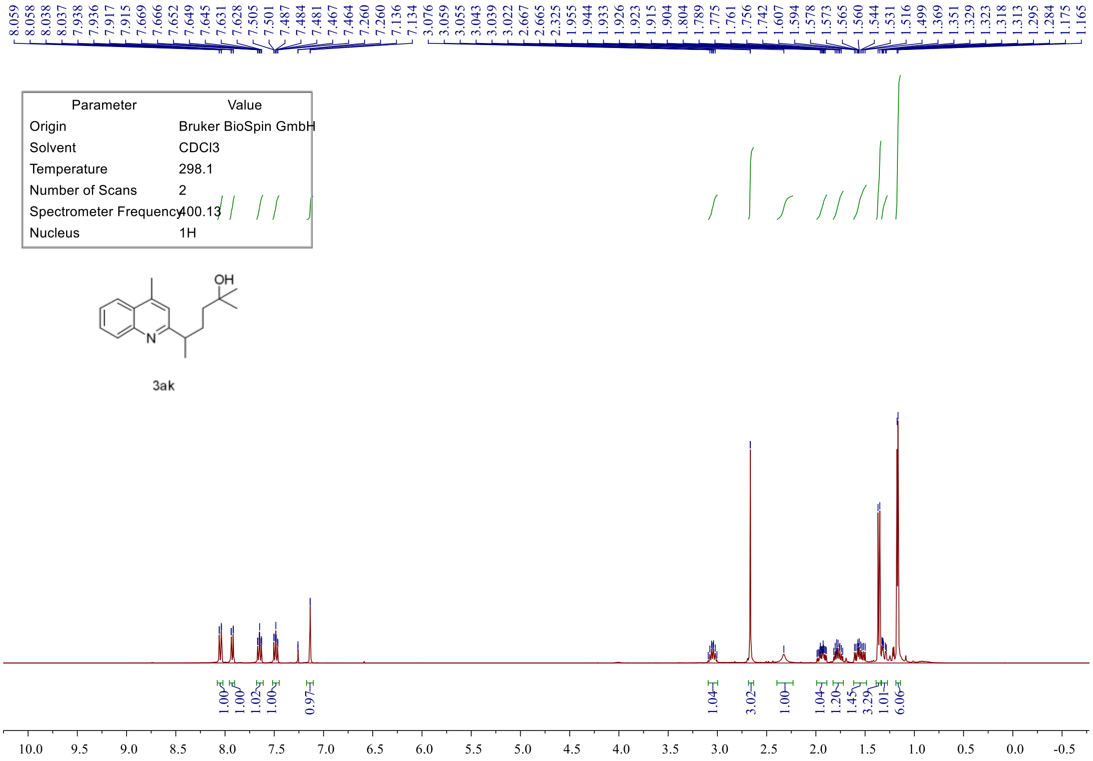

Supplementary Figure 83. ^{1}**H NMR (400 MHz,** $CDCl_3$**) spectra for compound 3ak.**

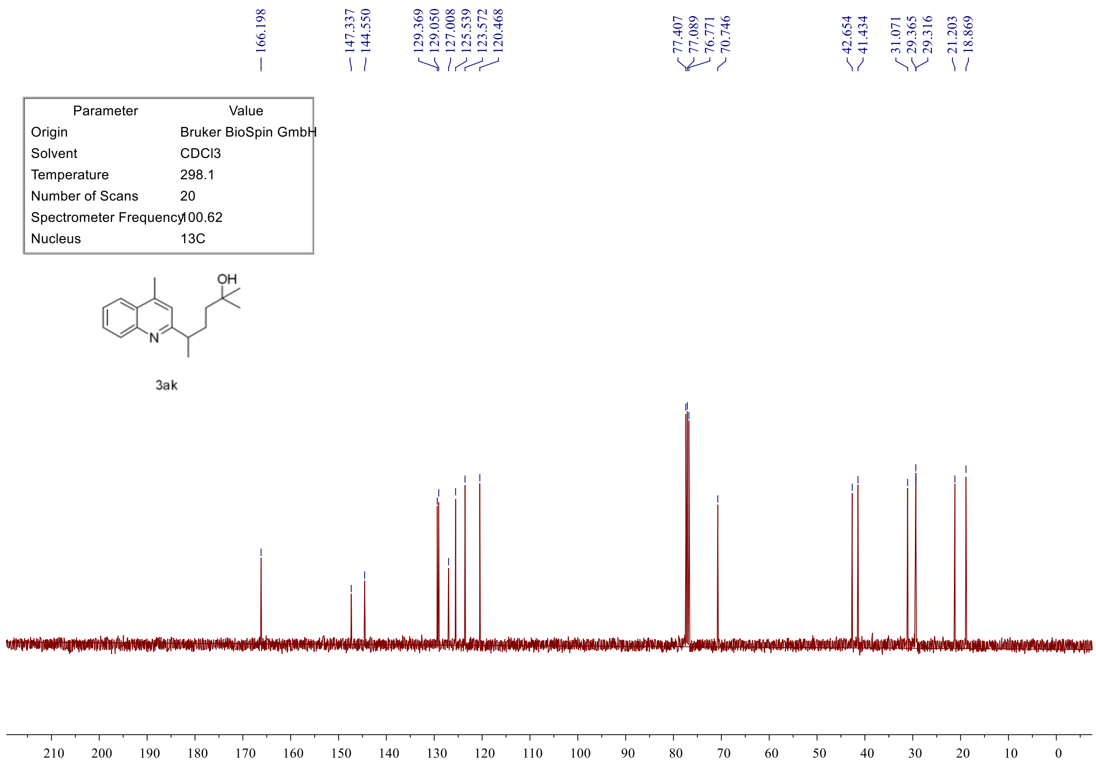

Supplementary Figure 84. ^{13}C NMR (100 MHz, $CDCl_3$) spectra for compound 3ak.

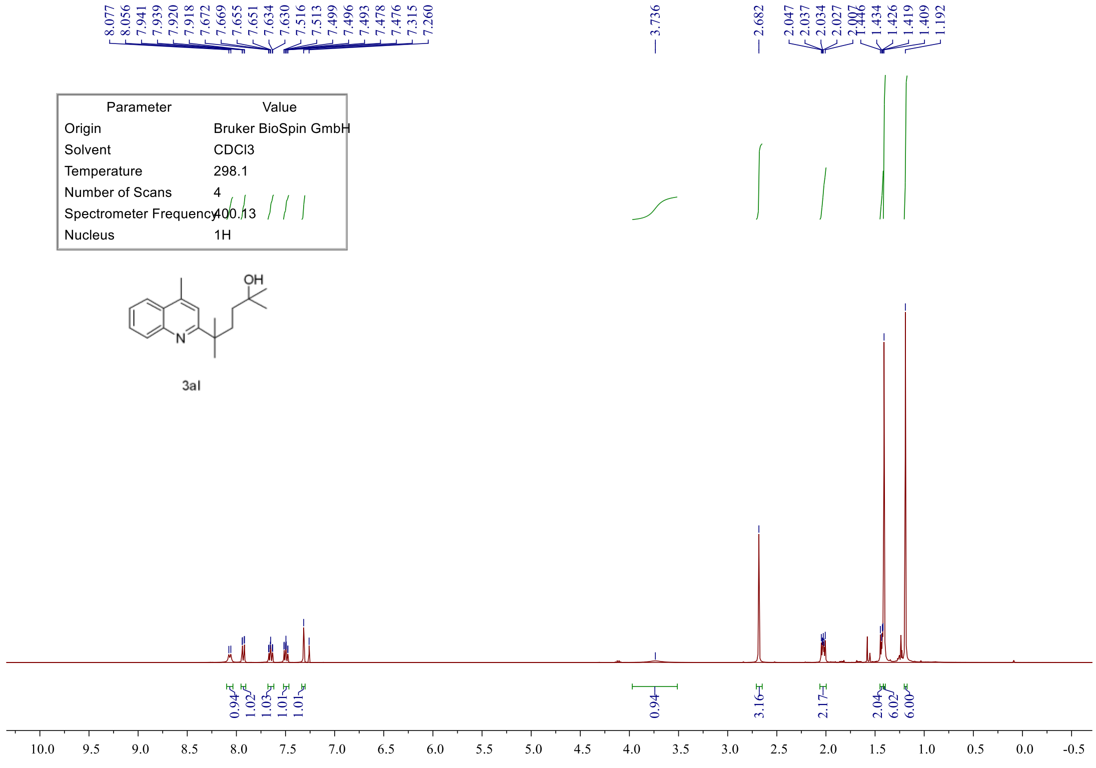

Supplementary Figure 85. ^{1}H NMR (400 MHz, $CDCl_3$) spectra for compound 3al.

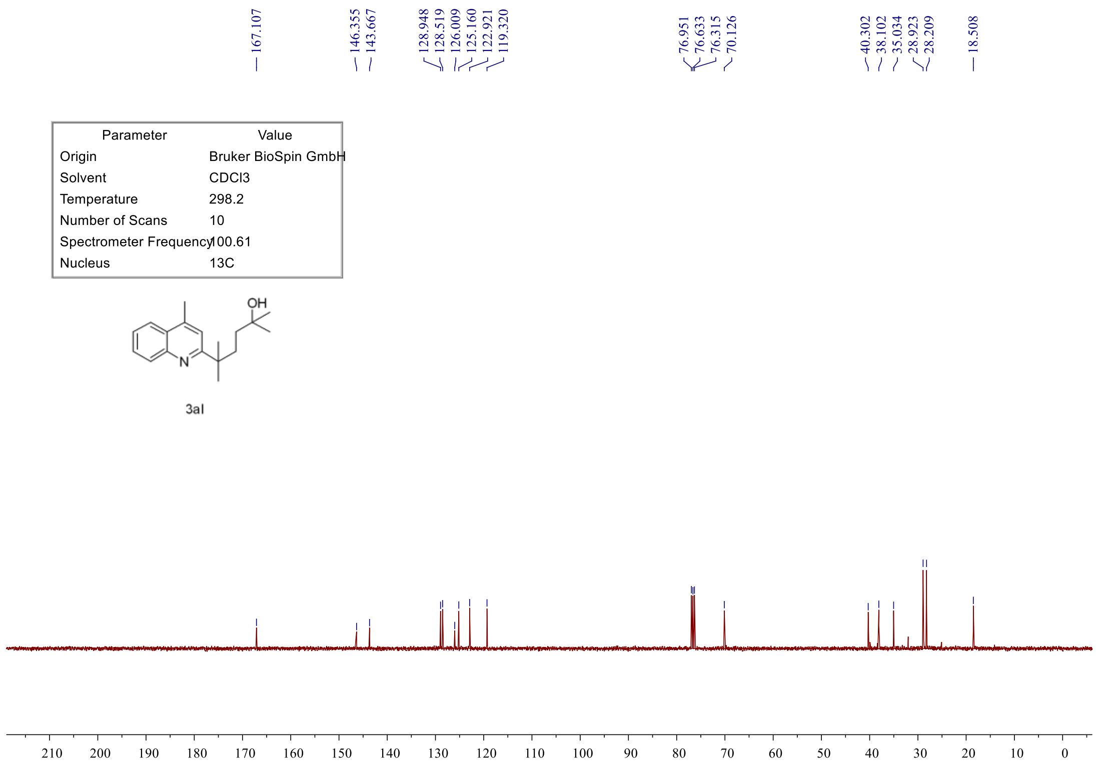

Supplementary Figure 86. ^{13}C **NMR (100 MHz,** $CDCl_3$**) spectra for compound 3al.**

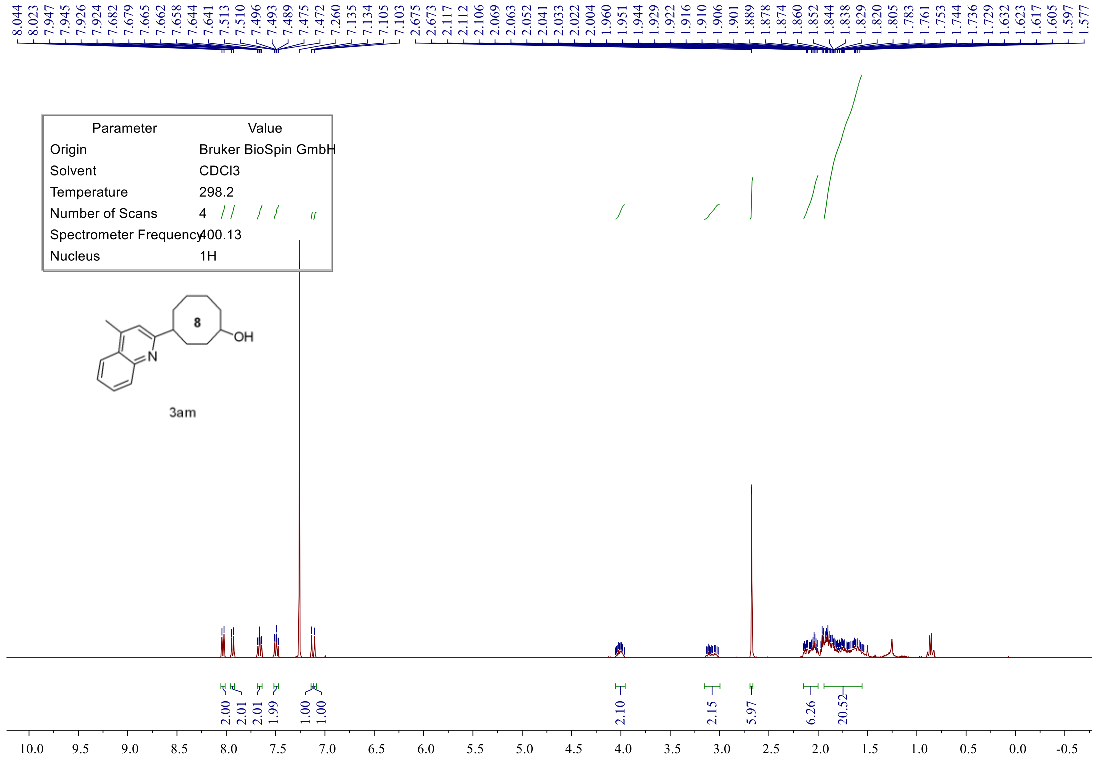

Supplementary Figure 87. ^{1}H **NMR (400 MHz,** $CDCl_3$**) spectra for compound 3am.**

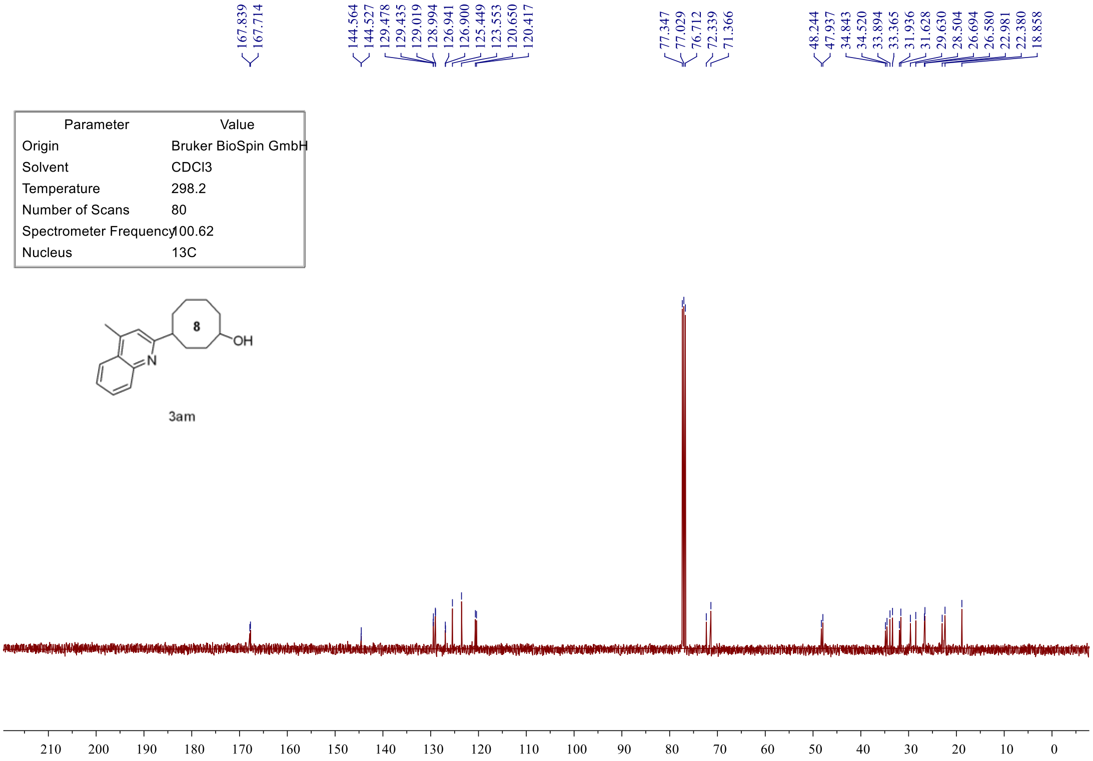

Supplementary Figure 88. ^{13}C NMR (100 MHz, $CDCl_3$) spectra for compound 3am.

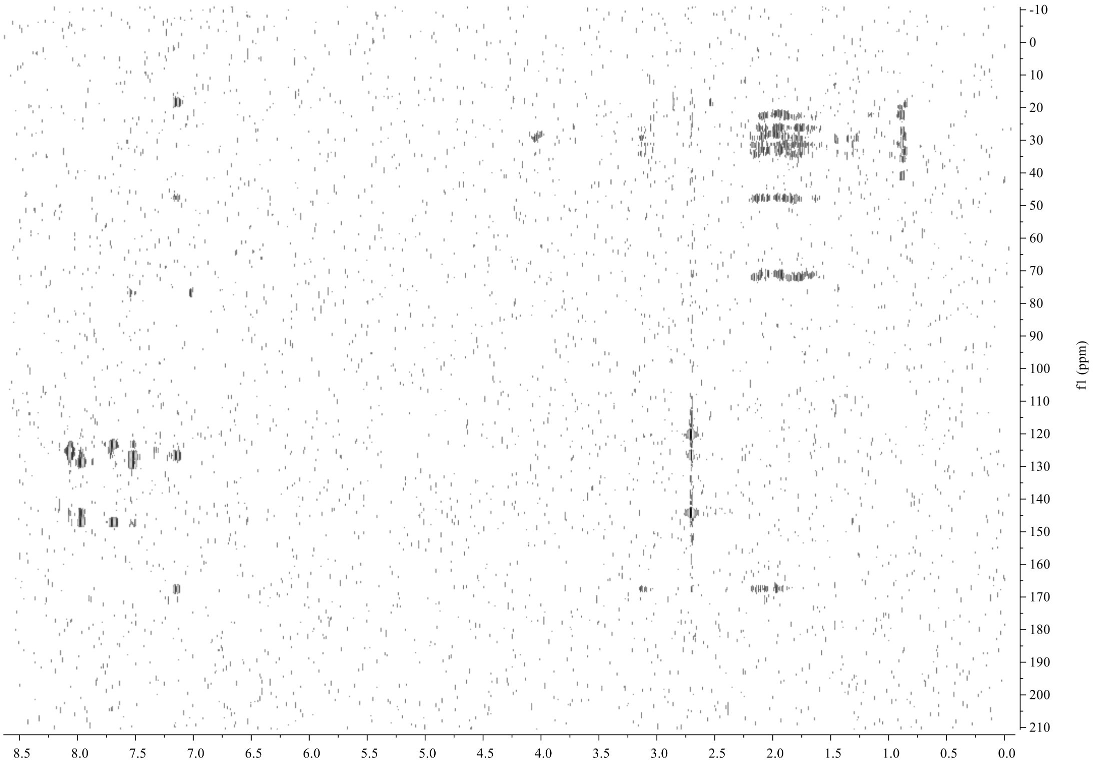

Supplementary Figure 89. HMBC spectra for compound 3am.

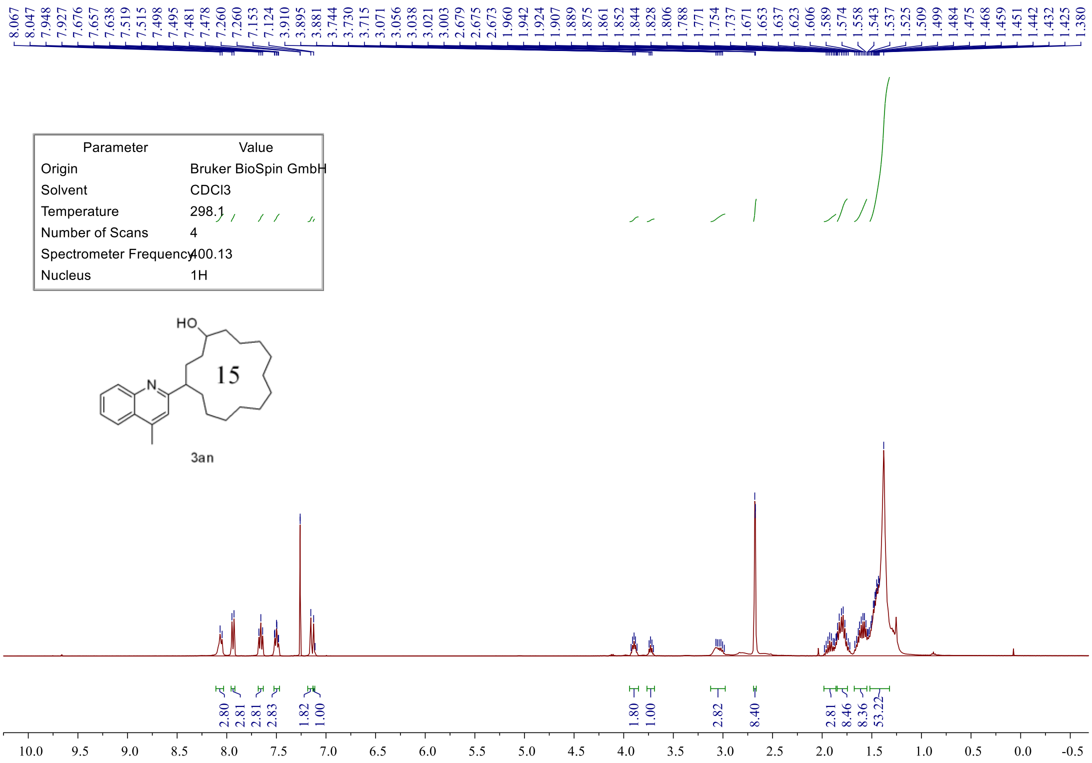

Supplementary Figure 90. ^{1}H NMR (400 MHz, $CDCl_3$) spectra for compound 3an.

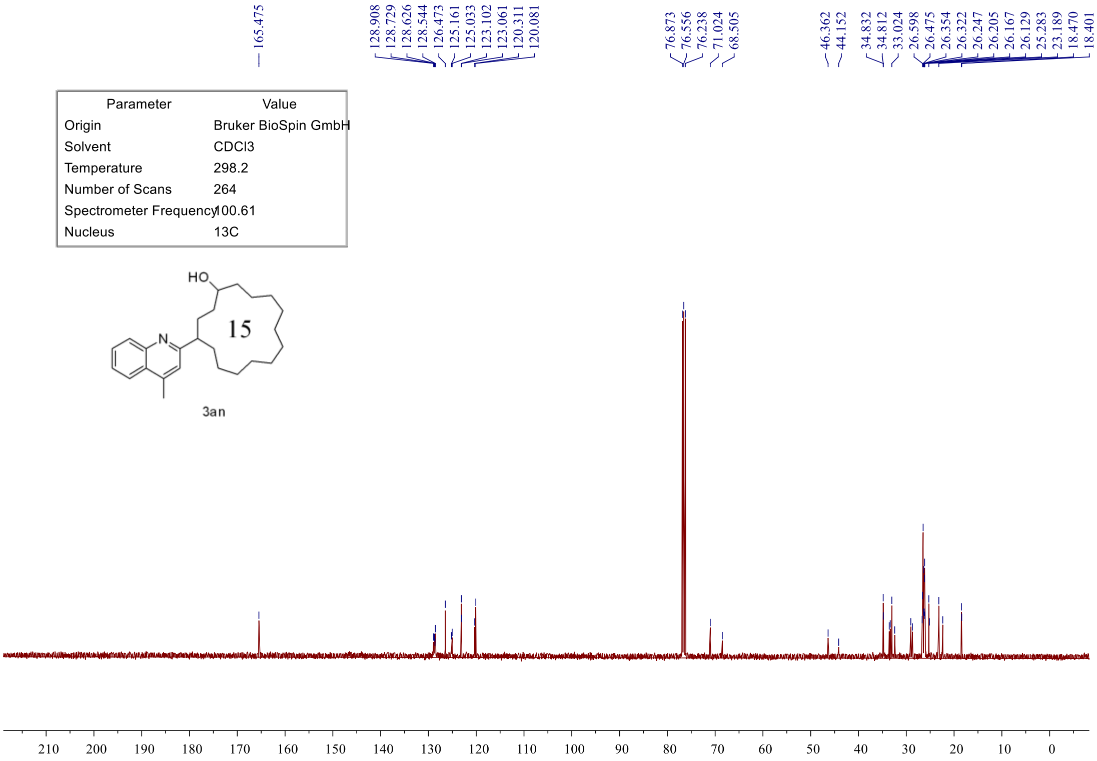

Supplementary Figure 91. ^{13}C NMR (100 MHz, $CDCl_3$) spectra for compound 3an.

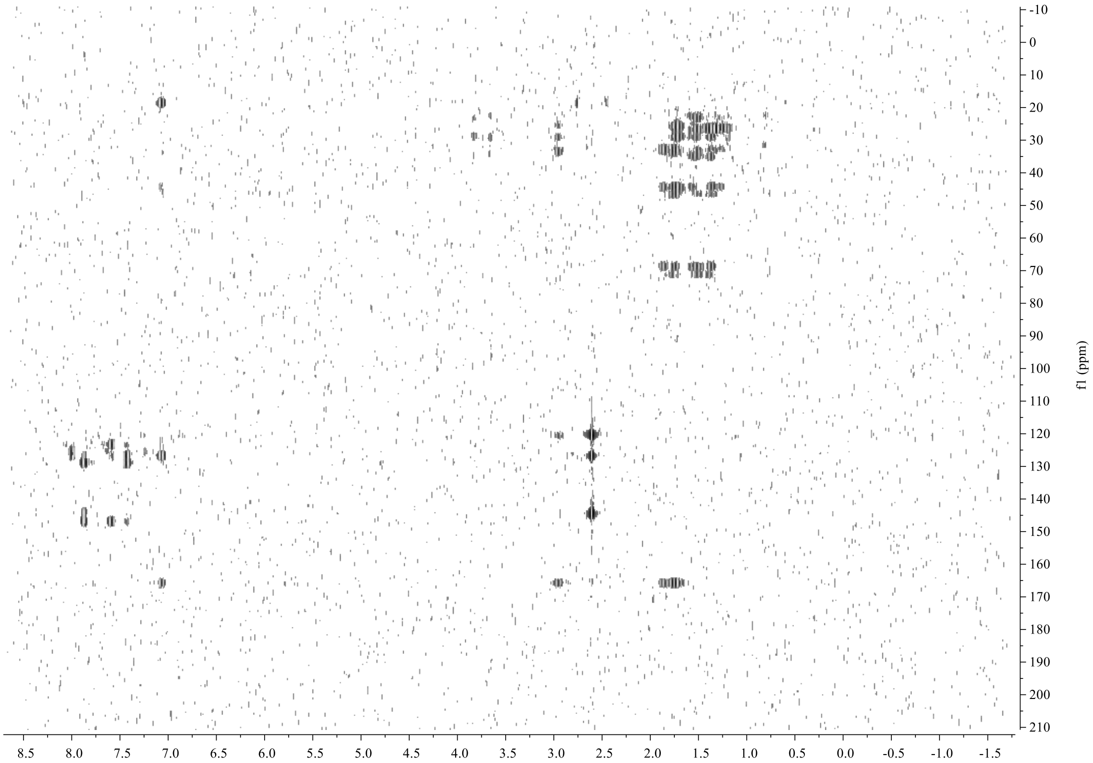

Supplementary Figure 92. HMBC spectra for compound 3an.

NMR experiments for mechanistic studies

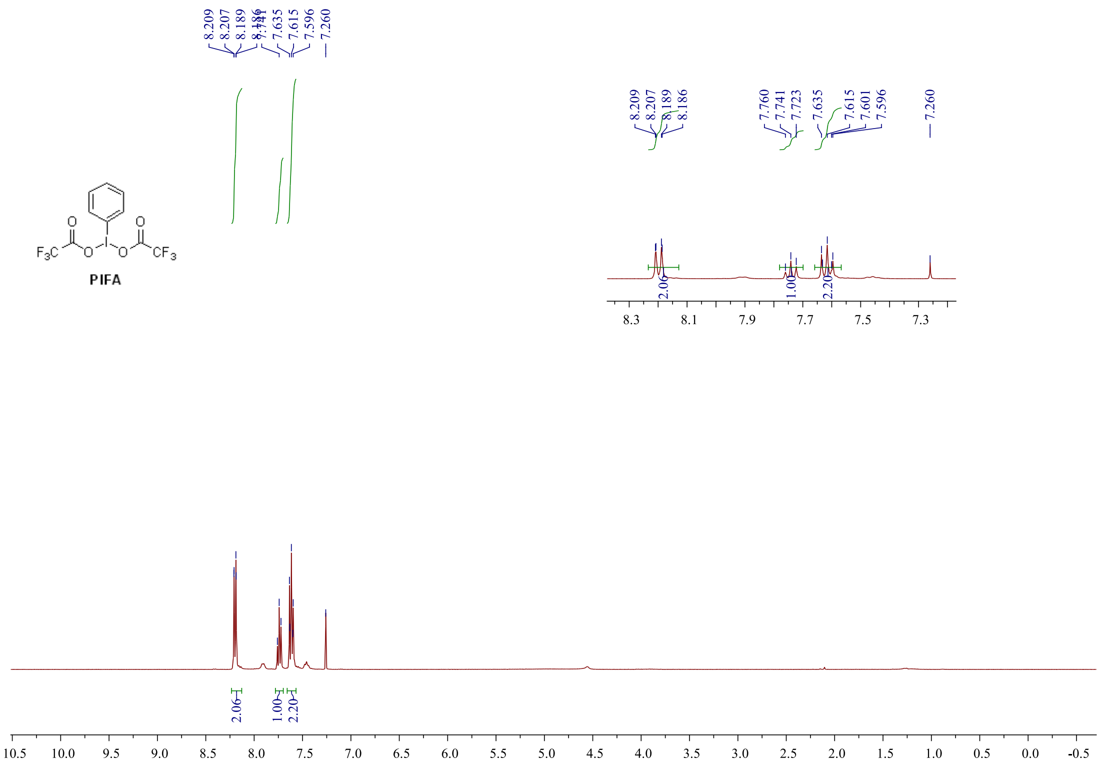

Supplementary Figure 93. PIFA-^{1}H NMR.

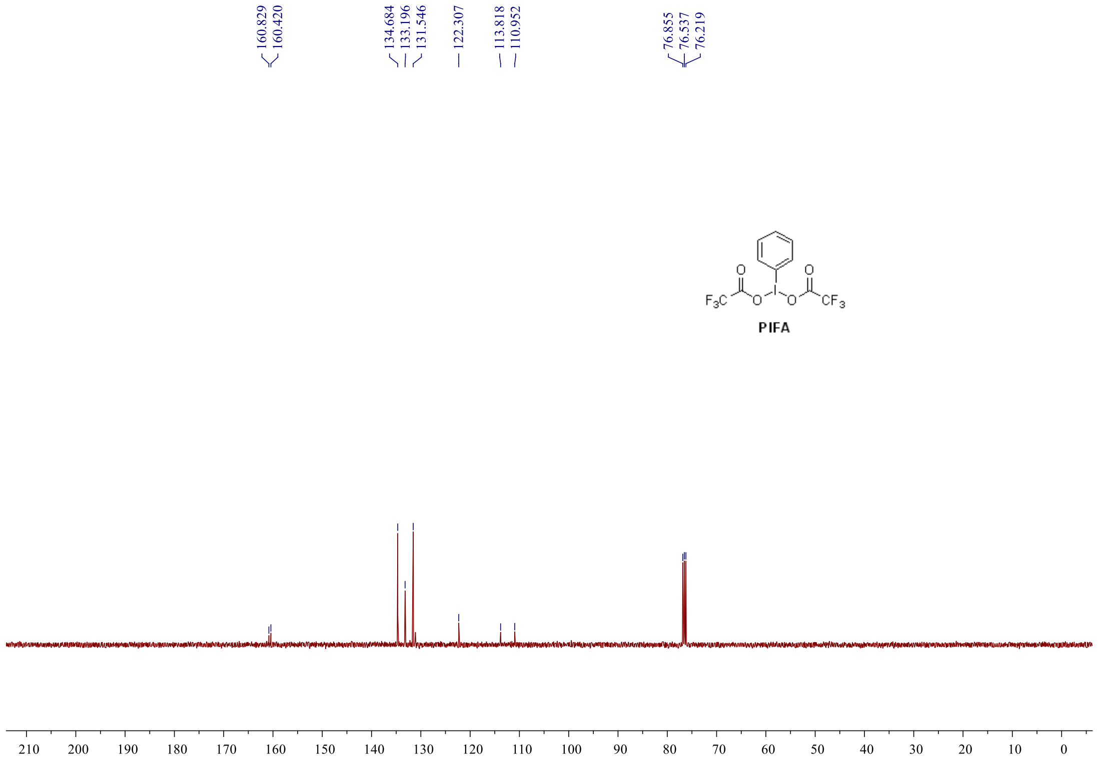

Supplementary Figure 94. PIFA-^{13}C NMR.

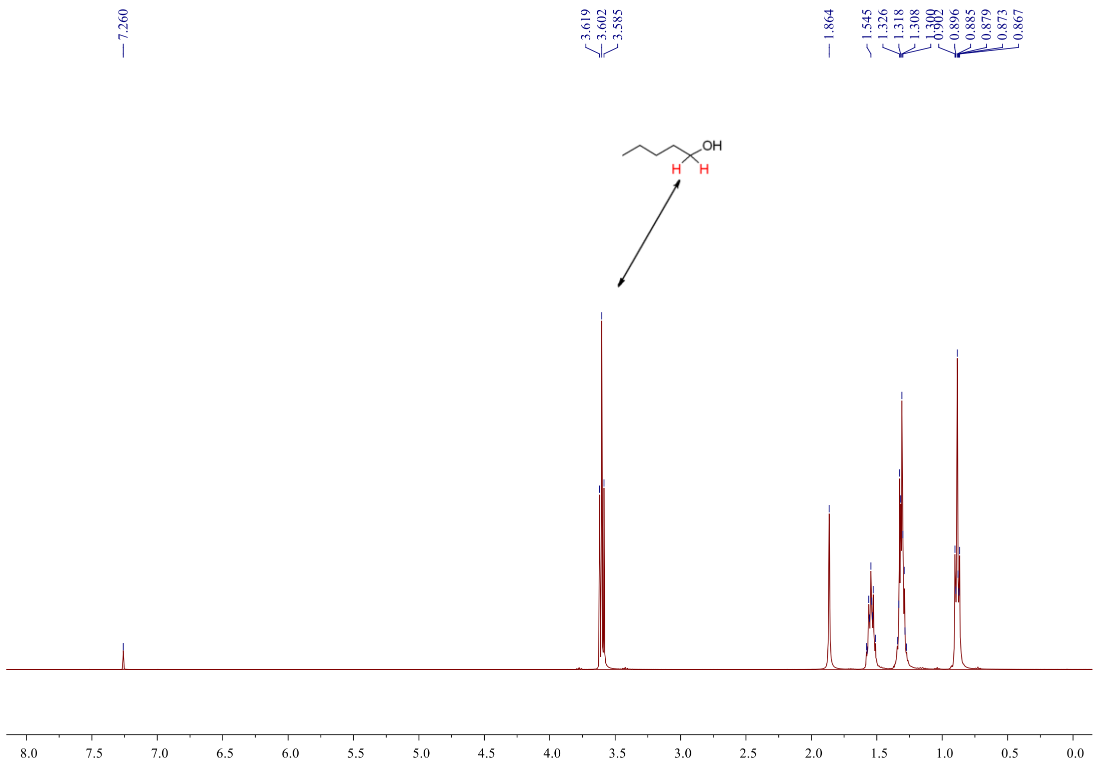

Supplementary Figure 95. *n*-Pentanol-^{1}H NMR.

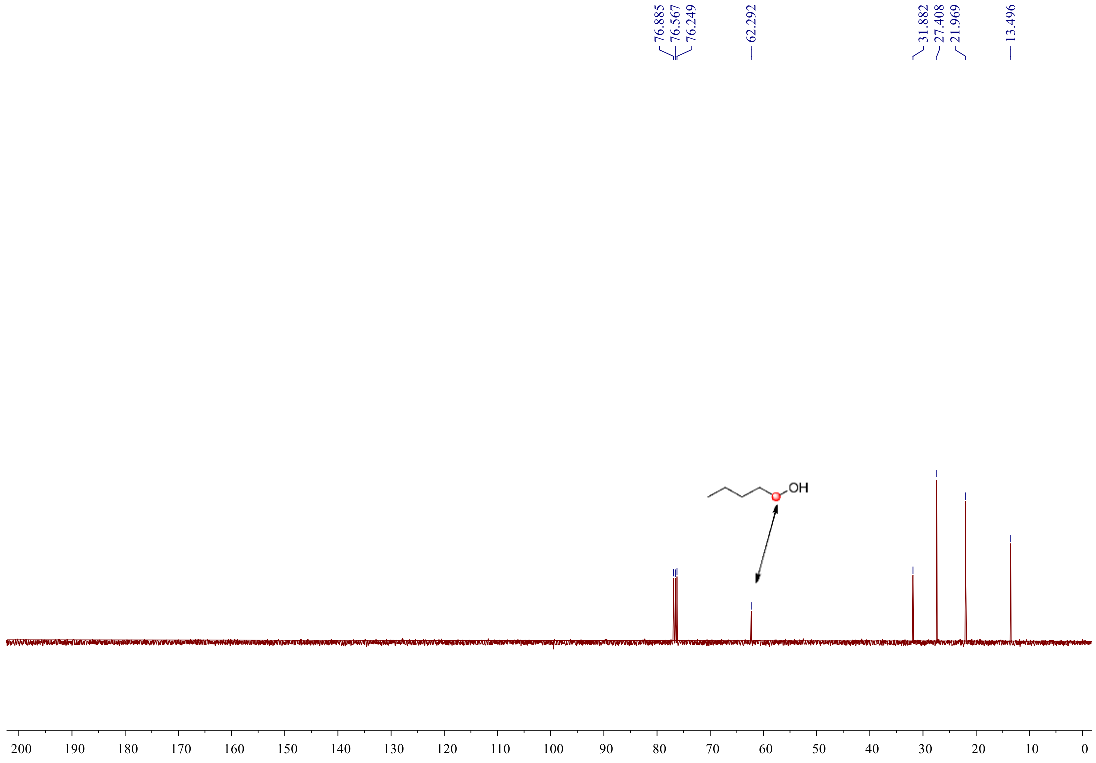

Supplementary Figure 96. *n*-Pentanol-^{13}C NMR.

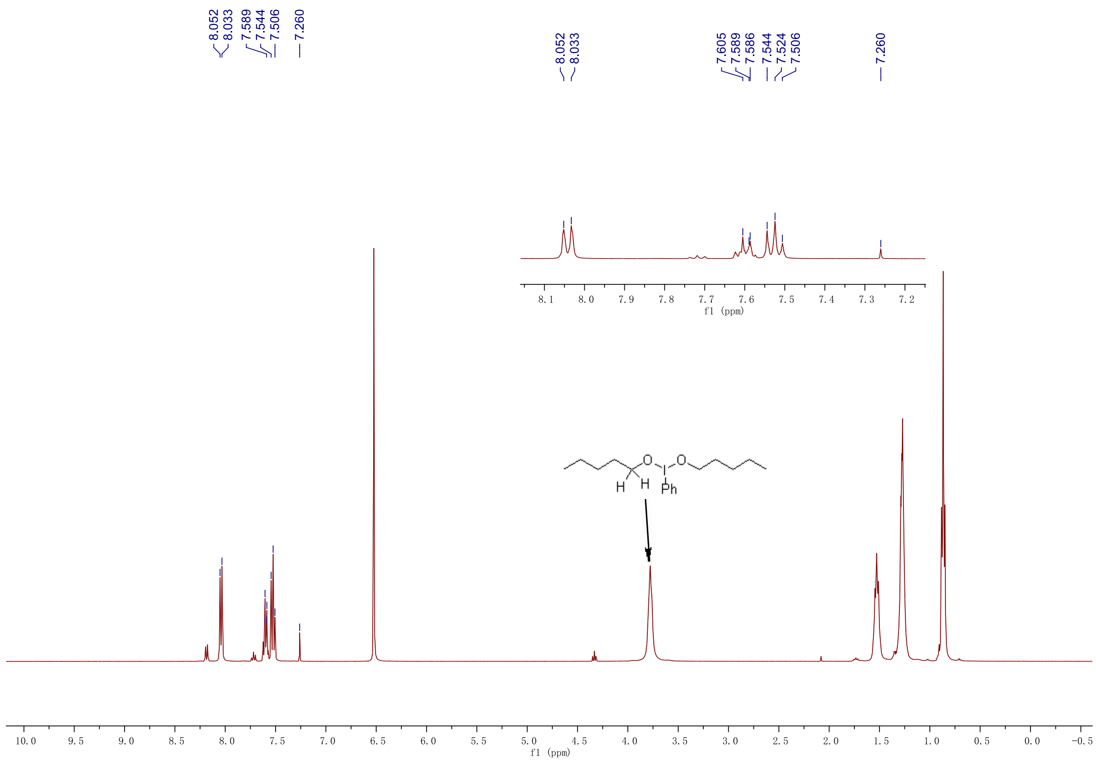

Supplementary Figure 97. Alcohol+PIFA (immediately) -^{1}H NMR.

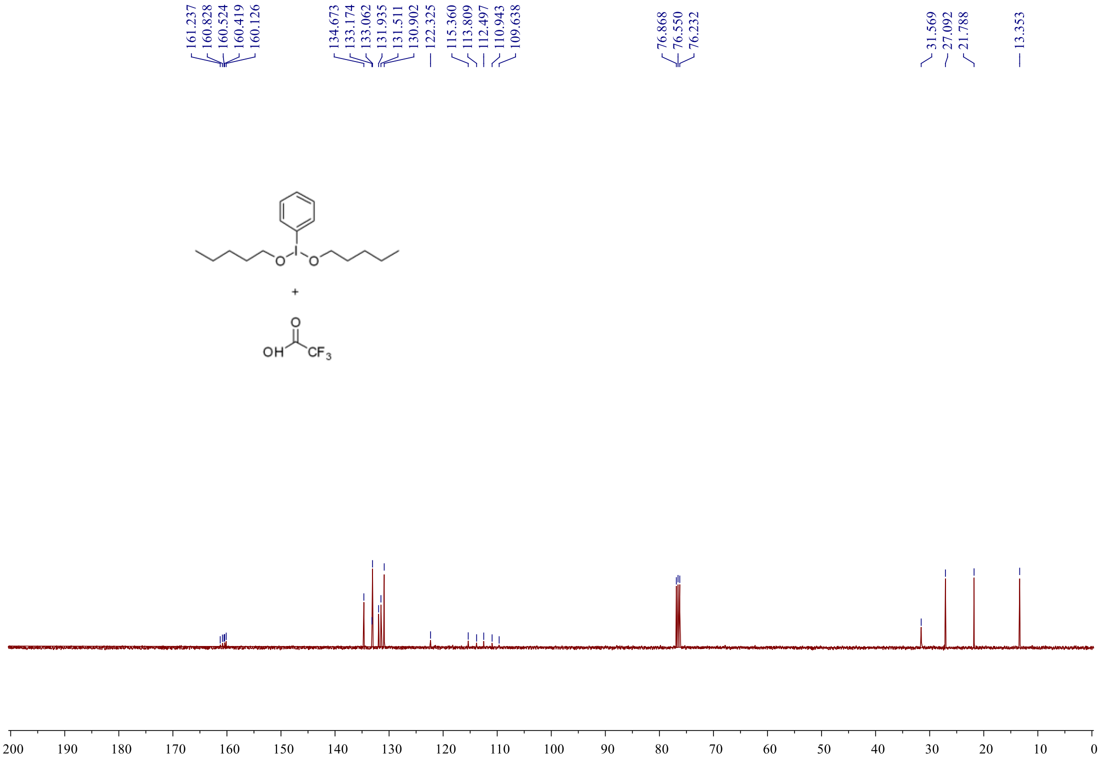

Supplementary Figure 98. Alcohol+PIFA (immediately) -^{13}C NMR.

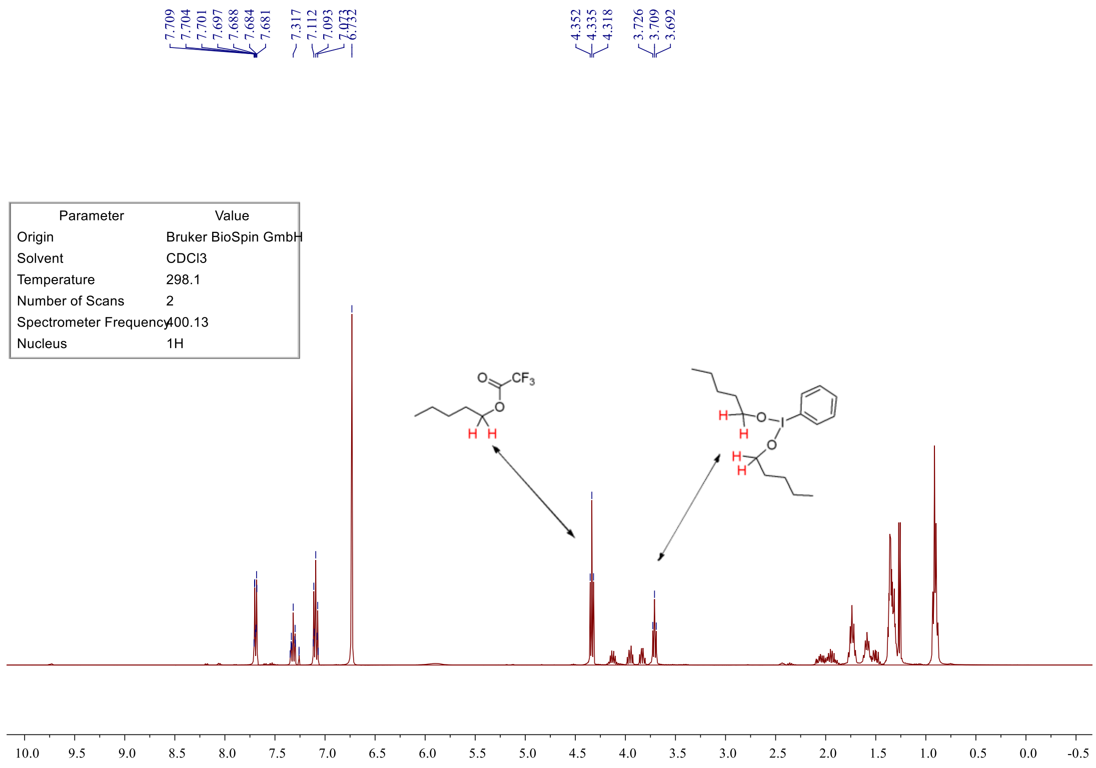

Supplementary Figure 99. Alcohol+PIFA (after 4 h irradiation) -^{1}H NMR.

Absorption spectra

Solutions of different complexes were introduced to a 1 cm path length quartz cuvette equipped with a Teflon® septum and analyzed using an Agilent Cary 5000 spectrophotometer.

For the solutions of 1-pentanol and PIFA in CH3CN: 1-pentanol (0.5 mmol) and PIFA (0.23 mmol) were dissolved in CH3CN (2 mL). The mixtures were stirred for 5 min, then transformed to 1 cm path length quartz cuvettes, sealed with Teflon® septa and degassed with a stream of argon for 10 minutes.

For the solutions of PIFA in CH3CN: PIFA (0.23 mmol) were dissolved in CH3CN (2 mL). The mixtures were stirred for 5 min, then transformed to 1 cm path length quartz cuvettes, sealed with Teflon® septa and degassed with a stream of argon for 10 minutes.

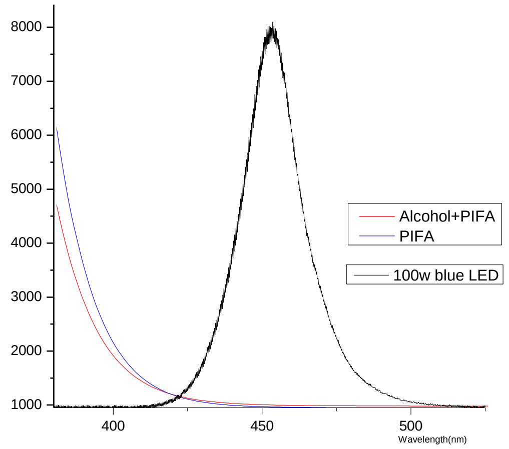

Supplementary Figure 100. Absorption spectra of the proposed $PhI(OC_5H_{11})_2$ and PIFA, and emission spectrum of blue LEDs

Radical clock experiment

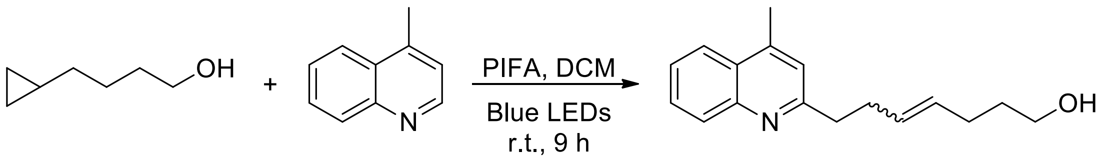

5 **1a** **6** (70 % yield, 4:1)

Supplementary Figure 101. Radical clock experiment

Heteroaryl **1a** (0.1 mmol) and alcohol **5** (0.5 mmol) were loaded in a reaction vial which was subjected to evacuation/ flushing with N_2 three times. Then DCM (2.0 mL) followed by PIFA (0.23 mmol) was added to the mixture. The reaction was irradiated with 100 W blue LEDs and kept at rt under fan cooling. After the reaction completion monitored by TLC, the mixture was quenched by addition of aq. KOH until pH>8 and then extracted with ethyl acetate (3 x 10 mL). The combined organic extracts were washed by brine, dried over Na_2SO_4, filtered, concentrated, and purified by flash column chromatography on silica gel (eluent: ethyl acetate/ petroleum ether) to give the desired product **6**.

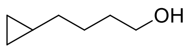

5: colorless oil. ^{1}H NMR (400 MHz, $CDCl_3$) δ 3.59 (t, *J* = 6.4 Hz, 2H), 2.21 (s, 1H), 1.60-1.51 (m, 2H), 1.46-1.37 (m, 2H), 1.18 (dd, *J* = 14.4, 7.2 Hz, 2H), 0.67-0.57 (m, 1H), 0.39-0.33 (m, 2H), -0.01--0.06 (m, 2H). 13C NMR (100 MHz, $CDCl_3$) δ 62.9, 34.5, 32.6, 25.8, 10.8, 4.3. FT-IR: ν (cm^{-1}) 3329, 3088, 3009, 2919, 2872, 2058, 1652, 1437, 1415, 1326, 1256. HRMS [ESI] calcd for $C_7H_{15}O$ $[M+H]^+$ 115.1117, found 115.1119.

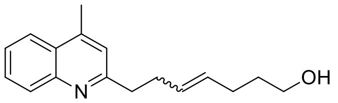

6 (ratio ~ 4:1): yellow oil. ^{1}H NMR (400 MHz, $CDCl_3$) δ 8.10-8.04 (m, 1.25H, two isomers), 7.97-7.92 (m, 1.25H, two isomers), 7.70-7.64 (m, 1.25H, two isomers), 7.53-7.48 (m, 1.25H, two isomers), 7.16-7.12 (m, 1.25H, two isomers), 5.58-5.35 (m, 2.5H, , two isomers), 3.61-3.55 (m, 2.5H, two isomers), 3.02-2.95 (m, 2.5H, two isomers), 2.69-2.66 (m, 3.75H, two isomers), 2.62-2.55 (m, 0.5H, single isomer), 2.53-2.46 (m, 2H, single isomer), 2.18-2.11 (m, 0.5H, single isomer), 2.09-2.02 (m, 2H, single isomer), 1.89 (br, 1.25H, two isomers), 1.63-1.51 (m, 2.5H, two isomers). ^{13}C NMR (100 MHz, $CDCl_3$) δ 161.8 (single isomer) & 161.7 (single isomer), 147.3 (two isomers), 144.6 (two isomers), 130.6 (two isomers), 130.1 (single isomer) & 129.6 (single isomer), 129.2 (single isomer) & 129.2 (single isomer), 129.0 (single isomer) & 128.9 (single isomer), 126.8 (two isomers), 125.7 (single isomer) & 125.6 (single isomer), 123.6 (two isomers), 122.3 (single isomer) & 122.2 (single isomer), 62.1 (single isomer) & 61.5 (single isomer), 39.0 (single isomer) & 38.8 (single isomer), 32.8 (two isomers), 32.3 (single isomer) & 32.2 (single isomer), 28.9 (single isomer) & 27.6 (single isomer), 23.4 (single isomer) & 18.7 (single isomer). FT-IR: ν (cm^{-1}) 2928, 2849, 2356, 2333, 1628, 1603, 1555, 1509. HRMS [ESI] calcd for $C_{17}H_{22}NO+$ $[M+H]^+$ 256.1696, found 256.1710.

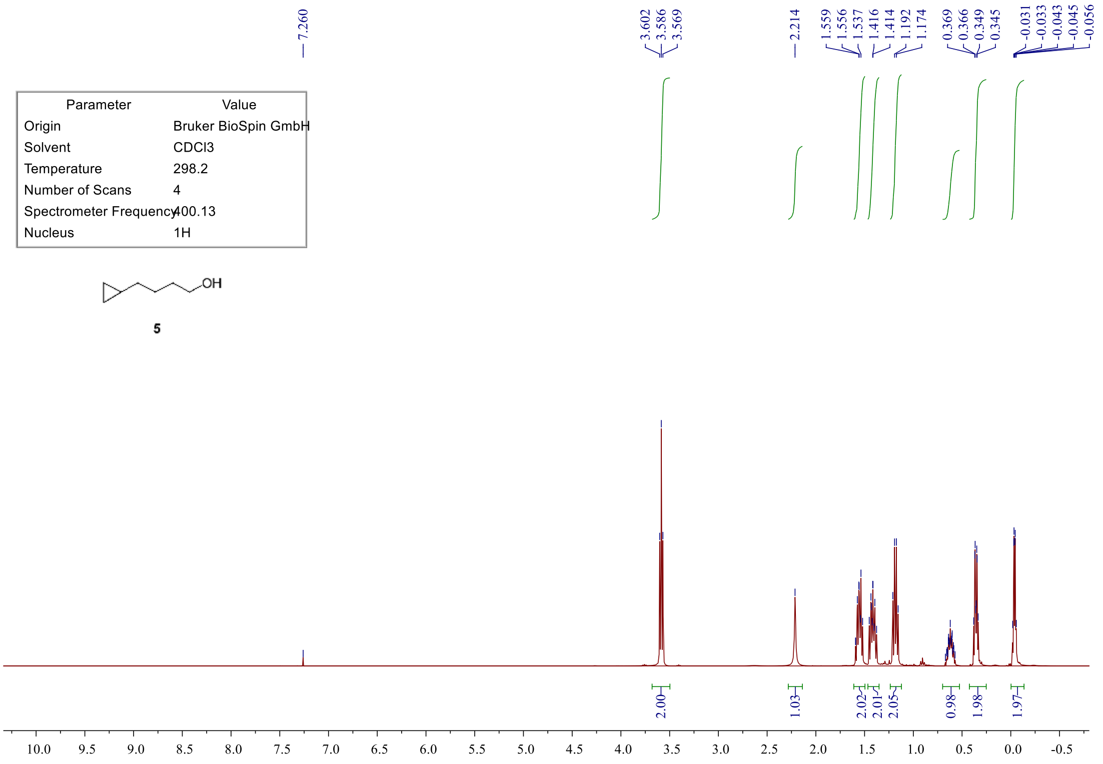

Supplementary Figure 102. ^{1}**H NMR (400 MHz,** $CDCl_3$**) spectra for compound 5.**

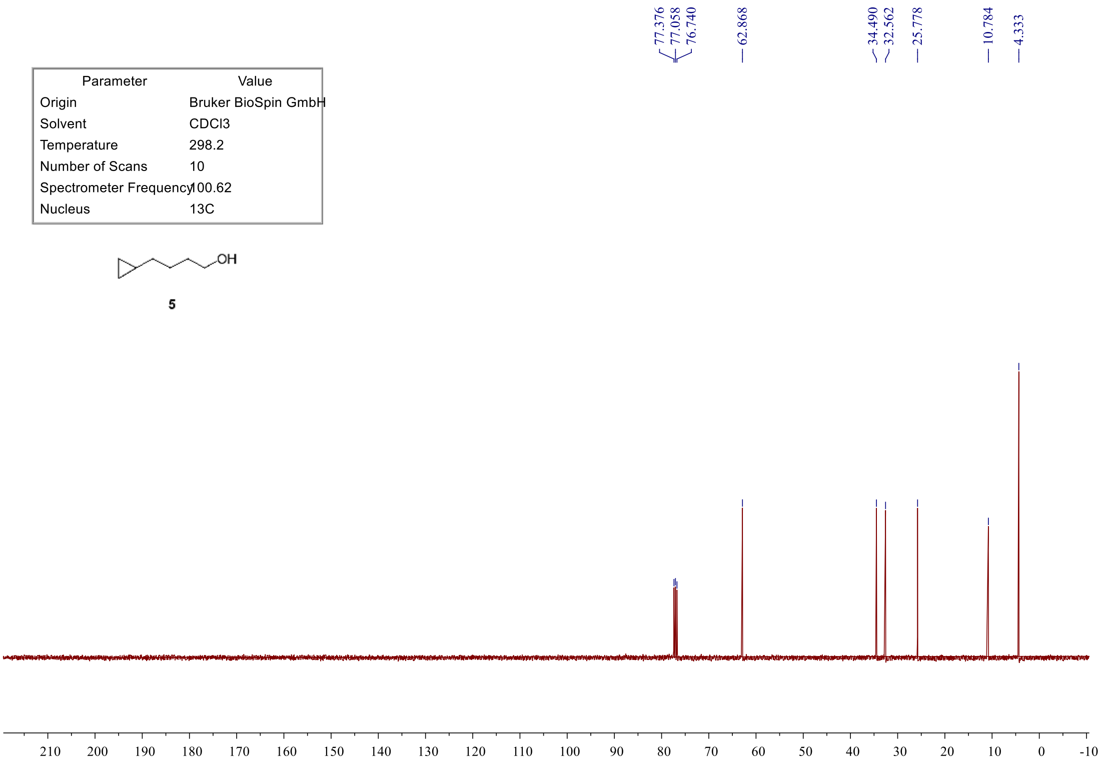

Supplementary Figure 103. ^{13}**C NMR (100 MHz,** $CDCl_3$**) spectra for compound 5.**

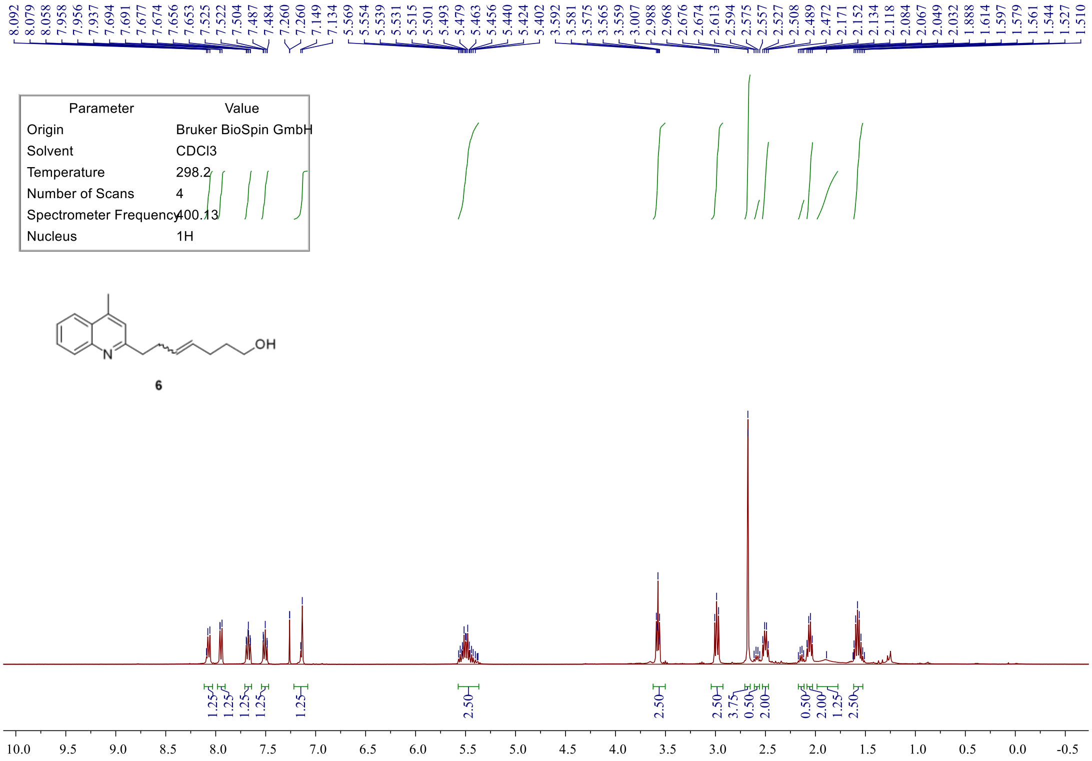

Supplementary Figure 104. ^{1}H NMR (400 MHz, $CDCl_3$) spectra for compound 6.

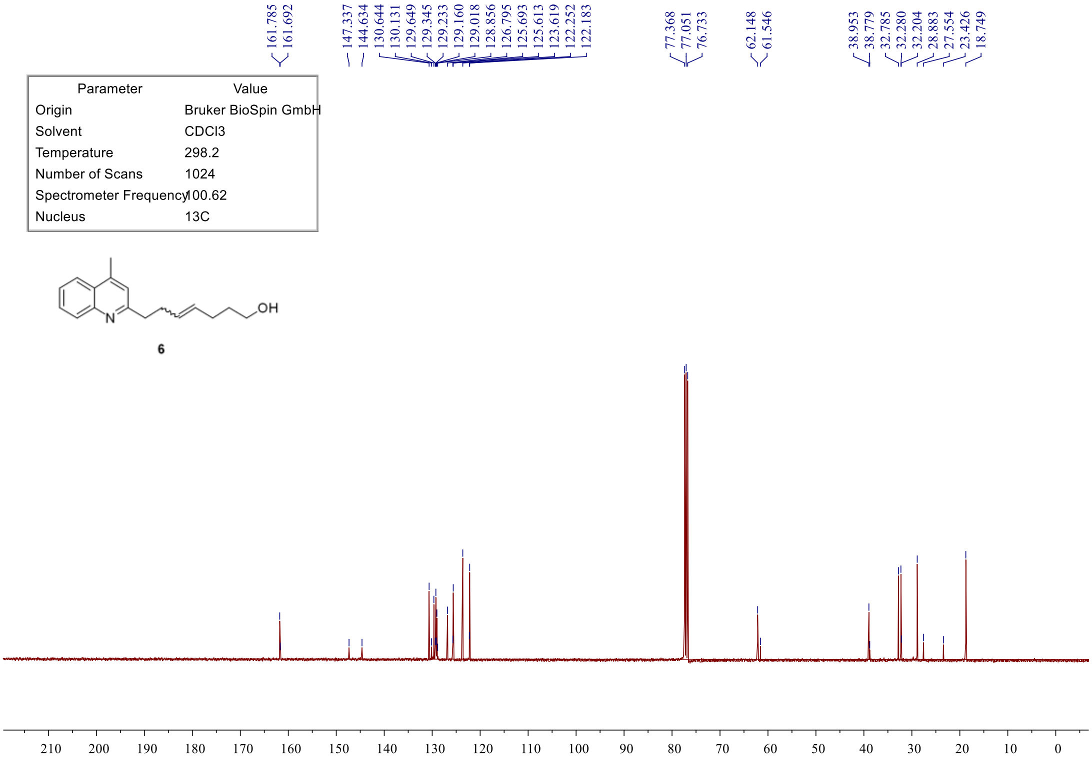

Supplementary Figure 105. ^{13}C NMR (100 MHz, $CDCl_3$) spectra for compound 6.
